# Development and Preclinical Evaluation of New Inhaled Lipoglycopeptides for the Treatment of Persistent Pulmonary Methicillin-Resistant Staphylococcus aureus Infections

**DOI:** 10.1128/AAC.00316-21

**Published:** 2021-06-17

**Authors:** Adam J. Plaunt, Sasha J. Rose, Jeong Yeon Kang, Kuan-Ju Chen, Daniel LaSala, Ryan P. Heckler, Arielle Dorfman, Barrett T. Smith, Donald Chun, Veronica Viramontes, Antonio Macaluso, Zhili Li, Yuchen Zhou, Lilly Mark, Jessica Basso, Franziska G. Leifer, Michel R. Corboz, Richard W. Chapman, David Cipolla, Walter R. Perkins, Vladimir S. Malinin, Donna M. Konicek

**Affiliations:** a Insmed Incorporated, Bridgewater, New Jersey, USA

**Keywords:** lipoglycopeptide, inhaled antibiotics, MRSA, intracellular infection, biofilm, cystic fibrosis

## Abstract

Chronic pulmonary methicillin-resistant Staphylococcus aureus (MRSA) disease in cystic fibrosis (CF) has a high probability of recurrence following treatment with standard-of-care antibiotics and represents an area of unmet need associated with reduced life expectancy. We developed a lipoglycopeptide therapy customized for pulmonary delivery that not only demonstrates potent activity against planktonic MRSA, but also against protected colonies of MRSA in biofilms and within cells, the latter of which have been linked to clinical antibiotic failure. A library of next-generation potent lipoglycopeptides was synthesized with an emphasis on attaining superior pharmacokinetics (PK) and pharmacodynamics to similar compounds of their class. Our strategy focused on hydrophobic modification of vancomycin, where ester and amide functionality were included with carbonyl configuration and alkyl length as key variables. Candidates representative of each carbonyl attachment chemistry demonstrated potent activity *in vitro*, with several compounds being 30 to 60 times more potent than vancomycin. Selected compounds were advanced into *in vivo* nose-only inhalation PK evaluations in rats, where RV94, a potent lipoglycopeptide that utilizes an inverted amide linker to attach a 10-carbon chain to vancomycin, demonstrated the most favorable lung residence time after inhalation. Further *in vitro* evaluation of RV94 showed superior activity to vancomycin against an expanded panel of Gram-positive organisms, cellular accumulation and efficacy against intracellular MRSA, and MRSA biofilm killing. Moreover, *in vivo* efficacy of inhaled nebulized RV94 in a 48 h acute model of pulmonary MRSA (USA300) infection in neutropenic rats demonstrated statistically significant antibacterial activity that was superior to inhaled vancomycin.

## INTRODUCTION

There are currently no approved therapies in the United States to treat chronic pulmonary methicillin-resistant Staphylococcus aureus (MRSA) infections in patients with cystic fibrosis (CF), a disease that affects approximately 25% of patients and has been associated with shortened life expectancy (1, 2). Front-line therapy for pulmonary MRSA in CF patients typically includes systemic administration of high-dose antibiotics, often the glycopeptide vancomycin, which has dose-limiting renal toxicity, requires intravenous administration, and does not exhibit sufficient sustained pulmonary levels to treat persistent infection (3–5). An investigative pulmonary-targeted approach designed to circumvent these drawbacks by formulating vancomycin into a dry powder for inhalation (DPI) recently reported negative results in a phase III clinical trial (NCT03181932). This outcome may not be surprising in light of a recent clinical investigation of a 28-day course of nebulized vancomycin in combination with standard-of-care antibiotics in the same patient population that showed insignificant improvement for the eradication of MRSA compared to a placebo-controlled group and demonstrated a greater incidence of bronchospasm (4).

Clinical failure of vancomycin for the treatment of persistent pulmonary MRSA when administered systemically or locally can be rationalized by its low permeability toward biological membranes, which limits its ability to colocalize with and subsequently act on protected colonies of MRSA that reside in biofilms and inside cells. Semisynthetic lipoglycopeptides, derivatives of vancomycin that modify the molecule with lipophilic side chains, provide a potential solution to this shortcoming because they possess enhanced lipophilicity and resultant membrane permeability characteristics that have enabled improved *in vitro* intracellular accumulation and biofilm efficacy (6–12). Moreover, lipoglycopeptides have demonstrated markedly enhanced antibacterial potencies versus planktonic colonies compared to vancomycin against a number of Gram-positive organisms, including MRSA, that can be attributed to additional antibacterial mechanisms of action (e.g., bacteriolysis), in addition to having lower propensities to form resistant mutants (13, 14).

A key challenge for the pharmaceutical development of lipoglycopeptide antibiotics is that they suffer from poor elimination kinetics *in vivo* (15). Chemical design approaches implemented to conserve high potencies of lipoglycopeptides and efficient membrane permeability often run counter to approaches to improve *in vivo* clearance. An example of this was highlighted during development of telavancin, where the synthetic intermediate *N*-decylaminoethylvancomycin, a molecule that we have recapitulated and refer to as RV40 herein, demonstrated highly potent *in vitro* activity but was found to accumulate in off-target tissues when administered parenterally to rodents (15). To address this finding, a hydrophilic functional group was incorporated at a different site on the vancomycin core, the resorcinol ring, to form telavancin, which resulted in reduced overall lipophilicity and off-target accumulation (15).

**FIG 1:**
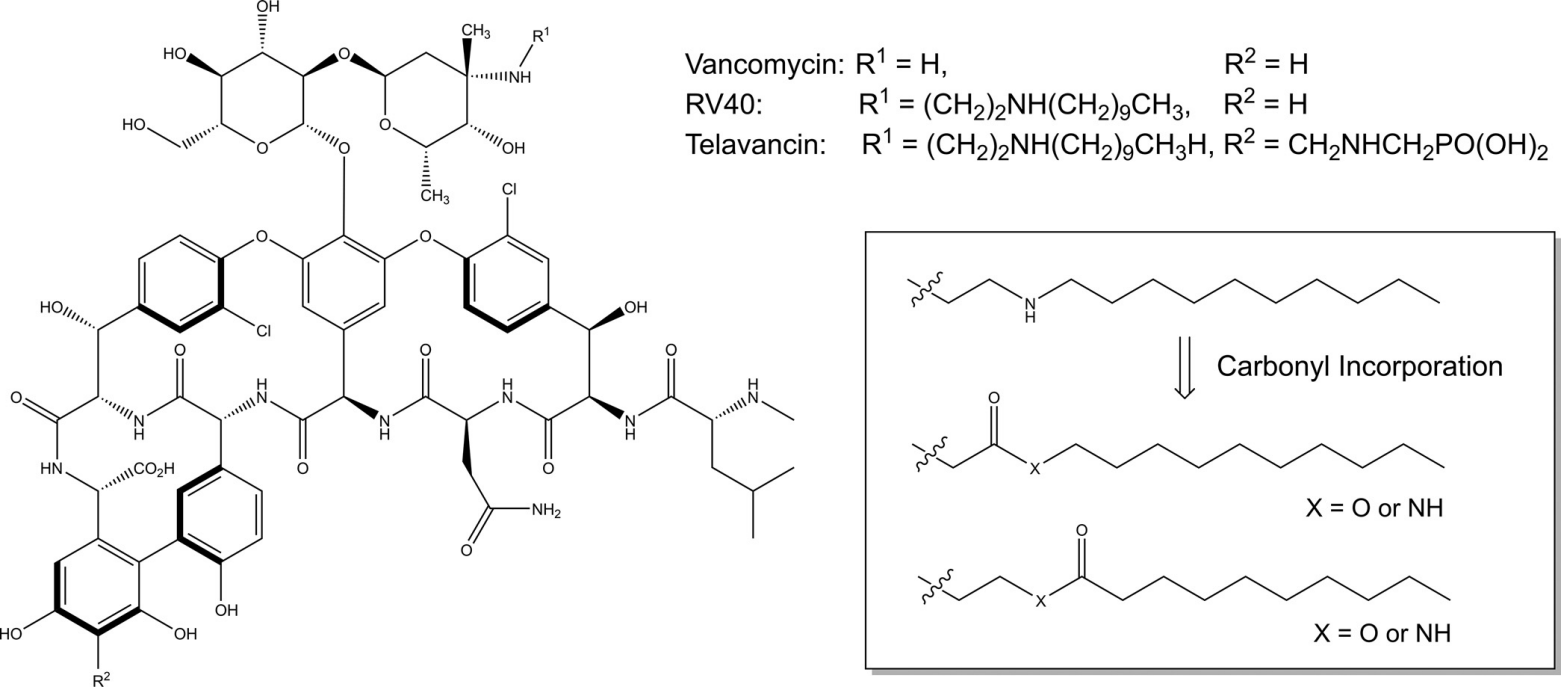
Structural comparison between vancomycin, RV40, and telavancin depicting a carbonyl incorporation strategy to develop the RV lipoglycopeptide antibiotic library.

Here, we sought to design and develop a lipoglycopeptide inhalation therapy superior to inhaled vancomycin for the treatment of chronic pulmonary MRSA infection that demonstrates high potency against planktonic, biofilm, and intracellular MRSA, the latter of which have been associated with clinical antibiotic failure (16, 17). Inhalation delivery of lipoglycopeptides to the lung requires different considerations than those for parenterally administered drugs, including evasion of first-pass metabolism pathways, deposition profiles that encourage access to the pathogen, and clearance from the respiratory tract (18–20). We hypothesized that by altering the hydrophobicity and charge of a vancomycin side chain, we could optimize the pharmacokinetics (PK) and pharmacodynamics (PD) for a more ideal inhaled therapeutic to support a chronic dosing regimen. In theory, we could design this to act either as a reverse prodrug that is subject to hydrolysis *in vivo*, resulting in a hydrophilic vancomycin-like by-product or a derivative thereof that is not efficiently hydrolyzed but demonstrates improved clearance due to optimized charge and hydrophobicity. Our concept, shown in Fig. 1 in relation to the lipoglycopeptides RV40 and telavancin, incorporates a labile carbonyl linker to extend the hydrophobic modification. This design platform enabled interrogation of a unique path to optimize the physiochemical properties of the molecule, resulting in improved *in vivo* PK relative to inhaled telavancin while concurrently conserving superior activity in comparison to vancomycin for the treatment of planktonic and protected populations of MRSA.

## RESULTS

### Chemistry.

RV lipoglycopeptide antibiotics were synthesized using standard synthetic chemistry techniques in accordance with literature precedent (15, 21). Specifically, a reductive amination strategy was used to synthesize all reported semisynthetic lipoglycopeptides (Scheme S1 in the supplemental material) (15, 21). Aldehyde precursors containing an oxygen or nitrogen heteroatom, a carbonyl, and differing alkyl length were obtained. Alkyl chain length varied from short octyl units (C8) to large palmitil units (C16), with chain length being inclusive of the carbonyl group when applicable. Note that a total of four available configurations can be formed with this combination of building blocks, two esters and two amides, with structural isomers (conventional or inverted) for each type of carbonyl bond.

### *In vitro* activity screening.

Activity screening for each derivative was determined by susceptibility testing of MRSA strain ATCC BAA 1556 (USA300) and the quality standard MSSA strain ATCC 29213, using broth microdilution, and the results were compared to RV40, telavancin, and vancomycin (Table 1). When vancomycin was modified using a conventional ester configuration, the most potent derivative was RV65, which bore a linear 12-carbon chain inclusive of the carbonyl group (i.e., C12 conventional ester) and yielded MIC values equal to 0.094 μg/ml for the MRSA ATCC BAA 1556 and 0.063 μg/ml for the MSSA ATCC 29213. Reconfiguration of the ester bond to yield the inverted ester configuration of RV compounds led to significantly diminished antimicrobial activity. The most potent inverted ester was RV55, which bore a linear 10-carbon chain (i.e., C10 inverted ester) and demonstrated MIC values of 0.25 μg/ml for both MRSA ATCC BAA 1556 and MSSA ATCC 29213.

**TABLE 1:**
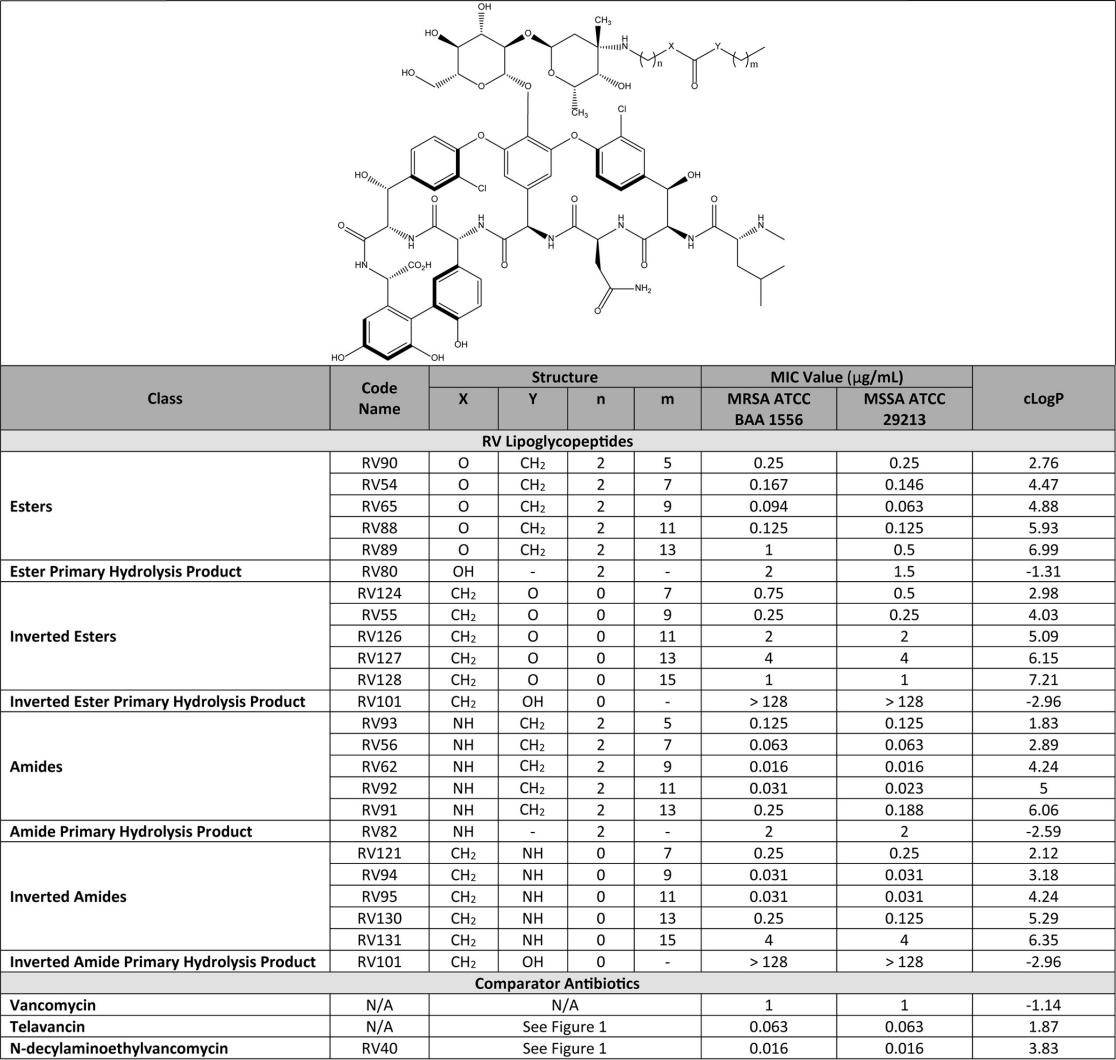
RV lipoglycopeptide and comparator antibiotic structure activity summary with cLogP values calculated using the ChemDraw software package

When using a conventional amide configuration, the most potent derivative was RV62, a C12 amide, which demonstrated MIC values of 0.016 μg/ml for both MRSA and MSSA. Unlike esters, reconfiguration of the amide bond to yield the inverted amide class of RV compounds did not lead to reduced antimicrobial activity. Two of the most potent RV lipoglycopeptides identified were the inverted amides RV94 and RV95 (C10 and C12 inverted amides, respectively) both with MIC values equal to 0.031 μg/ml for the MRSA and MSSA isolates investigated, which was approximately 30-fold more potent than vancomycin, 2-fold more potent than telavancin, and 2-fold less potent than RV40.

### *In vivo* PK screening.

Compounds representative of the various carbonyl attachment chemistries from the RV lipoglycopeptide library were selected for advancement into *in vivo* PK studies based on their *in vitro* potencies determined by MRSA MIC screening (Table 1) and propensities to hydrolyze (Fig. S1 and S2, and Table S1 in the supplemental material). The inverted ester configuration was not included due to its inferior potency by comparison to other configurations. Lung and plasma PK results for single-dose nebulized RV lipoglycopeptides and comparators administered by nose-only inhalation to healthy rats are summarized in Table 2 and plotted in Fig. S3. Inhaled vancomycin demonstrated efficient pulmonary elimination kinetics, with a half-life calculated to be 23 h. Telavancin, although previously reported to have a suitable clearance profile from plasma and lung when dosed parenterally (9, 15), demonstrated long elimination kinetics from lung tissue when dosed by inhalation (half-life = 507 h). Similarly, RV40, a synthetic intermediate of telavancin and potent antibiotic from the *in vitro* investigations herein, was not measurably eliminated from the lung over the course of the experiment (168 h).

**TABLE 2:** Pharmacokinetic parameters from single nebulized inhaled doses of RV lipoglycopeptides and comparators administered by nose-only inhalation to healthy rats^*d*^

Compound	Delivered dose (mg/kg)^*d*^	Pulmonary dose (mg/kg)^*d*^	Lung *C*_max_ (μg/g)^*d*^	Lung t_1/2_ (h)^*b*^^,^^*d*^	Lung AUC_0-∞_ (μg· h/g)^*d*^	Plasma *C*_max_ (μg/ml)	Lung: Plasma *C*_max_ ratio^*c*^	Drug (byproduct) lung level at 120 h (%)^*c*^
Vancomycin	10^*a*^	0.20	31	23	671	8.0	4	ND
Telavancin	0.4	0.03	12	507	3877	BLQ	ND	81
RV40	4.9	0.30	48	>333^*b*^	9800	0.10	690	105
RV88	23	0.12	26	21	360	0.02	1350	1.5 (71)
RV62	0.7^*a*^	0.15	30	11	317	0.02	1550	0.1 (107)
RV94	15	1.10	262	108	40671	0.14	1871	45 (0.1)

a Dose estimated from test article concentration and aerosol delivery flow rates due to insufficient filter sampling of the aerosol concentration at the time of the experiment.

b The RV40 lung half-life (t_1/2_) mean value was calculated as 5,140 h, with the 95% confidence interval (CI) lower range being 333 h.

c The RV lipoglycopeptide levels at 120 h postdose are relative to IPD levels, as are the primary hydrolysis by-products which are listed in parentheses. Telavancin, RV40, and vancomycin are not hydrolysable and therefore have no by-products listed. The vancomycin level was not determined (ND) at 120 h because the experiment concluded at 24 h. Telavancin lung: plasma *C*_max_ could not be determined because the plasma concentrations were BLQ (below limit of quantitation) at all time points. The limit of detection for drug concentrations in plasma was typically in the range of 1 to 20 ng/ml. PK plots can be seen in Fig. S3 in the supplemental material.

d The delivered dose is the dose presented to the nose of the animals during the inhalation procedure as determined by an aerosol sampling filter and subsequent analytical measurement of drug extracted from the filter (49). The pulmonary dose is the dose determined by direct measurement of compounds in the lung tissue. *C*_max_, t_1/2_, and AUC_0-∞_ were determined by noncompartmental extravascular analysis using PK solver 2.0 and applying the linear up/log down method.

The hydrolysable ester and amide-configured lipoglycopeptides RV88 and RV62 were efficiently eliminated from the lung after administration (half-lives = 21 h and 11 h, respectively); however, their primary hydrolysis by-products RV80 and RV82, respectively, were sustained in the lung throughout the course of the 120 h experiments (Table 2 and Fig. S3 inset). The lipoglycopeptide that demonstrated the most acceptable PK profile when given by inhalation was the inverted amide-linked lipoglycopeptide RV94, with a half-life calculated to be 108 h. Unlike the amide- and ester-linked lipoglycopeptides RV88 and RV62, which were fully converted to their hydrolysis products in the lung within 24 h, RV94 was minimally converted to its hydrolysis product RV101 with only 0.1% relative to the parent RV94 concentration at the maximum concentration of drug in lung tissue (*C*_max_). In addition, systemic concentrations of RV94 in the plasma were approximately 50-fold lower than plasma levels measured from a single inhaled dose of vancomycin, reducing the potential for systemic toxicities by comparison.

### Spectrum of activity.

**FIG 2:**
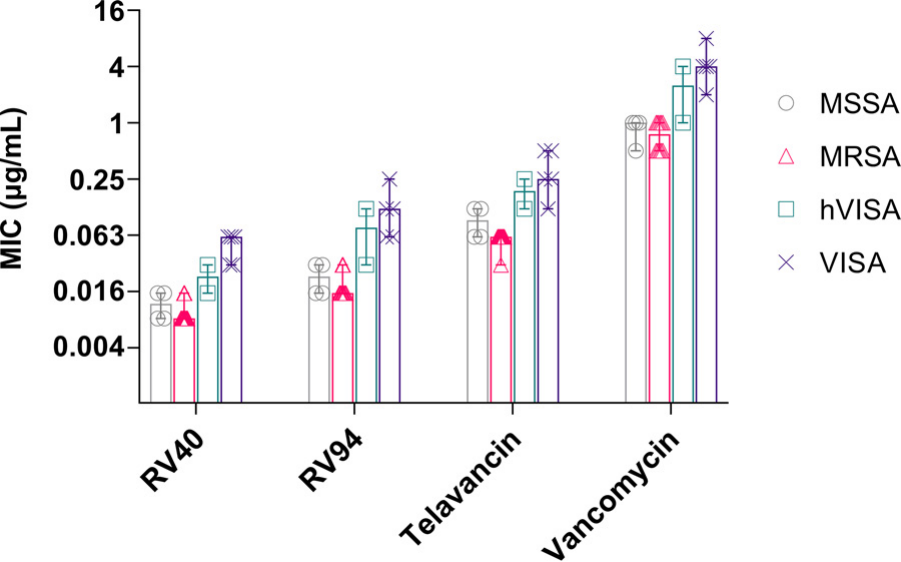
*In vitro* activities of RV94 and select comparators against antibiotic-susceptible and -resistant forms of S. aureus as determined by broth microdilution. MIC, minimum inhibitor concentration. Data plotted as median MIC and error bars are range. Methicillin-susceptible S. aureus (MSSA), *n* =  4 isolates; methicillin-resistant S. aureus (MRSA), *n* =  11 (RV94) or 12 isolates; heterogeneous vancomycin-intermediate S. aureus (hVISA), *n* =  2 (RV94) or 3 isolates; vancomycin-intermediate S. aureus (VISA), *n* =  5 isolates.

Due to its potent *in vitro* activity and its superior *in vivo* PK performance in comparison to compounds of its class, RV94 was advanced into more comprehensive testing against an expanded panel of 74 Gram-positive isolates and the results are summarized in Table 3 and listed for specific organisms in Table S2. Further, to highlight antimicrobial potencies of the test compounds versus strains associated with the disease indication discussed herein, the data summarizing compound activities against methicillin-susceptible and -resistant forms of S. aureus are plotted in Fig. 2. Against MSSA and MRSA, RV94 demonstrated highly potent antimicrobial activity, with MICs ranging from 0.015 to 0.03 μg/ml. By comparison to the other antibiotics investigated in this panel, RV94 was on average 1- to 2-fold less potent than the highly potent RV40, 4- to 8-fold more potent than telavancin, and 30- to 60-fold more potent than vancomycin. Against vancomycin-intermediate S. aureus (VISA), RV94 demonstrated median MICs equal to 0.08 μg/ml for heterogeneous vancomycin-intermediate S. aureus (hVISA) and 0.12 μg/ml for VISA, a 3- and 2-fold activity reduction compared to RV40, a 2-fold enhancement compared to telavancin for both strains, and an 18- and 30-fold enhancement in comparison to vancomycin. RV94 was tested against vancomycin-resistant S. aureus and showed moderate activity, with a median MIC equal to 4 μg/ml, in comparison to vancomycin which had MICs of >64 μg/ml. These activity data for RV94 against vancomycin-intermediate and -resistant forms of S. aureus support the claim of additional modes of action, as has been reported previously for telavancin and the lipoglycopeptide class (14).

**TABLE 3:** Summary of RV94 and comparator *in vitro* microbiological activity against 74 Gram-positive organisms determined by broth microdilution

Organism	Type^*a*^	No. of isolates	RV40^*b*^		RV94		Telavancin		Vancomycin	
			MIC (μg/ml)	MIC:MBC	MIC (μg/ml)	MIC:MBC	MIC (μg/ml)	MIC:MBC	MIC (μg/ml)	MIC:MBC
Gram-positive aerobes										
S. aureus	MSSA	4	0.012	1	0.023	8	0.09	1	0.75	1
S. aureus	MRSA	12	0.008	1	0.015	1	0.06	1	0.5	1
S. aureus	hVISA	2	0.023	1	0.08	8	0.19	1	1.5	1
S. aureus	VISA	5	0.06	1	0.12	2	0.25	1	4	4
S. aureus	VRSA	12	NT	NT	4	NT	NT	NT	>64	NT
Staphylococcus epidermidis	MSSE	1	0.015	NT	0.015	NT	0.12	NT	0.5	NT
S. epidermidis	MRSE	2	0.008	1	0.03	1	0.12	1	1	1
Staphylococcus lugdunensis		1	0.004	NT	0.015	NT	0.06	NT	0.25	NT
Staphylococcus haemolyticus		1	0.015	NT	0.06	NT	0.06	NT	1	NT
Staphylococcus hominis		1	0.015	NT	0.03	NT	0.06	NT	0.5	NT
Enterococcus faecalis	VSE	2	0.023	>8	0.03	>8	0.19	>8	1.5	>8
E. faecalis	VanA VRE	1	0.5	8	0.03	>8	1	8	128	NT
Enterococcus faecium	VSE	1	0.004	NT	0.015	NT	0.06	NT	0.5	NT
E. faecium	VanA VRE	1	1	>4	2	>8	2	>4	128	NT
E. faecium	VanB VRE	1	0.008	>8	0.03	>8	0.06	>8	64	>2
Streptococcus pneumoniae	PISP	2	0.004	1	0.006	4	0.02	1	0.6	8
S. pneumoniae	PRSP	1	≤0.008	NT	0.008	NT	0.03	NT	0.25	NT
Streptococcus pyogenes		2	0.04	NT	0.012	NT	0.06	NT	0.25	NT
S. pyogenes	erm^R^	1	0.008	1	0.008	1	0.06	1		1
Streptococcus agalactiae		2	0.008	1	0.02	4	0.06	>8	0.25	4
S. agalactiae	erm^R^	1	0.008	NT	0.004	NT	0.06	NT	0.25	NT
Streptococcus dysgalactiae		3	0.015	1	0.008	4	0.12	4	0.25	>8
Streptococcus anginosus	AGS	1	0.015	>8	0.015	>8	0.06	>8	0.5	>8
Streptococcus constellatus	AGS	1	0.008	NT	0.008	NT	0.03	NT	0.25	NT
Streptococcus mitis	MGS	2	0.02	1	0.008	1	0.05	1	0.25	1
Streptococcus oralis	MGS	1	0.015	NT	0.015	NT	0.06	NT	0.5	NT
Gram-positive anaerobes										
Clostridioides difficile	toxAB-	1	0.06	NT	0.015	NT	0.12	NT	0.25	NT
C. difficile	ribo 027	1	0.06	NT	0.015	NT	0.12	NT	0.5	NT
C. difficile	NAP1;ribo 027	1	0.12	NT	0.06	NT	0.12	NT	1	NT
Clostridium. perfringens		1	0.015	NT	0.008	NT	0.015	NT	0.25	NT
Peptostreptococcus micros		1	0.06	NT	NT	NT	0.12	NT	NT	NT
Peptostreptococcus anaerobius		1	0.008	NT	0.008	NT	0.03	NT	0.12	NT
Cutibacterium acnes		2	0.008	NT	0.008	NT	0.02	NT	0.19	NT
Eggerthella lenta		1	NT	NT	0.008	NT	NT	NT	0.5	NT

a MSSA, methicillin-susceptible S. aureus; MRSA, methicillin-resistant S. aureus; hVISA, heterogeneous vancomycin-intermediate S. aureus; VISA, vancomycin-intermediate S. aureus; VRSA, vancomycin-resistant S. aureus, MSSE, methicillin-susceptible S. epidermidis; MRSE, methicillin-resistant S. epidermidis; VSE, vancomycin-susceptible enterococci; VanA VRE, VanA-type vancomycin-resistant enterococci (vancomycin- and teicoplanin-resistant); VanB VRE, VanB-type vancomycin-resistant enterococci (vancomycin-resistant and teicoplanin-susceptible); PISP, penicillin-intermediate S. pneumoniae; PRSP, penicillin-resistant S. pneumoniae; erm^R^, erythromycin-resistant; AGS, anginosus group streptococci; MGS, mitis group streptococci; NT, not tested.

b MIC listed in the cells is the median MIC value. The MBC was measured on one organism and type except for MRSA, where the MBC represents the median of *n* = 5, and VISA, where it represents the median of *n* = 2. MIC, minimum inhibitory concentration; MBC, minimum bactericidal concentration.

RV94 demonstrated similar potent activities when tested against a select panel of Gram-positive aerobes that included various susceptible and resistant enterococci and streptococci with MICs ranging from 0.004 to 2 μg/ml and Gram-positive anaerobes that included *Clostridium*, *Peptostreptococcus*, *Propionibacterium*, and *Eggerthella*, with median MICs ranging from 0.008 to 0.06 μg/ml. Consistent with the glycopeptide class, it was expected that RV94 and RV40 would not demonstrate activity against the Gram-negative organisms. Against Pseudomonas aeruginosa and Burkholderia cepacia, RV40 was not active, demonstrating MICs >64 μg/ml (Table S2). RV40 demonstrated moderate activity against Acinetobacter baumannii (32 to 64 μg/ml), Haemophilus influenzae (16 to 32 μg/ml), and Moraxella catarrhalis (1 to 2 μg/ml).

### *In vitro* cellular accumulation and intracellular MRSA killing.

**FIG 3:**
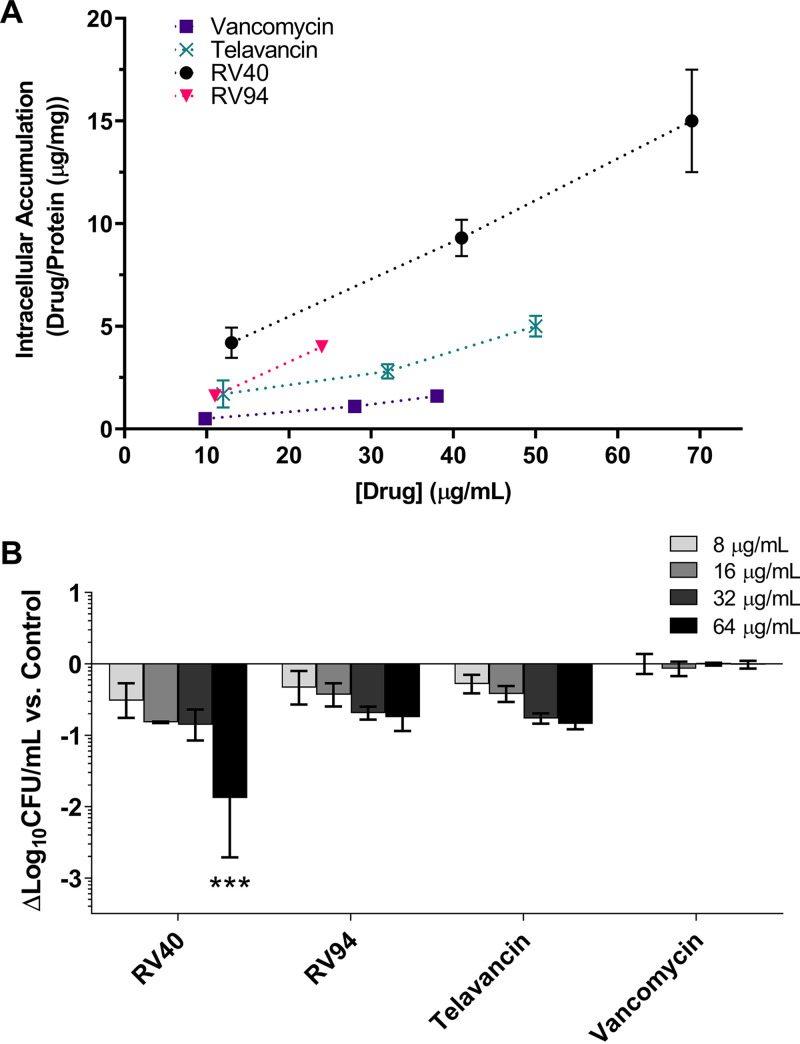
*In vitro* accumulation and MRSA killing of RV94 and comparators in THP-1 cells. (A) Cellular accumulation of RV compounds and comparators in THP-1 cells. Cells were treated with test compounds for 24 h. Data are plotted as means ± standard deviation (SD) (*n* = 3 per time point). (B) *In vitro* intracellular activity of RV compounds against intracellular MRSA ATCC BAA 1556 (USA300) in THP-1 cells. Cells were infected with MRSA for 1 h at MOI equal to 10. Incubation with lysostaphin (25 mg/ml) was done for 2 h to eliminate residual extracellular bacteria. Wells were washed with PBS, replaced with medium containing test compounds and 150 nM bafilomycin A1 (47), and incubated for 24 h. After treatment, cells were lysed and surviving intracellular bacteria were enumerated. Log_10_ CFU reduction for each concentration of respective test compound relative to the log_10_ CFU count of untreated cells is presented. The average untreated cell MRSA count was 4.9 ± 0.5 log_10_ CFU/ml (*n* = 3). Each ml of medium contained 7.5 × 10^5^ cells. Data are plotted as averages ± SD; *n* = 3 (RV40, telavancin, and vancomycin) or *n* = 2 (RV94) per experiment, with each experiment having *n* = 3 measurements. Limit of detection (LOD) = 2.0 log_10_ CFU/ml. Statistics are based on two-way ANOVA with Bonferroni's multiple-comparison test. *P* = 0.0005 for RV40 (64 μg/ml) versus control.

The cellular accumulation of RV94 and comparators in THP-1 cells is shown in Fig. 3A. RV94 demonstrated cellular accumulation that was similar to RV40 and superior to vancomycin and telavancin, with a 2- and 4-fold improvement, respectively, at concentrations tested in the range of 10 to 75 μg/ml. The intracellular MRSA (ATCC BAA 1556; USA300) killing activity of the RV94 and comparators are plotted as a function of log_10_ CFU/ml versus control in THP-1 cells (Fig. 3B). RV40, RV94, and telavancin demonstrated nearly dose-dependent intracellular MRSA killing that was superior to vancomycin. RV40 demonstrated the greatest activity against intracellular MRSA of the compounds investigated, with up to a 1.9 log_10_ CFU/ml reduction in MRSA titer compared to untreated control cells at the highest dose investigated (64 μg/ml). The intracellular killing activity of RV94 was similar to telavancin, with both compounds demonstrating a 0.8 log_10_ CFU/ml reduction in MRSA titer at the 64 μg/ml treatment dose. Notably, this treatment concentration exceeds the solubility limit of RV94 (ca. 50 μg/ml) but not that of RV40 (ca. 150 μg/ml), which could affect compound activities under the *in vitro* testing conditions. Vancomycin had no effect on reducing the intracellular MRSA titer versus control at doses up to 64 μg/ml, which is consistent with our hypothesis and motivation for designing the RV compound library.

### *In vitro* biofilm MRSA killing.

**FIG 4:**
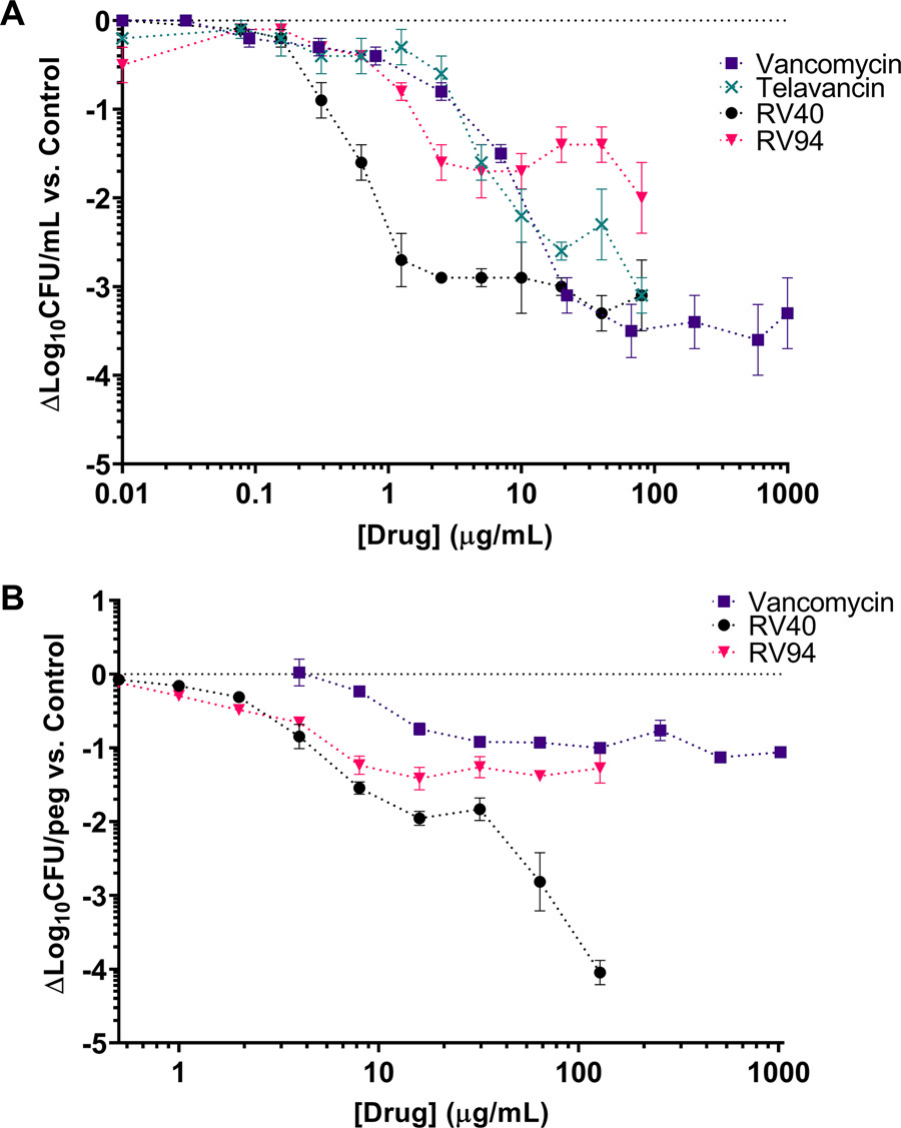
RV94 and comparator *in vitro* biofilm activity against MRSA ATCC BAA 1556 (USA300). Statistical analysis is based on two-way ANOVA with Bonferroni's multiple-comparison test. Asterisks designating statistical significance were omitted from the plots for clarity. (A) *In vitro* activity against a simple MRSA biofilm conducted in a static microtiter plate with minimum essential media (MEM). MRSA biofilms (at an inoculum of 7.7 log_10_ CFU/ml) were formed in plastic microtiter plates, as detailed in the Materials and Methods section and described previously (48). At 6 h postinoculation, test compounds were added to the biofilm culture and then incubated for another 16 h before disruption. The disrupted biofilms were collected and processed for CFU enumeration. Data are plotted as the mean change in log_10_ CFU/ml reduction versus untreated biofilm. Error is the standard error of the mean (SEM) for *n* = 4 (vancomycin, RV94) or *n* = 3 (telavancin, RV40) per experiment, with triplicate measurements in each experiment. Untreated MRSA biofilm average titer = 7.2 ± 0.1 log_10_ CFU/ml (*n* = 17). LOD = 2.6 log_10_ CFU/ml. Statistical significance was as follows for drug-treated versus control biofilms: RV40 for concentrations ≥1.25 μg/ml (*P* = 0.002 at 1.25 μg/ml); telavancin for concentrations ≥10 μg/ml (*P* = 0.02 at 10 μg/ml); vancomycin for concentrations ≥22 μg/ml (*P* = 0.0008), and no statistical significance was observed for the RV94 treatment. (B) *In vitro* activity of RV lipoglycopeptides in comparison to vancomycin against a more complex MRSA biofilm using the MBEC assay system. MRSA biofilms (at an inoculum of 7.7 log_10_ CFU/ml) were developed in TSB with the addition of 1% human plasma for 24 h. The biofilms established on the peg lid were challenged with test compounds in MHIIB broth for another 24 h. Biofilm disruption was performed by two sonication cycles and further processed for CFU enumeration. Data are plotted as the mean change in log_10_ CFU/peg reduction versus untreated biofilm. Error is SEM for *n* = 1 experiment for each drug with triplicate measurements. Untreated MRSA biofilm average titer = 6.2 log_10_ CFU/peg. LOD = 1.1 log_10_ CFU/peg. Statistical significance was as follows for drug-treated versus control biofilms: RV94 for concentrations ≥2 μg/ml (*P* = 0.04 at 2 μg/ml); RV40 for concentrations ≥4 μg/ml (*P* < 0.0001 at 4 μg/ml); vancomycin for concentrations ≥16 μg/ml (*P* = 0.0007 at 16 μg/ml).

The MRSA killing activity of RV lipoglycopeptides and comparator antibiotics against a static MRSA biofilm in minimal essential medium (MEM) is plotted in Fig. 4A. The RV compounds demonstrated potent activity (1 to 2 log_10_ CFU/ml reductions in MRSA titer versus control) at doses in the range of 0.625 to 2.5 μg/ml, which was on the order of 2- to 11-fold more potent than telavancin and vancomycin at equivalent treatment doses. RV40 demonstrated superior activity, with an approximate 3 log_10_ CFU/ml reduction in MRSA biofilm titer versus control when treated with doses ranging from 1 to 10 μg/ml, while vancomycin, RV94, and telavancin were less potent, yielding approximately 2 log_10_ CFU/ml reductions in this dose range. Apart from RV94, all compounds tested yielded reductions in MRSA biofilm titer that were bactericidal (≥3 log_10_ CFU/ml versus control) at doses of ≤80 μg/ml. While RV94 demonstrated bacteriostatic activity at low doses, yielding a 1 log_10_ CFU/ml reduction versus control at 2.5 μg/ml, this effect diminished nonlinearly at the higher doses investigated, where bactericidal activity was no longer achieved with this compound; again, this could be explained by its low solubility in testing medium (ca. 50 μg/ml).

RV94 *in vitro* biofilm activity was then evaluated in a more complex model using the standardized MBEC assay (22) conducted in tryptic soy broth (TSB) with the addition of 1% human plasma, which can augment biofilms to become more tolerant to antibiotic treatment (Fig. S4 and reference 23). In this model (Fig. 4B), RV94 had a maximum 1.2 log_10_ CFU/peg reduction in MRSA titer at a dose equal to 16 μg/ml, which was similar to that for RV40 and 2-fold more potent than vancomycin at the same dose, and the effect leveled off at doses up to 128 μg/ml. Vancomycin achieved a maximum 1.1 log_10_ CFU/peg reduction in titer relative to control at 512 μg/ml. Notably, RV40 was the most potent compound investigated in this assay and achieved a 4 log_10_ CFU/peg reduction compared to untreated biofilm at a dose equal to 128 μg/ml. Again, the activities of compounds at the higher doses investigated could be affected by their solubility under testing conditions.

### *In vivo* efficacy.

**FIG 5:**
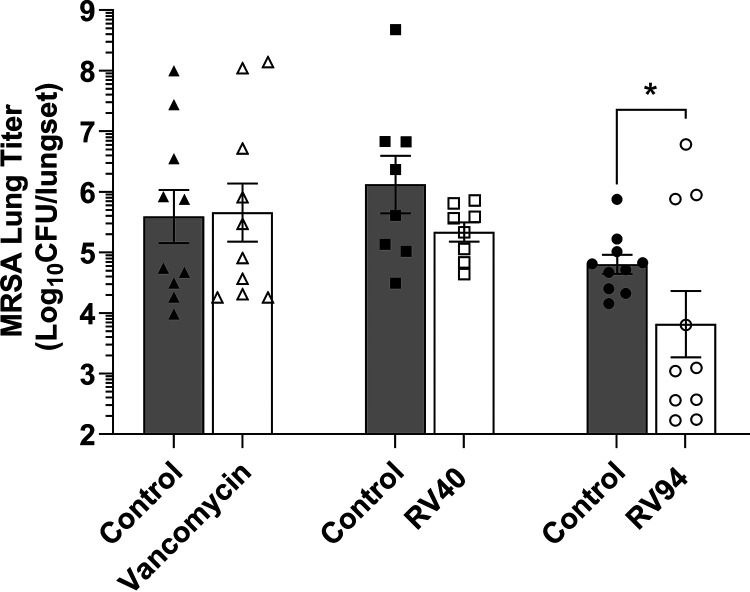
*In vivo* efficacy of RV94 and RV40 versus vancomycin when administered by nebulized nose-only inhalation in an acute pulmonary MRSA (ATCC BAA 1556; USA300) infection in neutropenic rats. Rats were rendered neutropenic with intraperitoneal (i.p.) cyclophosphamide on day −4 (150 mg/kg) and day −1 (100 mg/kg) relative to the challenge. On day 0 at 0 h, rats were challenged with MRSA via intranasal instillation at a target titer of 8.0 to 8.3 log_10_ CFU. At 24 and 32 h postchallenge, rats were loaded into a 12-port inhalation dosing tower, where they received nebulized drug or vehicle control treatments by nose-only inhalation. Animals were euthanized at 48 h postinfection for enumeration of lung MRSA titer. MRSA lung titer of test article treatments versus control is plotted for three separate experiments. Bars are geometric mean and error is SEM; *n* = 10 (vancomycin and control, RV94 and control) or 8 (RV40 and control) per group. Control is inhaled saline (vancomycin) or bicine (solvent for RV40 and RV94). The average of two doses delivered at 24 and 32 h postchallenge for vancomycin was 49.8 ± 0.6 mg/kg, for RV40 the average was 14.4 ± 2.6 mg/kg, and for RV94 it was 18.8 ± 0.7 mg/kg. Statistics are based on the Kolmogorov-Smirnov test for drug treatment versus control (RV94, *P* = 0.01). LOD = 2.1 log_10_ CFU/lungset.

The *in vivo* efficacy of inhaled nebulized RV94 in comparison to inhaled nebulized RV40 and vancomycin was investigated in an acute pulmonary MRSA infection in neutropenic rats. Treatments were administered 24 h and 32 h postinfection and the animals were euthanized at 48 h postinfection for enumeration of lung MRSA titer. In Fig. 5, the data demonstrate that both RV40 and RV94 treatments yielded reductions in lung MRSA versus control, whereas vancomycin was not effective when administered at doses that were 2.5- to 3.5-fold higher than the lipoglycopeptides. Inhaled vancomycin demonstrated an average 0.1 log_10_ CFU increase in lung MRSA titer versus inhaled vehicle control and RV40 demonstrated an average 0.8 log_10_ CFU reduction versus vehicle control. Inhaled RV94 was the most potent of the treatments investigated and demonstrated statistical significance from control (Kolmogorov-Smirnov test, *P* = 0.01), with an average 1.0 log_10_ CFU reduction in pulmonary MRSA titer.

## DISCUSSION

We sought to design a targeted inhalation therapy to treat persistent pulmonary MRSA infection in CF patients that demonstrates high potency against planktonic, biofilm, and intracellular MRSA, the latter two of which have been linked to clinical relapse after conventional therapy with front-line treatment options such as intravenous vancomycin (16, 17). The RV compound library of semisynthetic lipoglycopeptide derivatives was developed by modifying vancomycin at its vancosamine position using several different carbonyl linking configurations to extend various acyl chain lengths (Table 1). The goal of this work was to conserve the high potencies and membrane-penetrating characteristics of lipoglycopeptides while ameliorating shortcomings associated with their poor elimination kinetics *in vivo* (15). Moreover, we aimed to deliver the product as an inhalation therapy, which would be a novel route of administration for the lipoglycopeptide class of antibiotics. This required additional chemical modifications to optimize pulmonary PK and create an effective therapy that can be chronically administered without accumulation.

The first objective of this effort was to select for high antibacterial potency to minimize the inhaled efficacious dose and corresponding treatment burden for patients. In Table 1, the MIC activities for the RV lipoglycopeptide library are shown for MRSA ATCC BAA 1556 (USA300) and the quality control standard MSSA ATCC 29213. Therein, and consistent with the lipoglycopeptide class, are examples from each of the four carbonyl configurations investigated that demonstrated marked improvements in antibacterial activity compared to vancomycin. For the most potent compounds, this difference was on the order of 30-fold to 60-fold. Within the RV compound series, the activities show dependence on both the total length of the hydrophobic side chain and the configuration of the carbonyl bond. For each configuration, the optimal alkyl chain length was approximately 10 to 12 carbons after the peptide bond, inclusive of the carbonyl group, which is consistent with previous reports of alkyl vancomycin derivatives (24). Moreover, the cLogP value, a measure of the compound’s hydrophobicity, was in the range of 3.2 to 4.9 for the most potent compounds (Table 1). These data indicate that RV lipoglycopeptides with shorter aliphatic side chains do not possess sufficient lipophilicities to support additional mechanisms of action (e.g., bacteriolysis) that are characteristic to the class and thought to be responsible for enhanced potencies relative to traditional glycopeptides (11, 24). In contrast, RV lipoglycopeptides with longer side chains possess hydrophobicities that could limit their solubilities in testing media, which leads to reduced antibacterial activity *in vitro*. In terms of carbonyl bond configuration, RV lipoglycopeptides prepared with amide and inverted amide functionality exhibited superior *in vitro* activity among the series with select compounds that were 2- to 4-fold more potent than the lipoglycopeptide telavancin. Moreover, these configurations demonstrated lower propensities to hydrolyze compared to the ester-linked counterparts *in vitro*, which supported further investigation in more complex *in vitro* and *in vivo* models (Fig. S1 and S2; Table S1).

After establishing potent *in vitro* activity of the RV lipoglycopeptides against MRSA, we proceeded to evaluate their *in vivo* PK in healthy rats in pursuit of a suitable clearance profile supportive of a chronic dosing regimen when delivered by nose-only inhalation, and compared the results to inhaled vancomycin and telavancin. We aimed to develop a once-daily inhaled product with a lung residence half-life in the range of 6 to 24 h. Slower elimination kinetics (half-life up to ca. 120 h) could be acceptable if the dosing frequency was reduced correspondingly, as long as the drug remained active at the site of infection. A longer elimination time, however, may be undesirable in the clinical setting for situations where the treatment needs to be terminated or adjusted according to a patient response (25). In this investigation, vancomycin yielded a favorable lung PK profile, with a half-life calculated to be 23 h (Table 2). In contrast, the vancomycin derivative telavancin and its synthetic intermediate RV40 were sustained in the lung for the duration of the 120 and 168 h experiments, respectively, with negligible exposure in the systemic circulation. In a previous investigation, we showed that the long residence time of RV40 in the lung was met with reduced activity when predosed up to 168 h before challenge in an acute pulmonary MRSA infection in neutropenic rats (26). The hydrolysable amide and ester RV lipoglycopeptides RV62 and RV88 showed apparently optimized clearance; however, measurement of their primary hydrolysis products showed conversion and subsequent pulmonary retention over the course of the 120 h experiments (Fig. S3). This was somewhat surprising based on the chemistries of the by-products, which resemble vancomycin in terms of size, structure, and predictive lipophilicities. Unlike vancomycin, the ester and amide by-products RV82 and RV80 are cationic at physiological pH, so one theory to support the lung retention observation could be that the parent lipoglycopeptides efficiently enter cells in the pulmonary tissues, where they are converted to their charged primary hydrolysis products that then become electrostatically trapped therein. The RV lipoglycopeptide with the most acceptable single dose PK performance was the inverted amide and highly potent RV94, which demonstrated a descending trend in its terminal phase and a half-life calculated to be 108 h. RV94 also demonstrated nominal conversion to its primary hydrolysis product, RV101, and was present at nanogram levels in the systemic circulation, reducing the chance for systemic toxicities in a repeat-dosing treatment protocol.

Based on results combining *in vitro* potency and superior *in vivo* single-dose PK performance, RV94 was highlighted as a promising lead and further investigated to determine its spectrum of activity against an expanded panel of microorganisms. RV94 yielded potent bactericidal activity versus methicillin-susceptible and -resistant S. aureus strains that was superior to vancomycin and telavancin, an approved MRSA therapy of the same class that is regarded as highly potent (Fig. 2 and Table 3). In addition, RV94 displayed activity toward vancomycin-intermediate and -resistant organisms, supporting the claim for additional antibacterial mechanisms of action that align with the lipoglycopeptide class (11). Moreover, and beyond the scope of this report, RV94 also demonstrated potent and superior activity to comparators when tested against the Gram-positive aerobes enterococci and streptococci, as well as the anaerobes *Clostridium*, *Peptostreptococcus*, *Propionibacterium*, *and Eggerthella* (Table 3), which provides a basis for future pursuit of indications beyond pulmonary MRSA infection.

A key objective of this effort was to address protected colonies of MRSA inside cells and in biofilms, which are thought to contribute to clinical relapse after antibiotic treatment (16, 17). Although S. aureus has historically been described as an extracellular pathogen in human respiratory infections, there is a growing body of research suggesting the potential for an intracellular lifestyle for the organism (6, 8, 17, 27–29). This is important, as the intracellular persistence of MRSA can enable host immune evasion and can be a source for relapse after antibiotic therapy (17). A focus of the RV compound design effort was to preserve the cellular penetration capability of lipoglycopeptides while improving upon the potential drawbacks associated with systemic lipoglycopeptide treatment, and, more recently, pulmonary-targeted vancomycin inhalation therapy (4, 5).

The cellular accumulation of RV lipoglycopeptides in THP-1 cells was greater than vancomycin and the lipoglycopeptide telavancin (Fig. 3A), the latter of which has been reported to efficiently accumulate in phagocytes and act on intracellular S. aureus infection therein (29, 30). Of note, the measurement of cell accumulation obtained using this method does not differentiate between compounds that accumulate inside the cell versus those that bind to the membrane, as reported previously. This could be rectified in future studies by amending the assay to accommodate those methods (31). That said, the efficacy of the RV compounds against intracellular MRSA ATCC BAA 1556 (Fig. 3B) suggests that the treatments are able to access and act on bacteria colonized inside cells. Vancomycin, by contrast, had no appreciable effect on intracellular MRSA compared to untreated cultures. Among the lipoglycopeptides tested in this assay, RV40 demonstrated the greatest efficacy, with up to a 2 log_10_ CFU/ml reduction in MRSA titer versus control. More moderate activities were observed from RV94 and telavancin, with MRSA titer reductions in the range of approximately 0.8 log_10_ CFU/ml at the highest dose investigated (64 μg/ml). Notably, this result may be reflected by the limited solubility of RV94 in the testing medium at concentrations greater than 50 μg/ml.

Biofilm-associated bacteria are believed to be another source of clinical relapse after treatment of pulmonary MRSA infection (16, 32). The complex matrix that embeds bacteria makes them notoriously tolerant to conventional antibiotics, including vancomycin, which lack the chemical properties to efficiently penetrate biofilms. Lipoglycopeptides, through their enhanced lipophilicities, are better disruptors of the biofilm matrix, which can aid in subsequently unleashing their potent activities on the protected colonies that thrive in a biofilm state (12). *In vitro* MRSA biofilm activities of RV94 and comparators were first screened in an adapted simple static biofilm in a microtiter plate supplemented with MEM, as previously described (33), and then later in a more biomimetic plasma-supplemented MBEC assay. RV94 demonstrated activity versus biofilm MRSA (ATCC BAA 1556) in both assays that was superior to vancomycin with a 1.6 log_10_ CFU/ml reduction in biofilm MRSA at a dose equal to 2.5 μg/ml in the microtiter plate assay (Fig. 4A) and a 1.4 log_10_ CFU/peg reduction in biofilm MRSA at 16 μg/ml in the MBEC assay. As observed in the *in vitro* intracellular investigation, the activity of RV94 appeared to plateau at the higher doses investigated, which may be an artifact of the testing methodology due to insolubility of RV94 in the testing media at the higher concentrations. RV40, a hydrophobic semisynthetic intermediate in the preparation of telavancin, was the most efficacious compound identified in the *in vitro* biofilm activity screen by both the microtiter plate and MBEC methods, with bactericidal MRSA killing in both assays. Interestingly, vancomycin was effective against biofilm MRSA at high treatment doses in the MEM-supplemented assay (3 log_10_ CFU/ml at >20 μg/ml dose); however, its efficacy diminished to an approximate 1 log_10_ CFU/peg reduction against the same pathogen in a plasma-supplemented MBEC assay (Fig. 4A and B). This demonstrates that in a biofilm model that better represents *in vivo* conditions, the potencies of the lipoglycopeptides are further and preferentially differentiated from vancomycin.

After confirmation of potent *in vitro* activity against MRSA in planktonic and protected colonies, RV94 *in vivo* efficacy was evaluated using an acute pulmonary MRSA infection in neutropenic rats. Vancomycin was chosen as a comparator in these studies due to its optimized PK when administered by inhalation and its relevance as an investigational therapy for pulmonary MRSA in CF, and RV40 was chosen due to its high *in vitro* potency against MRSA in planktonic, intracellular, and biofilm states. Dose targets delivered to the nose of the animals for the RV lipoglycopeptides (ca. 10 to 20 mg/kg) were selected to align with those previously reported to demonstrate antibacterial efficacy from systemically delivered telavancin in a mouse MRSA pneumonia model (34). The selected vancomycin dose was chosen to be significantly greater than lipoglycopeptide target doses (ca. 50 mg/kg) due to its comparatively inferior *in vitro* MRSA activity.

Nebulized inhaled vancomycin, when administered at a dose that was 2.5- to 3.5-fold greater than the lipoglycopeptides, was shown to be ineffective at reducing MRSA lung titer versus vehicle control in this model. Both the inhaled nebulized RV40 and RV94 lipoglycopeptide treatments yielded efficacious reductions in lung MRSA titer compared to inhaled vehicle control (Fig. 5). Of note, from the PK data (Table 2) and consistent with theory (35, 36), the corresponding pulmonary-deposited dose in these experiments is expected to be approximately 10% of the dose delivered to the nose of the animals or 5 mg/kg for vancomycin and 1 mg/kg for the RV lipoglycopeptides. For the RV lipoglycopeptides, this would yield an approximate 250 μg/g pulmonary drug concentration, which is significantly greater than the efficacious doses observed for the RV lipoglycopeptides in the *in vitro* models, being several orders of magnitude greater than the MRSA MIC, approximately 4-fold greater than the efficacious intracellular RV lipoglycopeptide concentrations, and approximately 2.5-fold greater than the efficacious biofilm RV lipoglycopeptide concentrations. Among the RV lipoglycopeptides, RV94 demonstrated moderately superior activity to RV40 that was statistically significant versus vehicle control, with an average reduction of MRSA lung titer equal to 1.0 log_10_ CFU MRSA; however, this result could be influenced by the infection variability observed in this model and corresponding reduced measured MRSA titer in control rats in the RV94 experiment compared to the RV40 experiment.

In future studies, treatment investigations of repeat doses of RV lipoglycopeptides and comparator antibiotic treatments in a weeks-long chronic pulmonary infection model in rodents would be a better predictor of clinical outcome for the intended indication. However, while rodent models of chronic infection have been well characterized for Gram-negative pathogens such as Pseudomonas and nontuberculous mycobacteria (37, 38), development of a reliable chronic MRSA model has been challenging (39), in part due to the steep mortality curve associated with the infection. As such, we and others in this field have resorted to using acute pulmonary infection models to establish initial *in vivo* proof of concept (34, 40–42).

In summary, we have developed and screened a library of next-generation semisynthetic lipoglycopeptides to select for an inhalation therapy with optimized PK and PD for the treatment of persistent pulmonary MRSA infection. Several compounds in the library demonstrated noteworthy *in vitro* activities that were on average 30- to 60-fold more potent than vancomycin. Single-dose *in vivo* PK evaluations of nebulized inhaled RV compounds and comparators led to the selection of RV94, a C10 inverted amide lipoglycopeptide that demonstrated the most favorable pulmonary elimination kinetics. Advanced *in vitro* evaluation of RV94 revealed superior performance compared to vancomycin at killing protected colonies of MRSA residing in biofilms and inside cells. Moreover, RV94 was determined to be effective *in vivo* for the treatment of an acute pulmonary MRSA infection in neutropenic rats, where it yielded a statistically significant reduction in MRSA lung titer versus control that was superior to inhaled vancomycin.

## MATERIALS AND METHODS

### Ethical statement.

All the *in vivo* experiments were carried out according to the guidelines for animal ethics in accordance with the Canadian Council for Animal Care (CCAC) at IPS Therapeutique (protocol DS20190606-05, Sherbrooke, QC, Canada), institutional animal care and use committees (IACUC) at Rutgers University (protocol PROTO999900355; Piscataway, NJ), and Transpharm Preclinical Solutions (protocol TP-27; Jackson, MI).

### Experimental compounds.

RV lipoglycopeptide synthesis typically involved reductive amination between an aldehyde and the vancosamine nitrogen of vancomycin (Scheme S1). Synthetic derivatives were purified by reverse phase preparative high-performance liquid chromatography (HPLC). Compound identity was confirmed using liquid chromatography mass spectrometry (LC-MS). The chemical purity of RV lipoglycopeptide derivatives and telavancin was measured using HPLC-UV and in all cases was >95%. RV40 was synthesized as previously described (15). Telavancin was acquired from Cayman Chemical (Ann Arbor, MI) or AdooQ BioScience (Irvine, CA) and was synthesized as previously described (15). Additional synthetic details and compound characterization data are presented in the supplemental material. Table 1 includes a comprehensive listing of RV lipoglycopeptides prepared as part of this study.

### *In vitro* susceptibility and bactericidal activity.

Clinical and Laboratory Standards Institute (CLSI) guidelines were followed for the determination of minimum inhibitory and minimum bactericidal concentrations using broth microdilution (43–46). In-house RV lipoglycopeptide *in vitro* susceptibility was screened using MRSA ATCC BAA 1556 (USA300, SCC*mec*IV, pvl^+^) and the quality standard MSSA ATCC 29213 (American Type Culture Collection, Manassas, VA). Select compounds were advanced into susceptibility testing against an expanded panel of Gram-positive and Gram-negative organisms using broth microdilution at Micromyx LLC (Kalamazoo, MI) and these organisms are listed in Table S2. The test organisms in the expanded screening panel consisted of clinical isolates from the Micromyx internal repository, as well as reference strains acquired from the ATCC and the Network on Antimicrobial Resistance in S. aureus (NARSA; BEI, Manassas, VA). RV94 was evaluated against vancomycin-resistant S. aureus (VRSA) at JMI Laboratories (North Liberty, IA), and the test organisms were acquired from the NARSA repository (BEI, Manassas, VA).

### *In vitro* cellular accumulation.

THP-1 cells (TIB-202; ATCC, Manassas, VA; 1 × 10^6^ cells/well) were differentiated into macrophages by incubating in 10% fetal bovine serum (FBS; HyClone, Marlborough, MA) supplemented RPMI 1640 medium (HyClone) with 50 ng/ml of phorbol 12-myristate 13-acetate (PMA; Sigma-Aldrich, St. Louis, MO) for 24 h. After a 24 h incubation in fresh medium, cells were treated with RV lipoglycopeptides or comparator drugs at target concentrations in the range of 10 to 75 μg/ml and incubated at 37°C for 24 h. For sample collection, mixtures of cells and medium were transferred into a 1.5-ml microcentrifuge tube followed by centrifugation at 330 × *g* for 8 min at 4°C. The supernatant was collected to measure the extracellular portion. After washing three times using 1 ml of tris-buffered saline (TBS, 1× solution, pH 7.4, Fisher BioReagents, Waltham, MA), the cell pellet was resuspended in TBS to measure the cellular drug concentration. To confirm mass balance, drug concentrations were also measured from the supernatant and an organic solvent mixture (30% acetonitrile in water) that was used to extract residual drug from the empty wells after the cell mixture was removed. The drug from each fraction was then extracted using a customized protein precipitation method with an internal standard for each compound and transferred for analysis by liquid chromatography/tandem mass spectrometry (LC/MS-MS) (Sciex API4500 or API6500+ mass spectrometer, Sciex, Framingham, MA). Cellular accumulation was expressed as μg of drug per the mass of cell protein measured using a Pierce BCA protein assay kit (Thermo Scientific, Waltham, MA).

### *In vitro* intracellular efficacy.

THP-1 cells were cultured in RPMI 1640 medium supplemented with 10% FBS at 37°C in an atmosphere of 5% CO_2_. Unless specified otherwise, this is the medium used throughout the experiment. To create adherent macrophages for the infection and treatment assay, THP-1 cells were first grown to 1 × 10^6^ cells per ml in suspension and then seeded into flat-bottom tissue culture-treated 48-well plates (Corning, Corning, NY) at a density of 3 × 10^5^ cells per well in fresh RPMI medium containing 50 ng/ml PMA for 24 h. At 24 h, medium was replaced with fresh medium for an additional 24 h. Cells were infected with a log-phase culture of MRSA ATCC BAA 1556 at a multiplicity of infection (MOI) of 10 in fresh medium by synchronizing the infection at 150 × *g* for 10 min followed by 1 h of incubation at 37°C to allow intracellular uptake of bacteria. Wells were gently washed twice with phosphate-buffered saline (PBS; Corning) and then incubated with fresh medium containing 25 mg/ml of lysostaphin (Ambi, Lawrence, NY) for 2 h to eliminate residual extracellular bacteria. Wells were washed once with PBS to remove excess lysostaphin, replaced with fresh medium containing 150 nM bafilomycin A1 as previously reported (47) (Alfa Aesar, Haverhill, MA) and test compounds, and then incubated for 24 h. Control wells were incubated with fresh medium containing 150 nM bafilomycin A1 and 2 μg/ml gentamicin, which is a subinhibitory concentration against the intracellular bacteria in this assay but a high enough concentration to control extracellular outgrowth. After 24 h, medium in the wells was removed and replaced with sterile deionized water containing 0.1% Triton X-100 (Sigma-Aldrich, St. Louis, MO) and incubated for 10 min to permeabilize the cells. Complete lysis and homogenization were achieved via mixing wells 20× with a pipette. Lysed wells were serially diluted, and spot plated onto tryptic soy agar containing 5% wt/vol activated charcoal (Sigma-Aldrich) to enumerate surviving intracellular bacteria. Viable counts were reported as log_10_ CFU/ml with a limit of detection equal to 2.0 log_10_ CFU/ml.

### *In vitro* static biofilm efficacy evaluated via plastic microtiter plate method.

MRSA ATCC BAA 1556 was grown in tryptic soy broth (TSB) and overnight in a temperature-controlled shaker at 37°C for 16 h. All antibiotics were dissolved in dimethyl sulfoxide (DMSO) to form stock solutions, except for vancomycin, which was dissolved in sterile water. Vancomycin stock solutions were serially diluted in minimal essential medium (MEM) (1,000 to 0.03 μg/ml). All the other stock solutions were serially diluted in MEM with 0.002% polysorbate 80 and 1% DMSO (80 to 0.08 μg/ml for RV lipoglycopeptides). MRSA ATCC BAA 1556 biofilms were formed in plastic microtiter plates, as described previously (33, 48). Briefly, overnight liquid cultures of MRSA ATCC BAA 1556 were diluted to an optical density at 600 nm (OD_600_) of 0.05 using a plate reader (Synergy Neo, Biotek, Winooski, VT). The cultures were washed in PBS twice and resuspended in MEM. The resulting suspension (100 μl) was added into each well of the 96-well plate and incubated at 37°C. At 1 h postinoculation, the medium in each well was replenished with 90 μl of fresh MEM. At 6 h postinoculation, the medium was removed again and 90 μl of serially diluted antibiotic solutions were added for treatment. The plate was then incubated at 37°C for another 16 h before disruption. To disrupt the biofilm, planktonic cells were removed and 50 μl of 0.1% Triton X-100 in PBS was added, followed by gentle agitation on a rocker for 30 min. The wells were then scraped using a 96-pin microplate replicator, covered with an adhesive sealing film, and vortexed for 2 min. The disrupted biofilms were collected and serially diluted for CFU enumeration. Viable counts were reported as log_10_ CFU/ml with limit of detection equal to 2.6 log_10_ CFU/ml.

### *In vitro* biofilm efficacy evaluated via MBEC system.

The assay was performed with a slight modification of the manufacturer’s instructions of the MBEC Assay System from Innovotech (22). Briefly, a starting inoculum of 7.7 log_10_ CFU/ml was established by overnight culture of a single colony from a TSB plate. Biofilms were developed in TSB with the addition of 1% human plasma (BioIVT, Westbury, NY) at 37°C for 24 h on an orbital shaker (GeneMate MP4). The peg lid was then rinsed twice with sterile PBS to remove sessile bacteria before placing into a challenge plate that contained fresh uninoculated broth with serial dilutions of each antibiotic. All antibiotics were serially diluted in cation-adjusted Mueller-Hinton broth (MHIIB). The challenge plate was then incubated for another 24 h at 37°C and the peg lid was rinsed twice with PBS and then placed into a recovery plate containing MHIIB plus 0.1% Triton X-100. Adherent biofilms were disrupted by sonication in a recovery plate for 30 min. The sonication cycle was repeated once after placing the peg lid into another fresh recovery plate containing MHIIB plus 0.1% Triton X-100. Samples from both recovery plates were combined and mixed at least five times. The resulting sample mixture was then serially diluted, followed by spot plating onto tryptic soy agar containing 5% wt/vol activated charcoal, and the number of CFU per peg, as well as the log reduction of the antimicrobial at each concentration tested, was determined. The limit of detection was 1.1 log_10_ CFU/peg.

### *In vivo* PK.

Male Sprague Dawley rats (Charles River Laboratories, QC, Canada) weighing between 245 and 325 g were used for the PK evaluation of single-dose nebulized nose-only inhalation administrations of RV lipoglycopeptides and comparators using a 12-port nose-only inhalation tower (CH Technologies, Westwood, NJ). Rats were acclimated in the study facility for approximately 1 week prior to study start. Ten or eleven rats per cohort were loaded into the chamber at the time of dosing. RV lipoglycopeptides and comparators were aerosolized using an Aerogen Pro nebulizer (Aerogen, Galway, Ireland), which generates droplet sizes (median mass aerodynamic diameter) ranging from 2.5 to 4 μm at output rates ranging from 0.2 to 0.4 ml/min. An additional airflow set to 6 liters/min was admixed with the nebulizer output to further direct the test articles to the animals. The lipoglycopeptides telavancin, RV40, RV62, RV88, and RV94 were formulated in bicine buffer (34 mM) adjusted to pH 9.0 to 9.4 with sodium hydroxide, and vancomycin was formulated in sterile water. Single-dose administrations of formulated drugs were dosed to the animals at drug concentrations ranging from 5 to 30 mg/ml to approximately delivered dose targets from approximately 1 to 10 mg/kg. The remaining port on the chamber was fit with an aerosol sampling filter for dose determination over a 5 min sampling period. The average dose delivered to the nose of each animal was calculated using an algorithm that was previously described (49). Sample sizes were *n* = 2 to 4 animals per time point for *n* = 4 to 7 time points dosed in one or two cohorts. Animals were euthanized for collection of whole lungs and plasma at time points ranging from immediate postdose (IPD; approximately 30 min after dosing for nebulization) to 168 h postdose (7 days). Drugs were extracted from tissues using customized protein precipitation methods with an appropriate internal standard for each compound and transferred for analysis by LC-MS/MS (Sciex API4500 or API6500+ mass spectrometer [Sciex, Framingham, MA]).

### *In vivo* efficacy.

Male Sprague Dawley rats (Envigo, Indianapolis, IN) weighing between 225 and 250 g were used in these studies. Rats were housed and acclimated to the study facility as described in the PK methods section. Rats were rendered neutropenic through a series of intraperitoneal cyclophosphamide (Sigma-Aldrich) injections on day −4 (150 mg/kg) and day −1 (100 mg/kg) relative to the challenge. On day 0 at 0 h, rats were challenged with MRSA ATCC BAA 1556 via intranasal instillation at a target titer of 8.0 to 8.3 log_10_ CFU using optical density and back-counting inoculum CFU. To prepare the inoculum, a frozen aliquot of the organism stored at −80°C was thawed, transferred into TSB, and incubated at 37°C while shaking for 16 h. Cultures were then centrifuged at 2,000 × *g* for 5 min, and the resulting pellet was resuspended in PBS and equilibrated. For the challenge procedure, rats were first anesthetized through use of 2 to 5% isoflurane in 100% oxygen, then restrained with a scruff grip and a total of 200 μl of inoculum was quickly introduced to one nare. The rat was held in a vertical position until the challenge was fully inhaled. Rats were monitored following challenge to ensure that normal respiration was achieved and then returned to their cage. At 24 and 32 h postchallenge, rats (*n* = 10 per cohort for RV94, vancomycin, and their respective vehicle controls or *n* = 8 for RV40 and its vehicle controls) were loaded into the inhalation dosing tower for nebulized drug or vehicle control treatments by nose-only inhalation using procedures described in the PK study methods section. Body weight target doses ranged from 10 to 50 mg/kg of drug delivered to the nose of each rat. Animals were euthanized via carbon dioxide overexposure at 48 h postinfection for enumeration of lung MRSA titer. At necropsy, whole lungs were aseptically removed and lung weights were recorded. Lungs were homogenized using stainless steel beads in sterile water with a Bead Ruptor Elite homogenizer (Omni International, Kennesaw, GA). Mixed, homogenized samples were serially diluted and plated on Vogel Johnson S. aureus selective media (Sigma-Aldrich) using sterile disposable cell spreaders. Plates were then incubated at 37°C for 24 to 48 h before colonies were enumerated.

### Statistical analysis.

GraphPad Prism (La Jolla, CA) was used for statistical analyses and data plotting. Analysis of the lung and plasma PK was performed using the open-source software add-in PKSolver 2.0 for Microsoft Excel. The data were applied to a noncompartmental analysis after extravascular dosing input and fit to a linear up/log down model. Statistical analysis of *in vitro* intracellular and biofilm activity was based on a two-way ANOVA with a Bonferroni post test. *In vivo* statistical analysis was based on the Kolmogorov-Smirnov test.
