# Supplementary material for: Development and Preclinical Evaluation of New Inhaled Lipoglycopeptides for the Treatment of Persistent Pulmonary Methicillin-Resistant Staphylococcus aureus Infections
Source: Antimicrob Agents Chemother. 2021 Jun 17;65(7):e00316-21. doi: 10.1128/AAC.00316-21 (PMC8373216; doi:10.1128/AAC.00316-21)
Supplement: Supplemental file 1 — Supplemental material. Download AAC.00316-21-s0001.pdf, PDF file, 3.3 MB [file aac.00316-21-s0001.pdf]

Supplemental Materials: Development and Preclinical Evaluation of New Inhaled Lipoglycopeptides for the Treatment of Persistent Pulmonary MRSA Infections

Authors: Adam J. Plaunt, Sasha J. Rose, Jeong Yeon Kang, Kuan-Ju Chen, Daniel LaSala, Ryan P. Heckler, Arielle Dorfman, Barrett T. Smith, Donald Chun, Veronica Viramontes, Antonio Macaluso, Zhili Li, Yuchen Zhou, Lilly Mark, Jessica Basso, Franziska G. Leifer, Michel R. Corboz, Richard W. Chapman, David Cipolla, Walter R. Perkins, Vladimir S. Malinin, and Donna M. Konicek#

Insmed Incorporated, Bridgewater, New Jersey, USA

# Address correspondence to Donna M. Konicek, donna.konicek@insmed.com

CONTENTS

METHODS 3
ENZYME HYDROLYSIS 6
PLASMA STABILITY 8
RV LIPOGLYCOPEPTIDE AND COMPARATOR PK 9
EXPANDED PANEL MIC 10
BIOFILM GROWTH MODEL 12
IN VITRO CELL CYTOTOXICITY 13
EXPERIMENTAL COMPOUNDS 14
SYNTHESIS PROCEDURES 15
- REPRESENTATIVE PREPARATION OF A CONVENTIONAL ESTER – RV65 19
- REPRESENTATIVE PREPARATION OF AN INVERTED ESTER – RV55 22
- REPRESENTATIVE PREPARATION OF A CONVENTIONAL AMIDE – RV62 25
- REPRESENTATIVE PREPARATION OF AN INVERTED AMIDE – RV94 29
- PREPARATION OF RV LIPOGLYCOPEPTIDE METABOLITES 33
- PREPARATION OF RV LIPOGLYCOPEPTIDE COMPARATORS 39

CHEMICAL CHARACTERIZATION 42
- NMR SPECTRA 42
- MS DATA 56

HPLC & LCMS CHROMATOGRAMS.. 60

METHODS

Enzyme hydrolysis. In general, RV lipoglycopeptides were formulated at pH 8.0 either as an aqueous solution or a suspension with hydroxy-beta-cyclodextrin (HPCD) as the primary excipient. To solutions of formulated RV lipoglycopeptide derivatives were added the target enzyme as a solution in PBS. The solutions were placed in an incubated shaker set to 37 °C and aliquots were removed at pre-specified time points (typically 0, 15, 30, 45, 60, 75, 90, and 120 minutes). Enzyme activity was halted by dilution in acidic media. The aliquots were then assessed by HPLC to monitor conversion from the parent lipoglycopeptide to the primary metabolite. Results are presented graphically as the percent degradation vs. time where percent degradation is defined as follows:

$$Percent\ Degradation = \frac{[Metabolite]}{[LGPC] + [Metabolite]}\ x\ 100$$

Enzymes studied include:

- Esterase from Porcine Liver (Sigma Aldrich, E3019)
- Carboxypeptidase A from bovine pancreas (Sigma Aldrich, C9268)
- Protease from *Aspergillus oryzae* (Sigma Aldrich, P6110)
- Papain from papaya latex (Sigma Aldrich, P4762)
- Protease from *Bacillus lichenformis* (Sigma Aldrich, P5380)
- Protease from Bovine Pancreas (Sigma Aldrich, P4630)

MIC Testing. Select compounds were advanced into susceptibility testing against an expanded panel of gram-positive and gram-negative organisms using broth microdilution at Micromyx LLC (Kalamazoo, MI) and these organisms are listed in Table S2. The test organisms in the expanded screening panel consisted of clinical isolates from the Micromyx internal repository, as well as reference strains acquired from the ATCC and the Network on Antimicrobial Resistance in S. aureus (NARSA; BEI, Manassas, VA). RV94 was

evaluated against Vancomycin-Resistant S. Aureus (VRSA) at JMI Laboratories (North Liberty, IA) and the test organisms were acquired from the NARSA repository (BEI, Manassas, VA).

Plasma stability. Stock solutions of RV lipoglycopeptide derivatives RV62 and RV65 were prepared in 100% DMSO. Stock solutions were diluted using rat plasma to contain less than 1% organic solvent with a final drug concentration of 50 µg/mL. Samples were briefly vortexed and then incubated in a shaker set to 37 °C and 300 rpm. Aliquots were removed at specified time points and store at – 80 °C until extraction (10% trichloroacetic acid in acetonitrile) and analysis (LC/MS).

Crystal violet staining of biofilm. Biofilms were grown on the pegs of the lid under shaking conditions for 24 h based on the protocol above at 37 °C with various culture conditions (TSB only, TSB with 1% glucose, TSB with either 1%, 2.5%, or 10% human plasma, TSB with either 1%, 10%, or 50% human serum, TSB with 1% glucose plus 10% human plasma, and TSB with 1% glucose plus 50% human serum; Both plasma and serum were obtained from BioIVT). The lid was removed, and the pegs were gently washed twice with 200 µL of PBS to remove nonadherent cells. Lids with biofilms were air-dried for 5 min. Adherent biofilms on the pegs were stained with 200 µL 0.1% (wt/vol) crystal violet in water for injection (WFI) for 15 min at room temperature. The pegs were then washed twice with 200 µL of PBS to remove excess stain. Lids were air-dried for 10 min before de-staining with acetic acid. The pegs were de-stained with 200 µL of 33% acetic acid to solubilize the dye for 15 min. Quantitative assessment of biofilm formation was obtained by measuring the absorbance at 600 nm using a plate reader.

***In vitro* cell cytotoxicity**

THP-1 cells were maintained at 37°C with 5% CO_2 in growth media (RPMI-1640 + 10% Fetal Bovine Serum) and passaged when cell density had reached roughly 1 x 10^6 cells/mL. PMA-differentiated cells for experiments were prepared by seeding 40,000 cells per well in clear-bottom black plastic 96-well plates at a final concentration of 50 ng/mL of PMA in a final volume of 200 uL. Plates were incubated at 37°C for 24 h, after which media was changed twice with growth media (no PMA). Cells were given an additional

24 h to recover from PMA treatment after which compound challenge was applied in a total volume of 100 µL of media plus compound. After 24 h of compound challenge at 37°C, cell integrity and metabolism were evaluated using the CellTox Green (Promega G8746) and MTT (Promega G4000) assays, respectively. For CellTox Green assay 100 µL of 2x CellTox Green Reagent was added directly to media in each well and plates were shaken briefly (1 min) to ensure reagent homogeneity. Plates were incubated for 15 min at room temperature, shielded from light, after which fluorescence was measured using a plate reader (Ex500/Em532). Membrane integrity was calculated as percent of lysis control from wells that had received 4 µL of Lysis Solution 30 min prior to addition of CellTox Green Reagent, representing complete loss of membrane integrity. For MTT assay 15 µL of Dye Solution was added directly to media in each well and plates were shaken briefly, then returned to the 37°C incubator for 2 h. 100 uL of Solubilization Solution/Stop Mix was then added to each well and the plate was incubated for an additional hour at 37°C. Plates were then mixed on an orbital shaker at 700 rpm for 2 min and absorbance at 570 nm was measured, with a reference wavelength of 650 nm. Results were calculated as percent of no drug control based on OD readings from wells which did not receive drug challenge.

ENZYME HYDROLYSIS

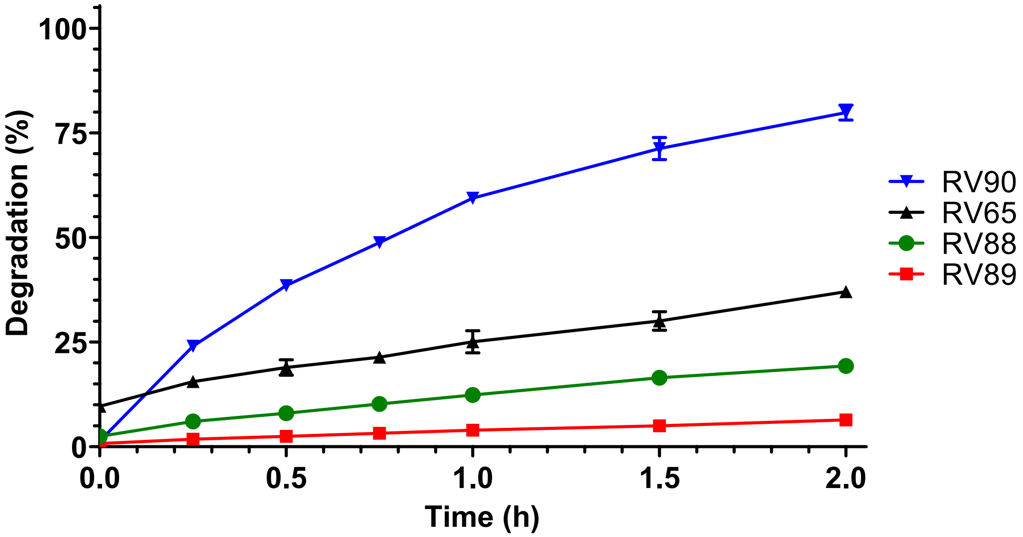

Figure S1: Esterase mediated hydrolysis of conventional ester RV Lipoglycopeptides RV90 (blue triangle, Octyl), RV65 (black triangle, Dodecyl), RV88 (green circle, Tetradecyl), and RV89 (red squares, Hexadecyl) was measured as a function of time in the presence of porcine liver esterase; parenthesis indicate total number of atoms in the cleavable linker. Samples were analyzed by HPLC (UV, 281 nm) to determine the relative peak area for both the parent RV lipoglycopeptide and the metabolite (RV80) for each compound tested.

Table S1: Enzyme hydrolysis of RV62 and RV94 was measured as a function of time in PBS (pH 8) at 37 °C. Control samples in the absence of enzyme were included as positive controls. Samples were analyzed by HPLC detecting the parent RV derivative (RV62 or RV94).

RV Derivative	Enzyme	[Enzyme], U/mL	Percent RV derivative remaining in HPLC chromatograph, %				
			0 h	0.5 h	1 h	2 h	24 h
RV62	NA	0	97.14	97.14	97.38	97.07	NA
RV62	Carboxypeptidase A from bovine pancreas	2	96.4	95.95	96.06	95.69	NA
RV62	Protease from Aspergillus oryzae	2	96.73	96.17	96.63	95.76	NA
RV94	NA	0	99.74	NA	99.54	NA	98.89
RV94	Protease from Papain	5	99.46	NA	99.39	NA	98.31
RV94	Protease from Bacillus Lichenformis	5	99.76	NA	99.47	NA	99.43
RV94	Protease from Bovine Pancreas	5	99.74	NA	99.36	NA	97.91

PLASMA STABILITY

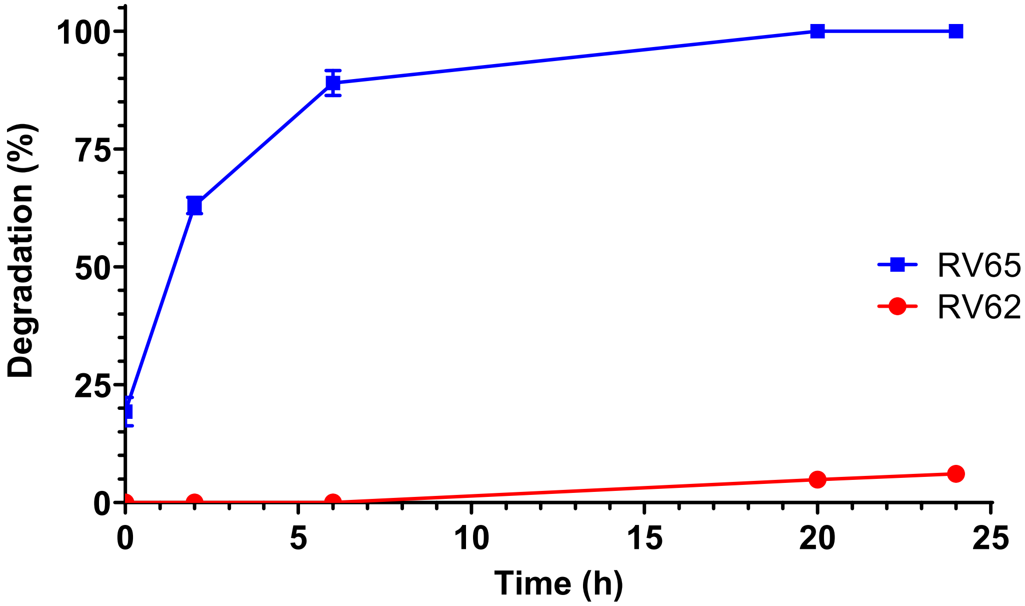

Figure S2: Plasma stability of RV62 (red circles) and RV65 (blue squares) was measured as a function of time in rat plasma at 37 °C. Samples were analyzed for both the parent RV lipoglycopeptide (RV62 or RV65) and the primary metabolite (RV82 or RV80, respectively) using LC/MS.

RV LIPOGLYCOPEPTIDE AND COMPARATOR PK

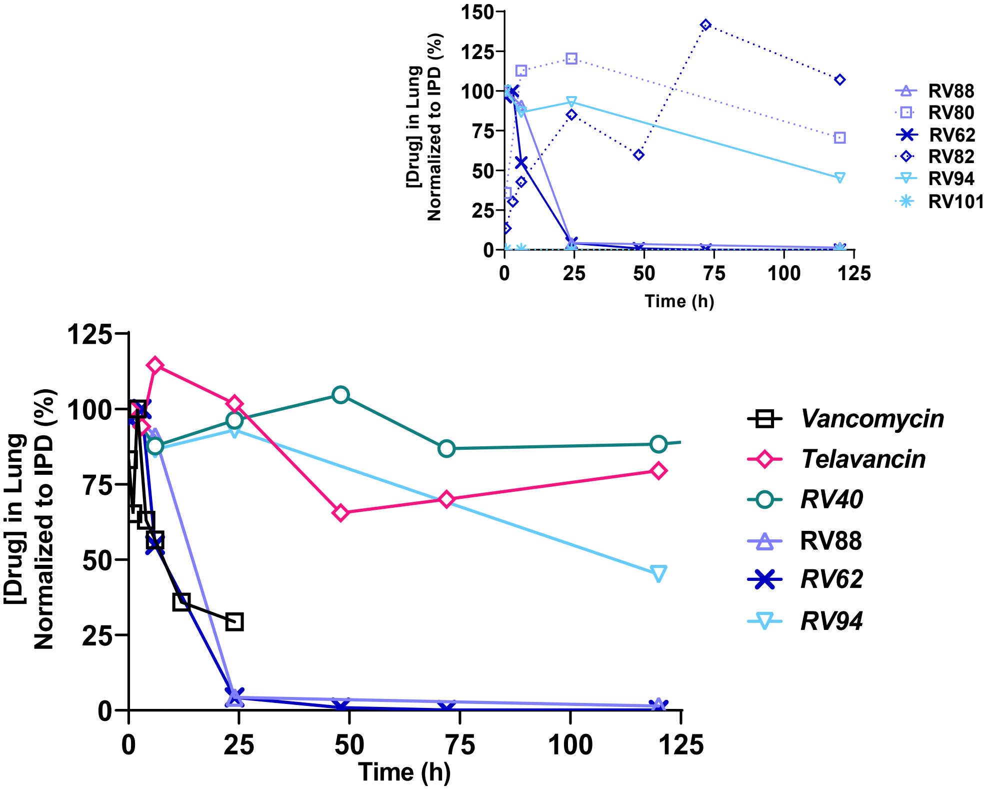

Figure S3. Single dose lung PK of inhaled nebulized RV lipoglycopeptides and comparators administered by nose-only inhalation to healthy rats. Data are the average lung level of n = 2-4 animals per timepoint and are normalized to the concentration of drug in the lung at immediate post-dose (IPD; assumed to be 0.5 h). Doses and pharmacokinetic parameters are listed in Table 3. The RV40 experiment was conducted for 168 h (7 days) and showed a constant level of lung drug concentration. The vancomycin experiment was conducted for 24 h (1 day) and all other experiments were 120 h (5 days). Inset: Ester (RV88) and Amide (RV62)-linked RV lipoglycopeptides were rapidly hydrolyzed to their primary byproducts (RV80 and RV82, respectively) indicated by the dashed lines that accumulated in the lung over the course of the experiment, whereas RV94, an inverted amide remained stable (minimal observed formation of RV101) and was eliminated from the lung more efficiently than telavancin and RV40.

EXPANDED PANEL MIC

Table S2. RV lipoglycopeptide and comparator antibiotic spectrum of activity. ATCC = American Type Culture Collection, MMX = Micromyx Collection, NRS = Network on Antimicrobial Resistance in *S. aureus,* NCTC = National Collection of Type Cultures. NT = not tested.

Organism	Isolate No.	Type	MIC (µg/mL)			
			RV40	Telavancin	RV94	Vancomycin
Gram-Positive Organisms						
S. aureus	ATCC 29213	MSSA	0.008	0.06	0.015	1
S. aureus	ATCC 13709	MSSA	0.015	0.12	0.03	1
S. aureus	MMX 7907	MSSA	0.008	0.06	0.03	0.5
S. aureus	MMX 7908	MSSA	0.015	0.12	0.015	0.5
S. aureus	NRS123	MRSA; USA400	0.008	0.06	0.015	0.5
S. aureus	NRS 384; ATCC BAA-1756	MRSA; USA300	0.008	0.06	0.015	0.5
S. aureus	MMX 3982	MRSA; USA300	0.008	0.06	0.015	0.5
S. aureus	ATCC BAA-1556	MRSA; USA300	0.008	0.06	0.03	0.5
S. aureus	NRS725	MRSA; USA300	0.008	0.06	0.015	0.5
S. aureus	ATCC 43300	MRSA	0.008	0.06	0.015	1
S. aureus	MMX 7899	MRSA	0.008	0.06	0.015	0.5
S. aureus	MMX 7900	MRSA	0.008	0.06	0.015	0.5
S. aureus	MMX 7901	MRSA	0.008	0.03	0.015	0.5
S. aureus	MMX 7902	MRSA	0.015	0.06	0.03	0.5
S. aureus	MMX 5715	MRSA	0.015	0.06	NT	NT
S. aureus	MMX 7903	MRSA	0.008	0.06	0.015	0.5
S. aureus	NRS23	hVISA	0.03	0.25	0.12	2
S. aureus	NRS2	hVISA;Mu3	0.015	0.12	0.03	1
S. aureus	NRS1	VISA; Mu50	0.06	0.5	0.25	4
S. aureus	NRS52	VISA	0.03	0.25	0.06	1
S. aureus	NRS22	VISA	0.06	0.5	0.12	4
S. aureus	NRS4	VISA	0.06	0.25	0.06	4
S. aureus	NRS13	VISA	0.03	0.12	0.12	2
S. epidermidis	ATCC 49134	MSSE	0.015	0.12	0.015	0.5
S. epidermidis	MMX 762	MRSE	0.008	0.12	0.03	1
S. epidermidis	MMX 5145	MRSE	0.004	0.03	0.015	1
S. lugdunensis	MMX 8724	NA	0.004	0.06	0.015	0.25
S. haemolyticus	ATCC 29970	NA	0.015	0.06	0.06	1
S. hominis	ATCC 27844	NA	0.015	0.06	0.03	0.5
E. faecalis	ATCC 29212	VSE	0.015	0.12	0.03	2
E. faecalis	MMX 4176	VSE	0.03	0.25	0.03	1
E. faecalis	MMX 1086	VanA VRE	0.5	1	0.03	128
E. faecium	MMX 4204	VSE	0.004	0.06	0.015	0.5
E. faecium	MMX 851	VanA VRE	1	2	2	128
E. faecium	MMX 173	VanB VRE	0.008	0.06	0.03	64
S. pneumoniae	ATCC 49619	PISP	0.004	0.03	0.008	1
S. pneumoniae	MMX 747	PISP	0.004	0.015	0.004	0.12
S. pneumoniae	MMX 432	PRSP	≤0.008	0.03	0.008	0.25
S. pyogenes	ATCC 19615	NA	0.06	0.06	0.015	0.25
S. pyogenes	MMX 8778	NA	0.015	0.06	0.008	0.25
S. pyogenes	MMX 946	erm^{R}	0.008	0.06	0.008	0.25
S. agalactiae	ATCC 13813	NA	0.008	0.06	0.03	0.25
S. agalactiae	MMX 4088	NA	0.008	0.06	0.008	0.25
S. agalactiae	MMX 4115	erm^{R}	0.008	0.06	0.004	0.25
S. dysgalactiae	MMX 5121	NA	0.008	0.12	0.008	0.25
S. dysgalactiae	MMX 5123	NA	0.12	0.25	0.03	0.25
S. dysgalactiae	MMX 5124	NA	0.015	0.03	0.008	0.25

S. anginosus (AGS)	ATCC 33397	NA	0.015	0.06	0.015	0.5
S. constellatus (AGS)	MMX 5677	NA	0.008	0.03	0.008	0.25
S. mitis (MGS)	ATCC 49456	NA	0.008	0.06	0.008	0.25
S. mitis (MGS)	MMX 5798	NA	0.03	0.03	0.008	0.25
S. oralis (MGS)	MMX 5821	NA	0.015	0.06	0.015	0.5
C. difficile	ATTC 700057	toxAB-	0.06	0.12	0.015	0.25
C. difficile	ATCC BAA-1805	ribo 027	0.06	0.12	0.015	0.5
C. difficile	ATCC BAA-1870	NAP1;ribo 027	0.12	0.12	0.06	1
C. perfringens	ATCC 13124	NA	0.015	0.015	0.008	0.25
P. micros	MMX 3546	NA	0.06	0.12	NG	NG
P. anaerobius	ATCC 27337	NA	0.008	0.03	0.008	0.12
P. acnes	ATCC 6919	NA	0.008	0.03	0.008	0.25
P. acnes	ATCC 11827	NA	0.008	0.015	0.008	0.12
E. lenta	ATCC 43055	NA	NT	NT	0.008	0.5
Gram-Negative Organisms						
E. coli	ATCC 25922	non-ESBL	>64	NT	NT	>64
E. coli	MMX 5684	ESBL (TEM-10)	>64	NT	NT	>64
K. pneumoniae	MMX 2542	-	>64	NT	NT	>64
K. pneumoniae	MMX 4679	KPC-2	>64	NT	NT	>64
P. mirabilis	ATCC 43071	-	>64	NT	NT	>64
P. vulgaris	MMX 9373	-	>64	NT	NT	>64
E. cloacae	ATCC 49141	-	>64	NT	NT	>64
E. cloacae	ATCC BAA-1143	AmpC	>64	NT	NT	>64
C. freundii	MMX 6602	-	>64	NT	NT	>64
S. marcescens	ATCC 43862	-	>64	NT	NT	>64
P. aeruginosa	ATCC 27853	-	>64	NT	NT	>64
P. aeruginosa	MMX 2562	MDR	>64	NT	NT	>64
B. cepacia	MMX 9040	-	>64	NT	NT	>64
A. baumannii	NCTC 13304	OXA-27	>64	NT	NT	>64
A. baumannii	MMX 3372	MDR	64	NT	NT	>64
A. baumannii	MMX 6974	colistinR	32	NT	NT	>64
H. influenzae	ATCC 49247	BL-	64	NT	NT	>64
H. influenzae	MMX 7988	BL+	32	NT	NT	64
H. parainfluenzae	MMX 2529	-	16	NT	NT	64
M. catarrhalis	ATCC 8193	-	32	NT	NT	64
M. catarrhalis	ATCC 8176	-	1	NT	NT	32
M. catarrhalis	MMX 3782	-	1	NT	NT	16

BIOFILM GROWTH MODEL

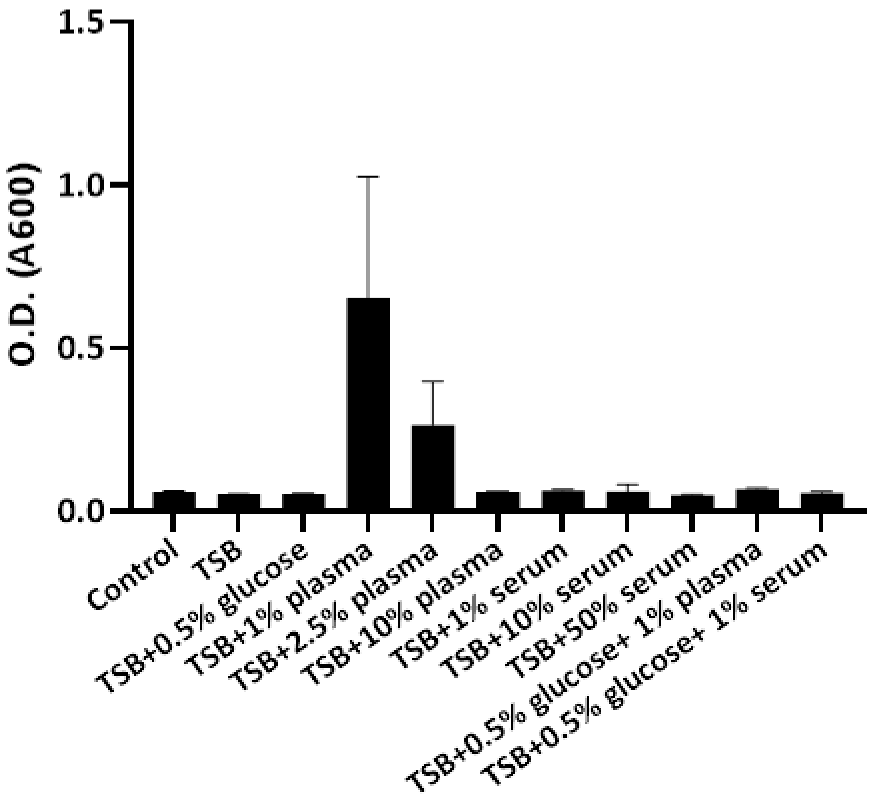

Figure S4. Investigating conditions to enhance biomass on the biofilm growth supports (pegs) for the MBEC assay from a MRSA ATCC BAA-1556 (USA300) in vitro biofilm using crystal violet staining. TSB = tryptic soy broth. The condition that generated the greatest degree of biomass in this experiment (TSB + 1% plasma) was used in the MBEC assay system that yield data in Figure 4B.

***IN VITRO* CELL CYTOTOXICITY**

A)

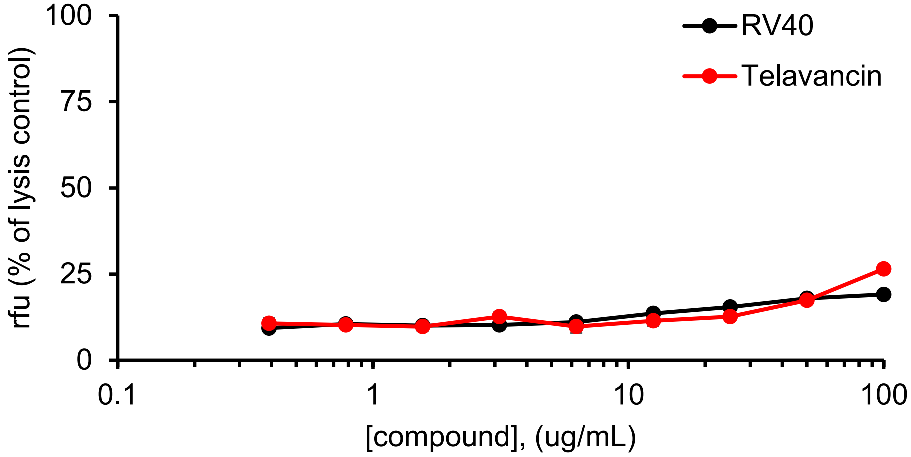

B)

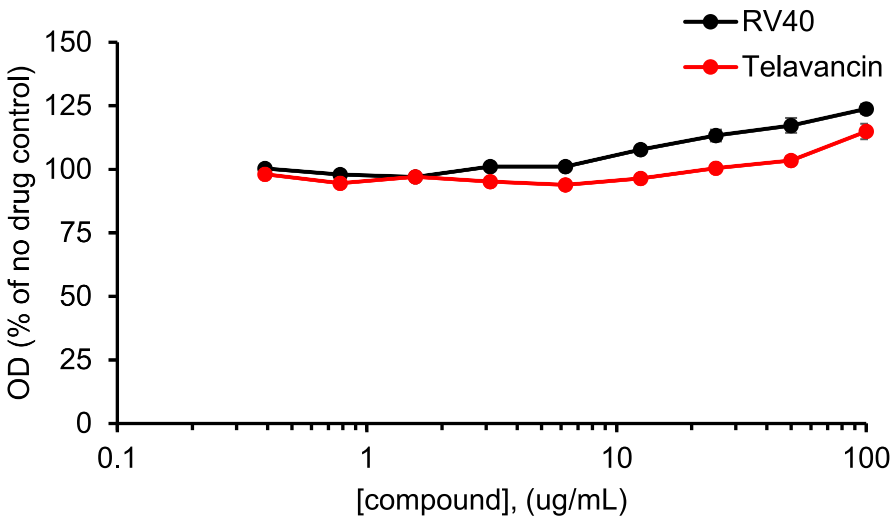

Figure S5. The *in vitro* cell cytoxicity of RV40 and telavancin was tested in THP-1 cells using the A) CellTox Green and B) MTT cytotoxicity assays. n = 4 measurements; error is SEM.

EXPERIMENTAL COMPOUNDS

Chemicals and reagents were obtained from Sigma-Aldrich (St. Louis, M), Fisher Scientific (Waltham, MA), or Afla Aesar (Haverhill, MA). Vancomycin HCl was sourced from Chemwerth Incorporated (Woodbridge, CT). Aldehydes used in reductive amination reactions with vancomycin were typically prepared in-house via oxidation of an alcohol precursor, though in some instances aldehydes were obtained from commercial vendors including Astatech Inc (Bristol, PA), Manchester Organics Ltd (Runcorn, UK), and Shanghai Balmxy Pharmaceutical Co. (Songjiang District, Shanghai, P.R. China); all materials were used as received. Preparative and flash chromatography was performed using a Waters Prep-150 chromatography system (Milford, MA). Flash column chromatography supplies were purchased from Biotage (Charlotte, NC). Preparative HPLC columns were purchased from Phenomenex (Torrance, CA). ^{1}H nuclear magnetic resonance (NMR) spectra were obtained using either a Bruker Advance III HD 500 instrument operating at 500 MHz or a Magritek Spinsolve 60 instrument operating at 60 MHz. ^{13}C nuclear magnetic resonance (NMR) spectra were obtained using a Bruker Avance III HD 500 instrument operating at 126 MHz. Raw NMR data were analyzed using Mestrelab Research Chemistry Software Solutions. Chemical shifts were reported in units of parts per million (ppm; δ) relative to either tetramethylsilane (TMS) as the internal standard or residual solvent proton. Coupling constant values are reported in hertz. HPLC analysis was performed using a Water H-Class UPLC system equipped with PDA (Waters) and CAD (Thermo Fisher) detectors using a Phenomenex Luna 3 μM C18(2) 100Å, 150 x 4.6 mm column using gradients of water and acetonitrile, each containing 0.1% (v/v) of TFA. The chemical purity of RV lipoglycopeptide derivatives and telavancin was measured using HPLC-UV and in all cases was > 95%. Telavancin and RV40 were synthesized as previously described (1).

SYNTHESIS PROCEDURES

Generic Reductive Amination Procedure

To a reactor vessel containing a stirred solution of DMF (50 mL) and DIPEA (4.0 mmol) at 65 °C was added vancomycin HCl (2.9 g, 2.0 mmol), and the mixture was stirred for 10 minutes. The mixture was then cooled to 30 °C at which point the appropriate aldehyde was added (2.8 mmol). The resulting solution was stirred overnight at which point MeOH (25 mL) and TFA (8 mmol) were added. After stirring for 2 hours, Borane tert-butylamine complex (2.0 mmol) was added portion-wise. After stirring for an additional 2 hours the reaction mixture was then purified using reverse phase C18 column chromatography (Phenomenex Luna 10 μM PREP C18(2) 250 x 21.2 mm column) using gradients of water and acetonitrile, each containing 0.1% (v/v) of TFA. In some instances, lactic acid was used instead of TFA to prepare lactic acid salt former of RV lipoglycopeptides. Fractions were evaluated using HPLC and then pertinent fractions containing purified RV lipoglycopeptide were pooled together for the isolation of the product via lyophilization. In general, the target RV lipoglycopeptides were obtained as white solids in modest yield (typically 30-50%) and high purity (>97% by HPLC-UV).

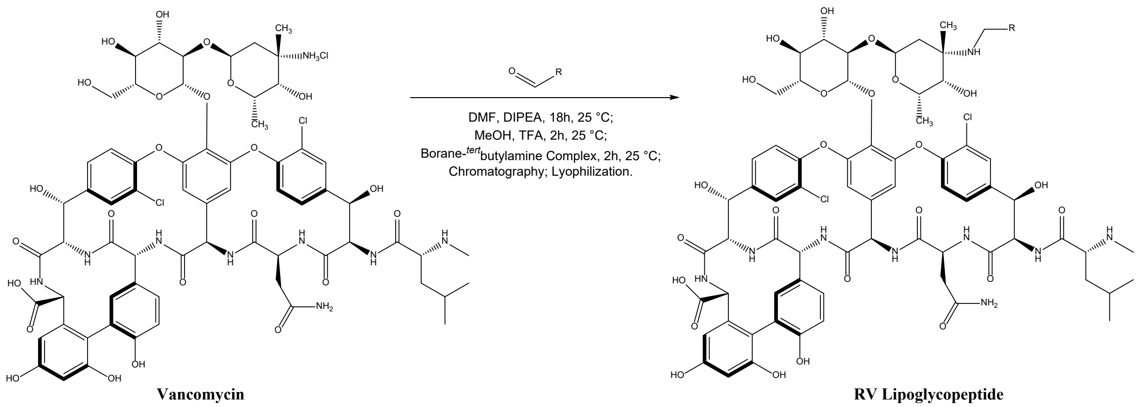

Scheme S1: Generic synthesis scheme depicting a reductive amination reaction to prepare RV lipoglycopeptide derivatives of vancomycin.

Generic aldehyde preparation

To a reactor equipped with a stir bar was added an alcohol reagent, containing an ester or amide bond and a suitable organic solvent (typically DCM or THF). The reaction mixture was stirred for approximately 5 minutes to fully dissolve the starting material at which point sodium bicarbonate and Dess-Martin periodinane were added to the reaction mixture. The reaction mixture stirred for 2 hours, at which point TLC analysis was used to assess progress. In the instance that a large amount of unreacted starting material was present, an additional aliquot of Dess-Martin periodinane was added to the reaction mixture and progress was re-assessed, after an additional 2 hours of stirring. Once complete, the reaction mixture was then treated with DCM and a solution of 10% sodium thiosulfate saturated with $NaHCO_3$ for 90 minutes. The reaction mixture was then extracted with the sodium thiosulfate solutions (3 x 100 mL) and brine (2 x 100 mL), while retaining the organic layer. The organic layer was dried over Na_2SO_4, filtered, and solvent was removed under reduced pressure, to yield the target aldehyde. The final material was typically used without further purification.

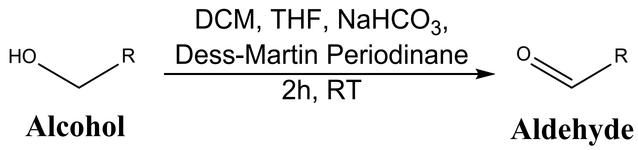

Scheme S2: Generic synthesis scheme depicting an oxidation reaction to prepare an aldehyde

Generic alcohol preparation – Conventional Ester

To a reactor vessel was added 2-iodoethanol, a suitable organic solvent, typically N,N-Dimethylformamide, and an appropriate carboxylic acid. The reaction mixture was then placed in an incubated shaker set at 40 °C and ~125 rpm where it was left to shake overnight. Solvent was removed under reduced pressure and the residue was subjected to liquid-liquid extraction using H_2O (40 mL) and hexanes (3 x 75 mL). Organic layers were combined, and the solvent was removed under reduced pressure. The crude material was purified via silica gel flash column chromatography using a gradient method with hexanes and ethyl acetate

as the mobile phases. Fractions of interest were combined, and the solvent was removed under reduced pressure to produce the target compound, typically as a thick oil.

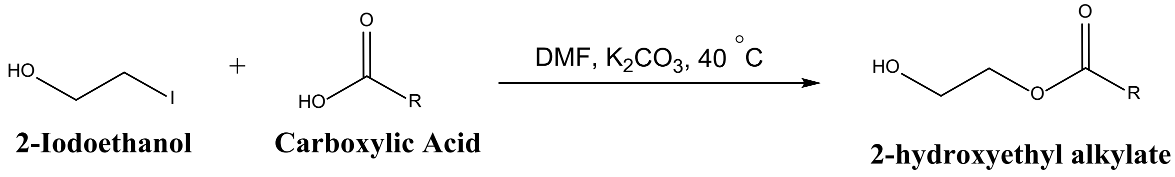

Scheme S3: Generic synthesis scheme depicting preparation of 2-hydroxyethyl alkylates

Generic ester preparation – Inverted Ester

To a vial were added: a suitable organic solvent, such as N,N-Dimethylformamide, an appropriate hydroxyl acid, such as glycolic acid, and an alkyl halide, such as 1-Iododecane. The reaction mixture was then placed in an incubated shaker set at 40 °C and ~125 rpm where it was left to shake overnight. Solvent was removed under reduced pressure and the residue was subjected to liquid-liquid extraction using H_2O (40 mL) and hexanes (3 x 75 ml). Organic layers were combined, and the solvent was removed under reduced pressure. The crude material was purified via silica gel flash column chromatography using a gradient method with hexanes and ethyl acetate as the mobile phases. Fractions of interest were combined, and the solvent was removed under reduced pressure to produce the target compound, typically as a thick oil.

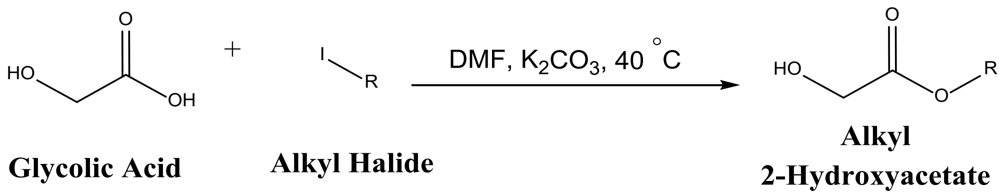

Scheme S4: Generic synthesis scheme depicting preparation of alkyl 2-hydroxyacetates

Generic alcohol preparation – Conventional Amide

To a reactor vessel were added ethanolamine and a suitable organic solvent, typically THF or DCM. Temperature was adjusted to be 0 °C and stirring was initiated. Once the temperature stabilized, triethylamine was added in a single aliquot. Separately, a solution of an appropriate acid chloride and a suitable organic solvent, such as THF or DCM, were prepared and loaded into a dosing apparatus. The

acid chloride solution was added drop wise over the course of few hours, while stirring at 0 °C. The reaction mixture was warmed to 25 °C over a two-hour period and the reaction mixture was stirred for approximately 18 hours, at which point stirring was stopped. The reaction mixture was filtered to remove a white precipitate that had formed. Solvent was removed under reduced pressure to yield a thick, colorless oil. The crude material was dissolved in EtOAc, and washed with 0.1M HCl, saturated $NaHCO_3$, and brine. The organic layer was dried over Na_2SO_4, filtered, and evaporated to dryness, to yield crude product, typically as a white solid. The crude material was purified using prep-HPLC with a CN column and an isocratic method with 10% isopropyl alcohol as the mobile phase. Pure fractions were combined, and solvent was removed to yield the target compound, typically as a white solid.

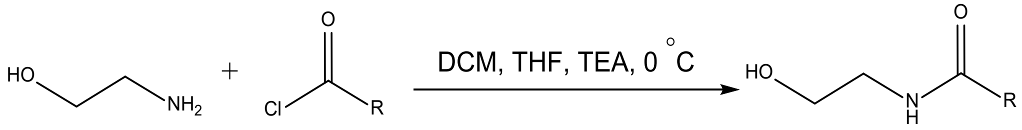

Ethanolamine **Acyl Chloride** **N-(2-oxoethyl) alkylamide**

Scheme S5: Generic synthesis scheme depicting preparation of N-(2-oxoethyl)-alkylamides

<u>Generic alcohol preparation – Inverted Amide</u>

To a reactor equipped with temperature control, overhead stirring, and a reflux condenser was added glycolic acid, toluene, and the appropriate alkyl amine. Stirring was initiated and the reaction mixture was refluxed overnight. The reaction mixture was evaporated to dryness to yield crude product, typically as a dense yellow powder. To remove excess glycolic acid a 100 mg/mL solution of crude material in H_2O was prepared and stirred at 40 °C for 60 minutes, at which point undissolved material was collected via vacuum filtration using a fine fritted filter funnel (Chemglass®, CG-1402-12). The collected solids were then dried overnight in a vacuum oven to yield the target product, typically as a white solid.

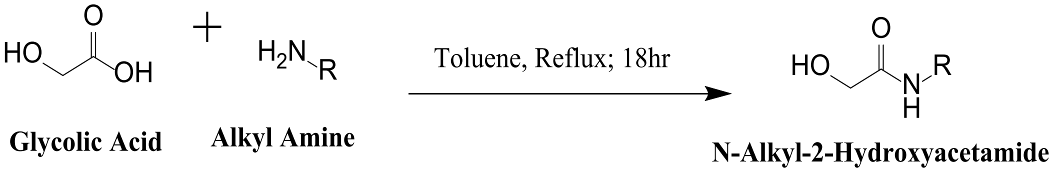

Scheme S6: Generic synthesis scheme depicting preparation of N-alkyl-2-hydroxyacetamides

<u>REPRESENTATIVE PREPARATION OF A CONVENTIONAL ESTER – RV65</u>

<u>2-hydroxyethyl dodecanoate</u>

To a reactor vessel was added N-Dimethylformamide (5 mL, 5 Vols), Triethylamine (1.7 mL, 12 mmol, 2.5 equiv.), Lauric acid (1g, 5.0 mmol, 1.0 equiv.) and 2-iodoethanol (880 µL, 10 mmol, 2.0 equiv.). The reaction mixture was then placed in an incubated shaker set at 40 °C and ~125 rpm where it was left to shake overnight. Solvent was removed under reduced pressure and the residue was subjected to liquid-liquid extraction using H_2O (40 mL) and hexanes (3x75 mL). Organic layers were combined, and the solvent was removed under reduced pressure. The crude material was purified via silica gel flash column chromatography using a gradient method with hexanes and ethyl acetate as the mobile phases. Fractions of interest were combined, and the solvent was removed under reduced pressure to produce the target compound, 2-hydroxyethyl dodecanoate as a thick oil.

^{1}H NMR (500 MHz, $CDCl_3$) δ 4.21 – 4.15 (m, 2H), 3.82 – 3.76 (m, 2H), 2.32 (t, *J* = 7.6 Hz, 2H), 1.60 (t, *J* = 7.3 Hz, 2H), 1.27 – 1.23 (m, 16H), 0.85 (t, *J* = 6.9 Hz, 3H). MS (ESI^+) calculated for $C_{14}H_{28}O_3$ $[M+H]^+$ *m/z*: 244.2038; found: 244.3.

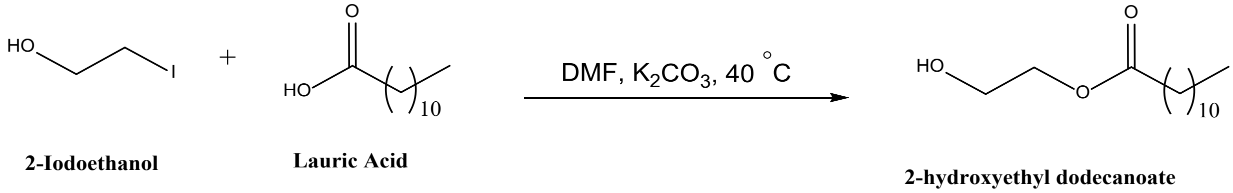

Scheme S7: Synthesis scheme describing the preparation of 2-hydroxyethyl dodecanoate

2-oxoethyl dodecanoate

To a reaction equipped with a stir bar was added 2-hydroxyethyl dodecanoate (180 mg, 0.75 mmol, 1 equiv.) and Dichloromethane (3.7 mL, 20 Vols.). The reaction mixture was stirred for approximately 5 minutes to fully dissolve the starting material at which point Dess-Martin periodinane (640 mg, 1.5 mmol 2.0 equiv.) was added to the reaction mixture. The mixture was allowed to stir for two hours at which point the reaction mixture was treated with a solution of 10% sodium thiosulfate saturated with $NaHCO_3$ for 90 minutes. The reaction mixture was then extracted with the sodium thiosulfate solutions (3 x 100 mL) and brine (2 x 100 mL), while retaining the organic layer. The organic layer was dried over Na_2SO_4, filtered, and solvent was removed under reduced pressure to yield the target product, 2-oxoethyl dodecanoate as an off-white solid. The final material was typically used without further purification; typically on the day of preparation.

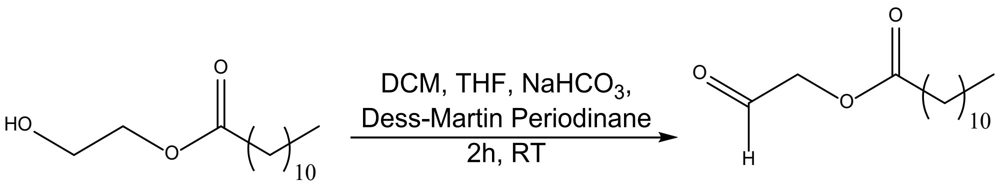

2-hydroxyethyl dodecanoate **2-oxoethyl dodecanoate**

Scheme S8: Synthesis scheme describing the preparation of 2-oxoethyl dodecanoate

RV65

To a stirred solution of DMF (20 mL) and DIPEA (240 µL, 1.4 mmol, 2.0 equiv.) at 65 °C was added vancomycin HCl (1.0 g, 690 µmol) and the mixture was stirred for 10 minutes. The mixture was then cooled to 30 °C at which point 2-oxoethyl dodecanoate was added (250 mg, 1.0 mmol, 1.5 equiv.). The resulting solution was stirred overnight at which point MeOH (10 mL) and TFA (210 µL, 7.8 mmol, 4 equiv.) were added. After stirring for 2 hours, Borane tert-butylamine complex (60 mg, 69 µmol, 1.0 equiv.) was added portion-wise. After stirring for an additional 2 hours the reaction mixture was then purified using reverse

phase C18 column chromatography (Phenomenex Luna 10 µM PREP C18(2) 250 x 21.2 mm column) using gradients of water and acetonitrile, each containing 0.1% (v/v) of TFA. In some instances, lactic acid was used instead of TFA to prepare lactic acid salt former of RV lipoglycopeptides. Fractions were evaluated using HPLC and then pertinent fractions containing purified RV65 were pooled together for removal of solvent via lyophilization. The target compound, RV65 (148 mg, 88 µmol, 13% overall yield), was obtained as a white solid in >97% purity (by HPLC).

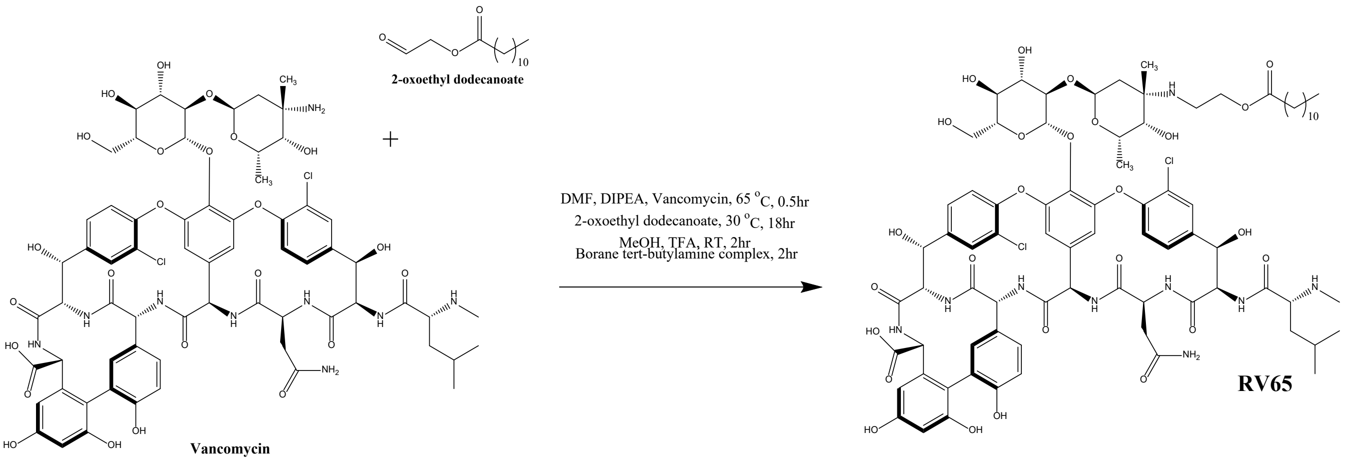

Scheme S9: Synthesis scheme depicting the preparation of RV65

REPRESENTATIVE PREPARATION OF AN INVERTED ESTER – RV55

Decyl 2-Hydroxyacetate

To a reactor vessel was added DMF (4.1 mL, 25 Vols.), K2CO3 (740 mg, 5.3 mmol, 2.5 equiv.), glycolic acid (163 mg, 2.1 mmol, 1.0 equiv. and 1-iododecans (630 mg, 2.3 mmol, 1.1 equiv.). The reaction mixture was then placed in an incubated shaker set at 40 °C and ~125 rpm where it was left to shake overnight. Solvent was removed under reduced pressure and the residue was subjected to liquid-liquid extraction using H_2O (40 mL) and hexanes (3 x 75 mL). Organic layers were combined, and the solvent was removed under reduced pressure. The crude material was purified via silica gel flash column chromatography using a gradient method with hexanes and ethyl acetate as the mobile phases. Fractions of interest were combined, and the solvent was removed under reduced pressure to produce the target compound, Decyl-2-Hydroxyacetate (140 mg, 650 μmol, 30% overall yield) as a thick oil.

^{1}H NMR (500 MHz, $CDCl_3$) δ 4.18 (t, *J* = 6.8 Hz, 2H), 4.14 (s, 2H), 2.57 (s, 1H), 1.65 (d, *J* = 7.0 Hz, 2H), 1.31 (dd, *J* = 19.5, 8.9 Hz, 6H), 1.25 (s, 8H), 0.87 (t, *J* = 6.9 Hz, 3H). MS (ESI^+) calculated for $C_{12}H_{24}O_3$ $[M+H]^+$ *m/z*: 216.3; found: 216.2.

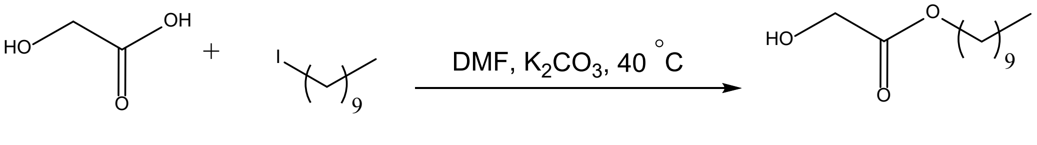

Glycolic Acid **1-Iododecane** **decyl 2-hydroxyacetate**

Scheme S10: Synthesis scheme depicting preparation of decyl 2-hydroxyacetate

Decyl 2-Oxoacetate

To a reactor vessel equipped with a stir bar was added decyl 2-hydroxyacetate (140 mg, 647 μmol, 1.0 equiv.), DCM (6 mL, 29 Vols), and THF (10 mL, 10 Vols). The reaction mixture was stirred for approximately 5 minutes to fully dissolve the starting material, at which point Dess-Martin periodinane (1.1 g, 780 μmol, 1.2 equiv.) was added to the reaction mixture. After stirring for 2 hours the solution was then poured into a

separatory funnel and the organic layer was isolated, washed with two additional aliquots of 10% sodium thiosulfate in water saturated with $NaHCO_3$ (3 x 100 mL), and brine (2 x 100 mL). The organic layer was then dried over Na_2SO_4, filtered, and evaporated to dryness to yield the target compound, Decyl 2-Oxoacetate, as a yellow tinged solid that was used without further purification; typically on the day of preparation.

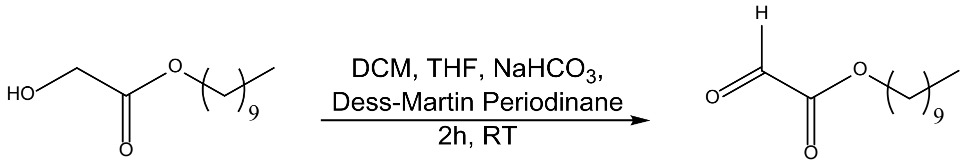

decyl 2-hydroxyacetate **decyl 2-oxoacetate**

Scheme S11: Synthesis scheme depicting preparation of decyl 2-oxoacetate

<u>RV55</u>

To a stirred solution of DMF (3 mL) and DIPEA (27 μL, 156 μmol, 2.0 equiv.) at 65 °C was added vancomycin HCl (113 mg, 78 μmol) and the mixture was stirred for 10 minutes. The mixture was then cooled to 30 °C at which point decyl 2-oxoacetate (25 mg, 117 μmol, 1.5 equiv.) was added. The resulting solution was stirred overnight at which point MeOH (3 mL) and TFA (24 μL, 310 μmol, 4 equiv.) were added. After stirring for 2 hours, Borane tert-butylamine complex (60 mg, 69 μmol, 1.0 equiv.) was added portion-wise. After stirring for an additional 2 hours the reaction mixture was then purified using reverse phase C18 column chromatography (Phenomenex Luna 10 μM PREP C18(2) 250 x 21.2 mm column) using gradients of water and acetonitrile, each containing 0.1% (v/v) of TFA. In some instances, lactic acid was used instead of TFA to prepare lactic acid salt former of RV lipoglycopeptides. Fractions were evaluated using HPLC and then pertinent fractions containing purified RV65 were pooled together for removal of solvent via lyophilization. The target compound, RV55 was obtained as a white solid in >97% purity (by HPLC).

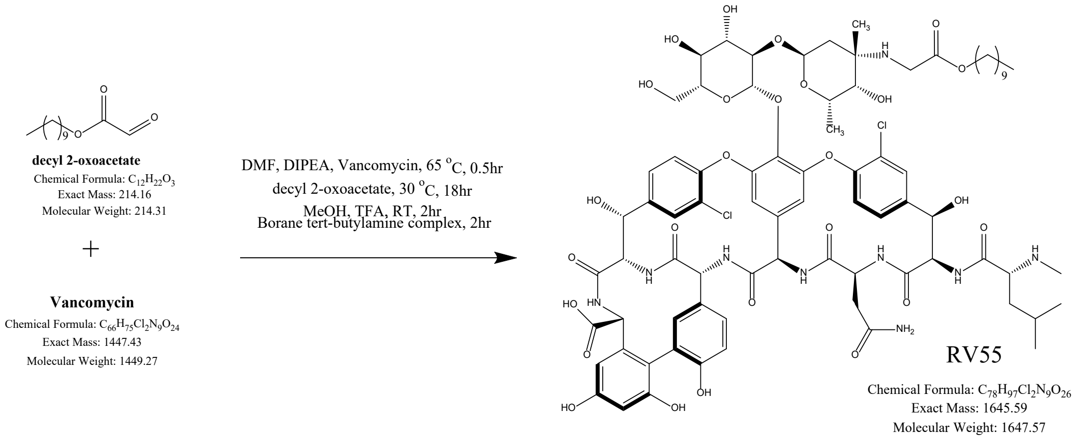

Scheme S12: Synthesis scheme depicting preparation of RV55

REPRESENTATIVE PREPARATION OF A CONVENTIONAL AMIDE – RV62

N-(2-hydroxyethyl)dodecanamide

To a reactor vessel were added ethanolamine (3.5 g, 56.7 mmol, 2.1 equiv.) and THF (150 mL, 25.4 Vols). The temperature was adjusted to be 0 °C and stirring was initiated. Once the temperature stabilized, triethylamine (5.6 mL, 40.5 mmol, 1.5 equiv.) was added in a single aliquot. Separately, a solution of dodecanoyl chloride (6.4 mL, 27.0 mmol, 1 equiv.) and THF (50 mL, 8.5 Vols) was prepared and charged into a dosing apparatus. The dodecanoyl chloride solution was added drop wise over the course of few hours, while stirring at 0 °C. The reaction mixture was warmed to 25 °C over a two-hour period and the reaction mixture was stirred for approximately 18 hours, at which point stirring was stopped. The reaction mixture was filtered to remove a white precipitate that had formed. Solvent was removed under reduced pressure to yield a thick, colorless oil. The crude material was dissolved in EtOAc (300 mL) and washed with 0.1M HCl (3 x 100 mL), saturated $NaHCO_3$ (3 x 100 mL), and brine (3 x 100 mL). The organic layer was dried over Na_2SO_4, filtered, and evaporated to dryness to yield the target compound, N-(2-hydroxyethyl)decanamide (3.2 g, 12.9 mmol, 48% overall yield) as a white solid. The crude material was purified using prep-HPLC with a CN column and an isocratic method with 10% isopropyl alcohol as the mobile phase. Pure fractions were combined, and solvent was removed to yield the target compound, N-(2-hydroxyethyl)decanamide (3.2 g, 12.9 mmol, 48% overall yield) as a white solid.

^1H NMR (500 MHz, $CDCl_3$) δ 6.13 (s, 1H), 3.73 – 3.68 (m, 2H), 3.41 (q, *J* = 5.4 Hz, 2H), 2.92 (s, 1H), 2.23 – 2.16 (m, 2H), 1.62 (t, *J* = 6.4 Hz, 3H), 1.28 - 1.24 (m, 16H), 0.87 (t, *J* = 6.9 Hz, 3H). ^{13}C NMR (126 MHz, $CDCl_3$) δ 174.62, 62.45, 42.48, 36.76, 31.91, 29.62, 29.50, 29.37, 29.34, 29.31, 29.16, 25.75, 22.69, 14.12. MS (ESI^+) calculated for $C_{14}H_{29}NO_2$ $[M+H]^+$ *m/z*: 243.2198; found: 243.219.

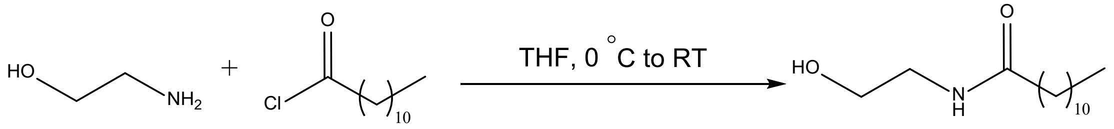

Scheme S13: Synthesis scheme depicting preparation of N-(2-hydroxyethyl)dodecanamide

N-(2-oxoethyl)dodecanamide

To a reactor vessel equipped with a stir bar was added N-(2-hydroxyethyl)dodecanamide (1.0 g, 4.1 mmol, 1.0 equiv.), DCM (20 mL, 20 Vols), and THF (10 mL, 10 Vols). The reaction mixture was stirred for approximately 5 minutes to fully dissolve the starting material, at which point $NaHCO_3$ (690 mg, 8.2 mmol, 2.0 equiv.) and Dess-Martin periodinane (2.2 g, 5.1 mmol, 1.3 equiv.) were added to the reaction mixture. After stirring for 2 hours the solution was then poured into a separatory funnel and the organic layer was isolated, washed with two additional aliquots of 10% sodium thiosulfate in water saturated with $NaHCO_3$ (3 x 100 mL), and brine (2 x 100 mL). The organic layer was then dried over Na_2SO_4, filtered, and evaporated to dryness to yield the target compound, N-(2-oxoethyl)dodecanamide (673 mg, 2.8 mmol, 67% overall yield), as a yellow tinged solid that was used without further purification; typically on the day of preparation.

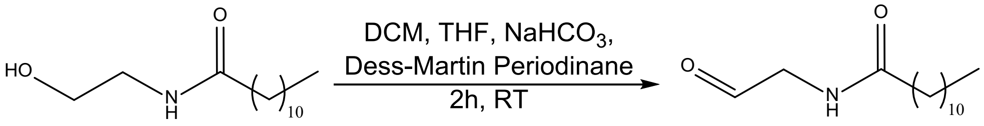

Scheme S14: Synthesis scheme depicting preparation of N-(2-oxoethyl)dodecanamide

Preparation of RV62

To a stirred solution of DMF (50 mL) and DIPEA (694 µL, 4.0 mmol) at 65 °C was added vancomycin HCl (2.9 g, 2.0 mmol) and the mixture was stirred for 10 minutes. The mixture was then cooled to 30 °C at which point N-(2-oxoethyl)dodecanamide was added (673 mg, 2.8 mmol). The resulting solution was stirred overnight at which point MeOH (25 mL) and TFA (610 µL, 8.0 mmol) were added. After stirring for 2 hours, Borane tert-butylamine complex (173 mg, 2.0 mmol) was added portion-wise. After stirring for an additional 2 hours the reaction mixture was then purified using reverse phase C18 column chromatography (Phenomenex Luna 10 µM PREP C18(2) 250 x 21.2 mm column) using gradients of water and acetonitrile, each containing 0.1% (v/v) of TFA. In some instances, lactic acid was used instead of TFA to prepare lactic acid salt former of RV lipoglycopeptides. Fractions were evaluated using HPLC and then pertinent fractions containing purified RV62 were pooled together for removal of solvent via lyophilization. The target

compound, RV62 (600 mg, 0.35 mmol, 18% overall yield), was obtained as a white solid in >97% purity (by HPLC).

^{1}H NMR (500 MHz; DMSO-d6): δ 9.40 (s, 1H), 9.13 (s, 1H), 9.05 (s, 1H), 8.62 (s, 1H), 8.49 (d, *J* = 5.7 Hz, 1H), 8.24 (s, 1H), 8.00 (t, *J* = 5.6 Hz, 1H), 7.84 (d, *J* = 1.9 Hz, 1H), 7.51 (dd, *J* = 8.5, 1.8 Hz, 1H), 7.46 (dd, *J* = 8.2, 1.9 Hz, 1H), 7.40 (s, 1H), 7.35 (s, 1H), 7.31 (d, *J* = 8.3 Hz, 1H), 7.24 (d, *J* = 8.4 Hz, 1H), 7.18 (s, 1H), 6.91 (s, 1H), 6.78 (d, *J* = 8.4 Hz, 1H), 6.71 (d, *J* = 8.4 Hz, 1H), 6.66 (d, *J* = 11.7 Hz, 1H), 6.39 (d, *J* = 2.3 Hz, 1H), 6.26 (d, *J* = 2.2 Hz, 1H), 5.93 (s, 1H), 5.79 (s, 1H), 5.74 (d, *J* = 8.1 Hz, 1H), 5.59 – 5.55 (m, 1H), 5.31 (dd, *J* = 23.1, 5.9 Hz, 2H), 5.21 – 5.17 (m, 1H), 5.16 (s, 1H), 5.11 (s, 1H), 4.90 (s, 1H), 4.64 (q, *J* = 6.4 Hz, 1H), 4.45 (s, 1H), 4.44 (d, *J* = 5.3 Hz, 1H), 4.33 (s, 1H), 4.23 – 4.15 (m, 2H), 4.08 – 3.98 (m, 4H), 3.69 (d, *J* = 11.0 Hz, 2H), 3.61 – 3.43 (m, 4H), 2.85 – 2.80 (m, 1H), 2.76 (s, 1H), 2.40 (s, 3H), 2.18 – 2.10 (m, 2H), 2.06 (t, *J* = 7.5 Hz, 2H), 1.92 – 1.86 (m, 1H), 1.81 (d, *J* = 13.1 Hz, 1H), 1.70 (hept, *J* = 6.7 Hz, 1H), 1.50 – 1.42 (m, 6H), 1.37 (d, *J* = 7.1 Hz, 2H), 1.34 – 1.28 (m, 2H), 1.28 – 1.19 (m, 20H), 1.14 (dd, *J* = 6.9, 0.8 Hz, 2H), 1.09 (d, *J* = 6.4 Hz, 2H), 0.96 – 0.85 (m, 2H), 0.85 (d, *J* = 4.5 Hz, 3H). ^{13}C NMR (126 MHz, DMSO) δ 179.25, 176.49, 174.03, 173.29, 172.51, 172.26, 171.15, 170.61, 169.43, 169.12, 167.73, 166.91, 158.14, 157.90, 157.16, 156.48, 155.06, 152.14, 151.31, 149.87, 148.27, 142.43, 139.82, 136.12, 135.62, 134.54, 131.78, 128.63, 127.36, 127.08, 126.24, 126.20, 125.48, 124.22, 123.30, 121.58, 118.41, 117.99, 116.21, 116.03, 107.29, 105.74, 104.60, 102.33, 100.89, 96.52, 77.91, 77.00, 76.80, 71.54, 70.20, 69.07, 65.87, 65.65, 63.11, 61.74, 61.20, 58.45, 56.72, 54.85, 53.64, 50.99, 36.03, 35.21, 32.71, 31.24, 28.93, 28.85, 28.69, 28.64, 28.60, 27.09, 24.97, 23.95, 22.65, 22.55, 22.04, 20.49, 20.33, 18.99, 16.90, 16.79, 13.91, 0.00 (TMS). LC-MS (ESI^{+}) calculated for $C_{80}H_{102}Cl_2N_{10}O_{25}$ [M+H]$^{+}$ *m/z*: 1672.6395; found: 1672.638; retention time = 19.2-19.4 minutes.

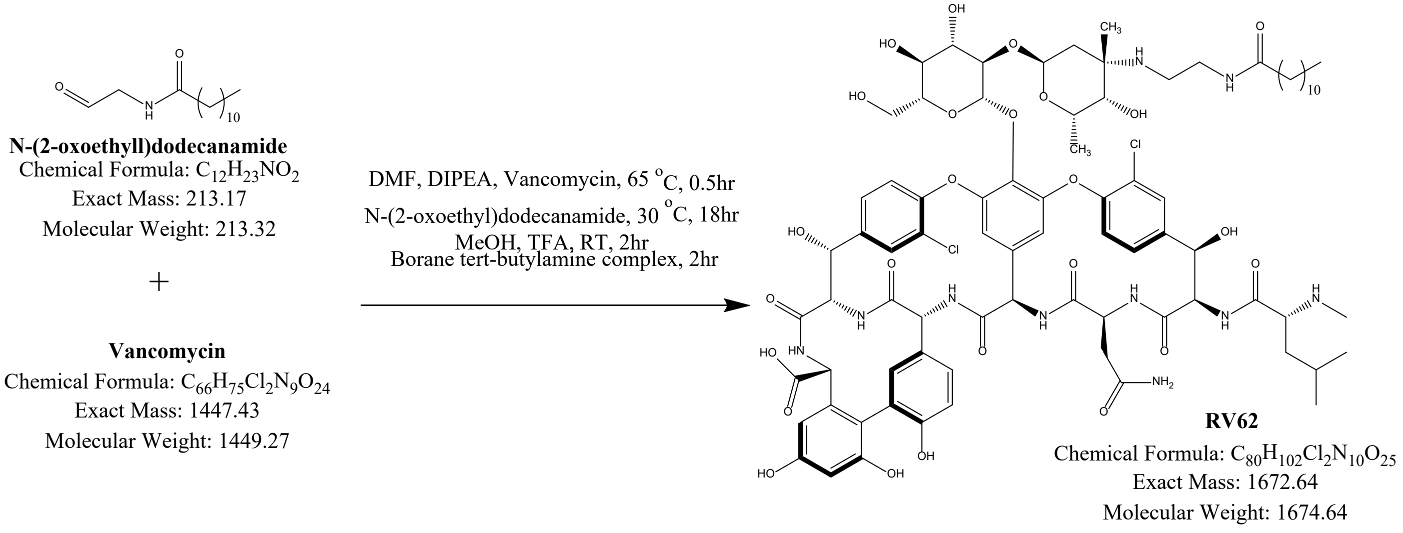

Scheme S15: Synthesis scheme depicting preparation of RV62

REPRESENTATIVE PREPARATION OF AN INVERTED AMIDE – RV94

N-Decyl-2-Hydroxyacetamide

To a chemical reactor equipped with temperature control, overhead stirring, and a reflux condenser was added glycolic acid (17.2 g, 227 mmol, 1.4 equiv.), toluene (350 mL, 0.462 M, 11.7 Vols), and N-Decylamine (30.0 g, 37.2 mL, 162 mmol, 1.0 equiv.). Stirring was initiated at 500 rpm and the reaction mixture was refluxed overnight. The reaction mixture was then evaporated to dryness to yield crude product as a dense yellow powder. To remove excess glycolic acid a 100 mg/mL solution of crude material in H_2O was prepared and stirred at 40 °C for 60 minutes, at which point undissolved material was collected via vacuum filtration using a fine fritted filter funnel (ChemGlass, CG-1402-12). The collected solids were then dried overnight in a vacuum oven to yield the target product, N-Decyl-2-Hydroxyacetamide, as a white solid in 58.4 % overall yield (23.0 g, 94.5 mmol). ^{1}H NMR (500 MHz, $CDCl_3$) δ 6.76 (s, 1H), 4.03 (s, 2H), 3.26 (q, *J* = 6.7 Hz, 2H), 1.50 (q, *J* = 7.2 Hz, 2H), 1.28 (s, 5H), 1.24 (s, 8H), 0.86 (t, *J* = 7.0 Hz, 3H). ^{13}C NMR (126 MHz, $CDCl_3$) δ 172.28, 77.41, 77.16, 76.90, 62.13, 39.20, 31.99, 31.70, 29.66, 29.61, 29.42, 29.41, 27.02, 22.79, 14.23. MS (ESI$^+$) calculated for $C_{12}H_{25}NO_2$ $[M+H]^+$ *m/z*: 215.1885; found: 215.189.

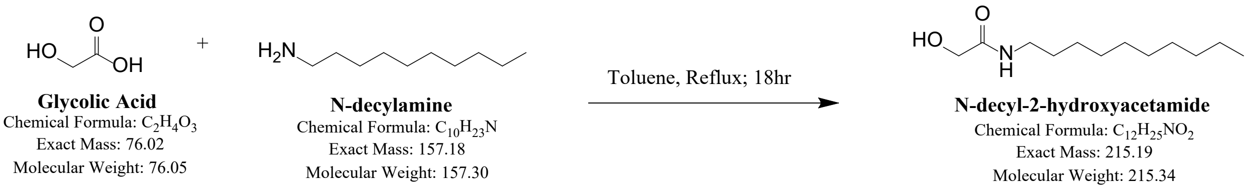

Scheme S16: Synthesis scheme depicting preparation of N-Decyl-2-hydroxyacetamide

N-Decyl-2-Oxoacetamide

To a reactor vessel equipped with temperature control, overhead stirring, and nitrogen purging was added N-Decyl-2-Hydroxyacetamide (10.6 g, 49.1 mmol, 1.0 equiv.), $NaHCO_3$ (4.54 g, 54.0 mmol, 1.1 equiv.), and DCM (264 mL, 25 Vols). Stirring at 400 rpm was initiated and the temperature was set to 5 °C. Once the alcohol starting material had fully dissolved and the temperature had equilibrated, Dess-Martin Periodinane (25.0 g, 58.9 mmol, 1.2 equiv.) was added portion-wise to the reaction mixture. The reaction

mixture was allowed to stir at 5 °C for 30 minutes. To the reaction mixture was added a solution of 10% sodium thiosulfate in water saturated with $NaHCO_3$ (150 mL). The reaction mixture was allowed to stir at 250 rpm and 25 °C for 30 minutes. The solution was then poured into a separatory funnel and the organic layer was isolated, washed with two additional aliquots of 10% sodium thiosulfate in water saturated with $NaHCO_3$ (2 x 150 mL), and brine (1 x 150 mL). The organic layer was then dried over Na_2SO_4, filtered, and evaporated to dryness to yield the target compound, N-Decyl-2-Oxoacetamide (4.6 g, 3.76 mmol, 44% overall yield), as a yellow tinged solid that was used without further purification; typically on the day of preparation.

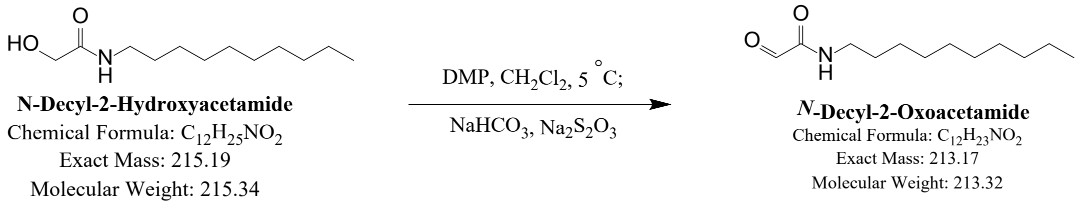

Scheme S17: Synthesis scheme depicting preparation of N-Decyl-2-Oxoacetamide

RV94

To a stirred solution of DMF (225 mL) and DIPEA (3.27 mL, 18.8 mmol) at 65 °C was added vancomycin HCl (13.6 g, 9.38 mmol) and the mixture was stirred for 10 minutes. The mixture was then cooled to 30 °C at which point N-Decyl-2-Oxoacetamide was added (2.2 g, 10.3 mmol). The resulting solution was stirred overnight at which point MeOH (100 mL) and TFA (2.87 mL, 37.5 mmol) were added. After stirring for 2 hours, Borane tert-butylamine complex (815 mg, 9.38 mmol) was added portion-wise. After stirring for an additional 2 hours the reaction mixture was then purified using reverse phase C18 column chromatography (Phenomenex Luna 10 µM PREP C18(2) 250 x 21.2 mm column) using gradients of water and acetonitrile, each containing 0.1% (v/v) of TFA. In some instances, lactic acid was used instead of TFA to prepare lactic acid salt former of RV lipoglycopeptides. Fractions were evaluated using HPLC and then pertinent fractions containing purified RV94 were pooled together for removal of solvent via lyophilization. The target compound, RV94 (6.2 g, 3.76 mmol, 40% overall yield), was obtained as a white solid.

^{1}H NMR (500 MHz; DMSO-d6): δ 9.40 (s, 1H), 9.12 (s, 1H), 9.05 (s, 1H), 8.62 (s, 1H), 8.49 (d, *J* = 5.8 Hz, 1H), 8.21 (s, 1H), 8.04 (s, 1H), 7.84 (d, *J* = 1.9 Hz, 1H), 7.51 (dd, *J* = 8.6, 1.7 Hz, 1H), 7.46 (dd, *J* = 8.4, 1.8 Hz, 1H), 7.39 (s, 1H), 7.34 – 7.27 (m, 1H), 7.23 (d, *J* = 8.4 Hz, 1H), 7.18 (d, *J* = 2.3 Hz, 1H), 6.90 (s, 1H), 6.78 (dd, *J* = 8.4, 2.1 Hz, 1H), 6.74 – 6.63 (m, 1H), 6.66 (s, 1H), 6.39 (d, *J* = 2.3 Hz, 1H), 6.26 (d, *J* = 2.2 Hz, 1H), 5.92 (d, *J* = 6.3 Hz, 1H), 5.77 (s, 1H), 5.75 – 5.71 (m, 1H), 5.57 (s, 1H), 5.32 (d, *J* = 7.7 Hz, 1H), 5.23 (s, 1H), 5.19 (d, *J* = 2.0 Hz, 1H), 5.15 (s, 1H), 5.10 (d, *J* = 5.9 Hz, 1H), 5.05 (s, 1H), 4.90 (s, 1H), 4.64 (q, *J* = 6.6 Hz, 1H), 4.44 (t, *J* = 5.9 Hz, H), 4.34 (s, 1H), 4.29 – 4.14 (m, 1H), 4.02 (q, *J* = 6.8 Hz, 2H), 3.69 (d, *J* = 11.3 Hz, 2H), 3.61 – 3.49 (m, 2H), 3.45 (s, 2H), 3.15 (s, 1H), 3.06 (q, *J* = 6.6 Hz, 2H), 2.46 – 2.40 (m, 2H), 2.39 (s, 3H), 2.19 – 2.10 (m, 2H), 1.82 (d, *J* = 12.5 Hz, 1H), 1.77 – 1.66 (m, 2H), 1.49 (dq, *J* = 37.1, 7.0 Hz, 2H), 1.38 (dd, *J* = 8.1, 5.1 Hz, 2H), 1.31 – 1.18 (m, 18H), 1.07 (d, *J* = 6.3 Hz, 2H), 0.93 – 0.85 (m, 2H), 0.85 (d, *J* = 5.0 Hz, 3H). ^{13}C NMR (126 MHz; DMSO-d6); δ 177.68, 176.45, 173.96, 172.44, 172.19, 171.00, 170.55, 169.42, 169.05, 167.80, 167.67, 166.95, 158.00, 157.76, 157.09, 156.41, 154.99, 152.07, 151.22, 149.88, 148.24, 142.30, 139.70, 136.07, 135.54, 134.34, 131.80, 128.57, 127.27, 127.05, 126.21, 126.15, 125.41, 124.15, 123.23, 121.52, 117.92, 116.14, 107.19, 105.69, 104.54, 102.27, 100.93,

96.99, 77.73, 76.99, 76.67, 71.49, 71.08, 70.22, 65.83, 65.59, 63.30, 61.69, 61.36, 61.18, 58.35, 56.66, 56.09, 54.78, 53.57, 50.92, 33.57, 32.85, 31.17, 28.84, 28.57, 27.33, 27.03, 26.21, 23.93, 22.66, 22.45, 21.98, 21.66, 20.44, 20.27, 20.18, 17.61, 16.97, 13.85, 0.00 (TMS). LC-MS (ESI$^+$) calculated for $C_{78}H_{98}Cl_2N_{10}O_{25}$ [M+H]$^+$ *m/z*: 1644.6082; found: 1644.607; retention time = 18.4-18.8 minutes.

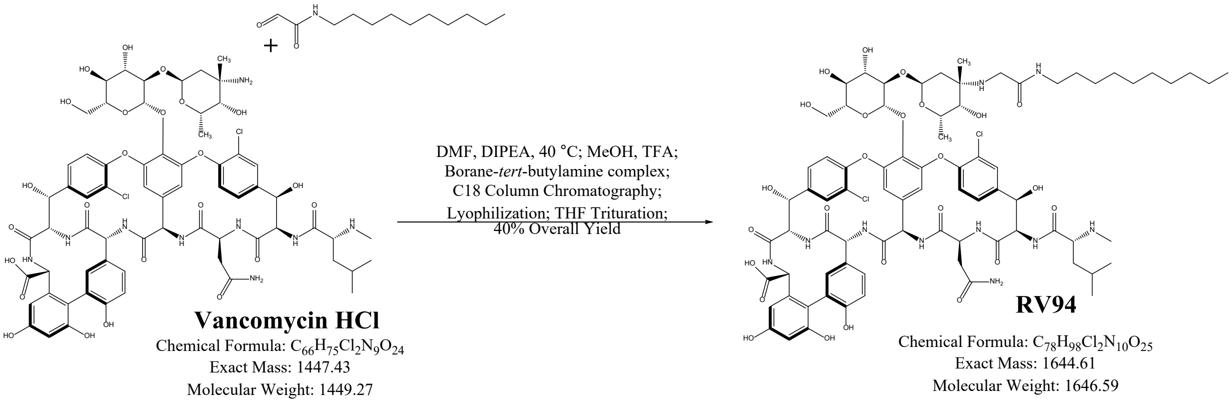

Scheme S18: Synthesis scheme depicting preparation of RV94

PREPARATION OF RV LIPOGLYCOPEPTIDE METABOLITES

RV80

To a stirred solution of DMF (40 mL) and DIPEA (5.0 mmol) at 65 °C was added vancomycin HCl (4.0 g, 2.7 mmol) and the mixture was stirred for 30 minutes. The mixture was then cooled to 30 °C at which point 2-[(tert-butyldimethylsilyl)oxy]acetaldehyde was added (2.8 mmol). The resulting solution was stirred overnight at which point MeOH (20 mL) and TFA (10 mmol) were added. After stirring for 2 hours, Borane tert-butylamine complex (2.0 mmol) was added portion-wise. After stirring for an additional 2 hours the reaction mixture was then purified using reverse phase C18 column chromatography (Phenomenex Luna 10 µM PREP C18(2) 250 x 21.2 mm column) using gradients of water and acetonitrile, each containing 0.1% (v/v) of TFA. Fractions were evaluated using HPLC and then pertinent fractions containing purified RV80-silyl-ether were pooled together. The RV80-silyl-ether was hydrolyzed during short term storage (4 hours) at room temperature. Crude material was isolated by lyophilization and was then purified using reverse phase C18 column chromatography (Phenomenex Luna 10 µM PREP C18(2) 250 x 21.2 mm column) using gradients of water and acetonitrile, each containing 0.1% (v/v) of TFA. Fractions were evaluated using HPLC and then pertinent fractions containing purified RV80 were pooled together for the isolation of the product via lyophilization.

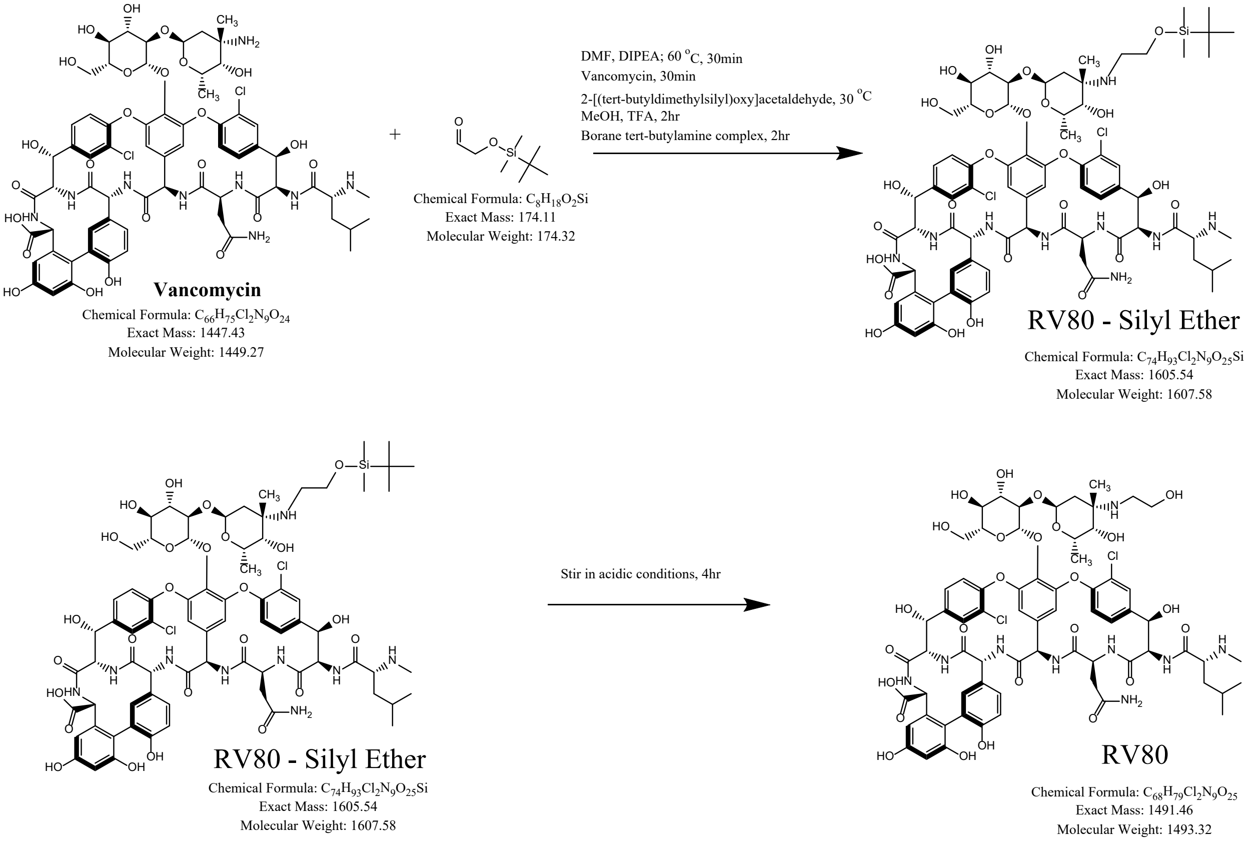

Scheme S19: Synthesis scheme depicting preparation of RV80.

RV82

Preparation of 9H-fluoren-9-ylmethyl N-(2-oxoethyl)carbamate

To a 400 mL reactor vessel, equipped with pH monitoring, overhead stirring, temperature control, inert gas, and a dosing apparatus, were added DCM (250 mL) and 9H-fluoren-9-ylmethyl N-(2-hydroxyethyl)carbamate (3.00 g, 10.6 mmol). The resulting solution was stirred at 25 °C for 30 minutes at which point the temperature was then reduced to 15 °C and Dess-Martin Periodinane (4.94 g, 11.7 mmol) was added. The reaction mixture was stirred overnight. The reaction mixture was diluted with DCM (150 mL) and was then washed with a 10% Sodium thiosulfate solution saturated with sodium bicarbonate (2 x 400mL), saturated sodium bicarbonate (2 x 400mL), and brine (1 x 400). The organic layer was then dried over anhydrous sodium sulfate, filtered, and evaporated to dryness to yield the target compound (500mg, 1.78mmol, 16.8 % yield) as a white solid that was used without further purification.

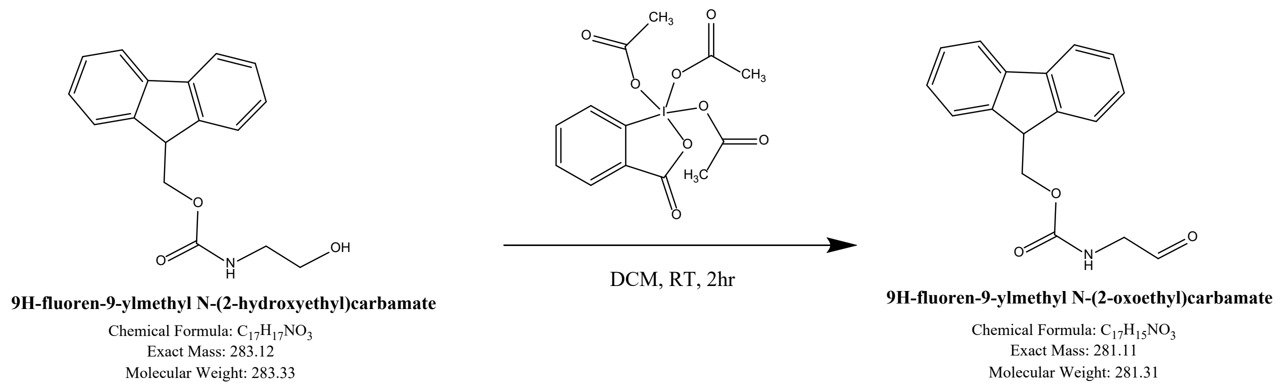

Scheme S20: Synthesis scheme depicting preparation of 9H-fluoren-9-ylmethyl N-(2-oxoethyl)carbamate

Preparation of RV82-FMOC

To a stirred solution of DMF (225 mL) and DIPEA (16.6 mmol) at 65 °C was added vancomycin HCl (12.0 g, 8.28 mmol) and the mixture was stirred for 30 minutes. The mixture was then cooled to 30 °C at which point 9H-fluoren-9-ylmethyl N-(2-oxoethyl)carbamate (2.33 g, 8.28 mmol) was added. The resulting solution was stirred overnight at which point MeOH (125 mL) and TFA (33.1 mmol) were added. After stirring for 2 hours, Borane tert-butylamine complex (8.28 mmol) was added portion-wise. After stirring for an additional 2 hours the reaction mixture was then purified using reverse phase C18 column chromatography (Phenomenex Luna 10 µM PREP C18(2) 250 x 21.2 mm column) using gradients of water

and acetonitrile, each containing 0.1% (v/v) of TFA. Fractions were evaluated using HPLC and then pertinent fractions containing purified RV82-FMOC were pooled together for the isolation of the product via lyophilization. The target compound, RV82-FMOC was obtained as a white solid (500mg, 0.28 mmol, 16.4 % yield) in >98% purity (HPLC).

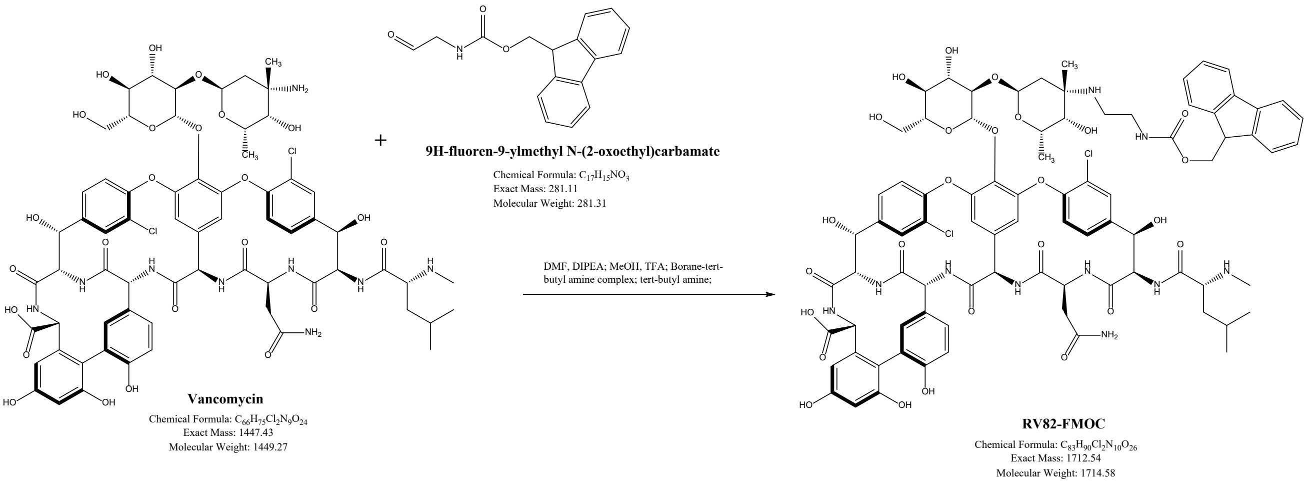

Scheme S21: Synthesis scheme depicting preparation of RV82-FMOC

Preparation of RV82.

To a slurry of purified RV82-FMOC (400 mg, 0.233 mmol) in MeCN (100 mL) was added piperidine (2.33 mmol) and the reaction mixture was stirred for 4 hours at room temperature. Crude material was purified via Flash Column chromatography (Biotage HP-Sphere C18 25 uM, SNAP Ultra 120g column) using gradients of water and acetonitrile, each containing 0.1% (v/v) of Lactic Acid as the mobile phases. Collected fractions were evaluated using HPLC, and pertinent fractions containing RV82 were pooled together for the isolation of the product via lyophilization. The target compound, RV82 was obtained as a white solid (240 mg, 0.161 mmol, 69 % yield) in >98% purity (HPLC).

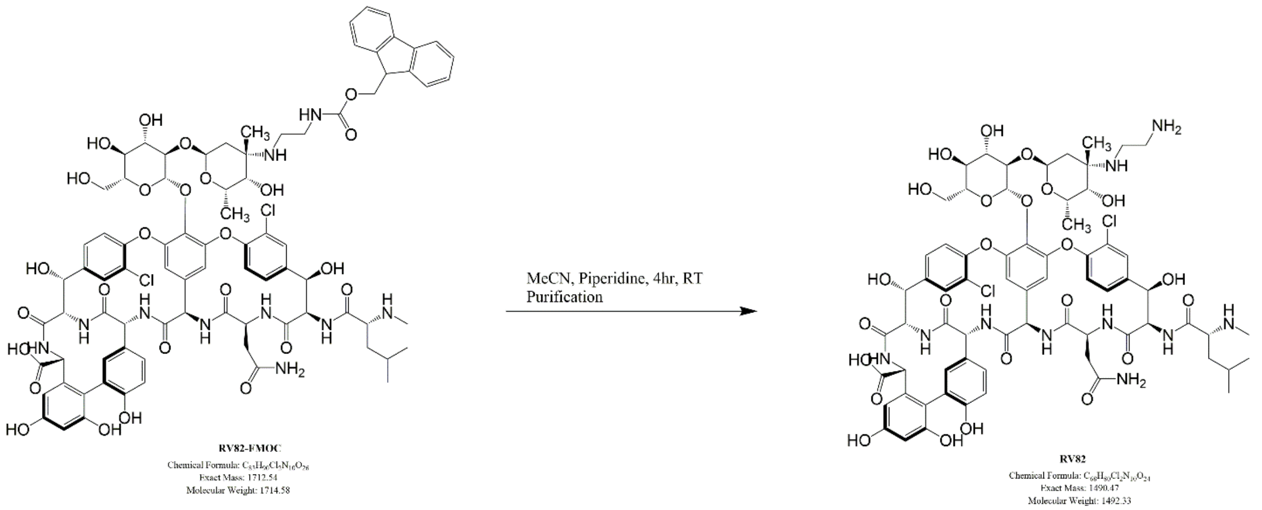

Scheme S22: Synthesis scheme depicting preparation of RV82.

RV101

To a solution of 30% MeCN in H_2O containing 0.1% TFA v/v (15 mL) at 35 °C was added RV94 (350 mg) via portion-wise addition. The colorless solution was stirred, and the pH was adjusted to 10 via addition of NaOH. The reaction was allowed to stir overnight at 35 °C at which point the reaction mixture was purified using Flash Column chromatography (Biotage HP-Sphere C18 25 uM, SNAP Ultra 120g column) using gradients of water and acetonitrile, each containing 0.1% (v/v) of Lactic Acid, as the mobile phases. Fractions were evaluated using HPLC, and then pertinent fractions containing RV101 were pooled together for the isolation of the product via lyophilization. The target compound, RV101 was obtained as a yellow solid (210mg, 0.139 mmol, 65.5 % yield) in >98% purity (by HPLC).

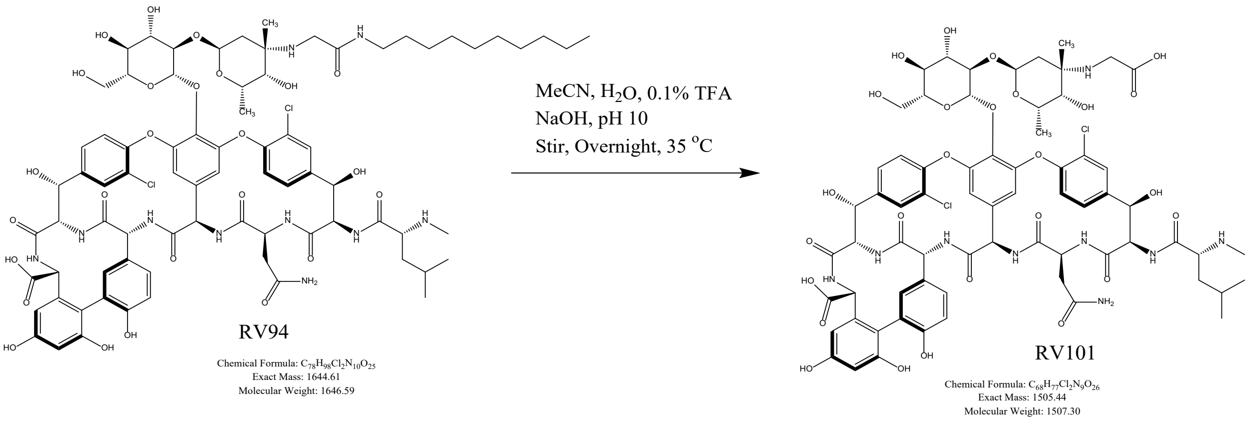

Scheme S23: Synthesis scheme depicting preparation of RV101.

PREPARATION OF RV LIPOGLYCOPEPTIDE COMPARATORS

RV40

To a stirred solution of DMF (250 mL) at 60 °C was added DIPEA (5.9 mL, 34 mmol) and vancomycin HCl (25.2 g, 17 mmol) and the mixture was stirred for 10 minutes. The mixture was then cooled to 30 °C at which point a solution of N-Fmoc-N-Decylaminoacetaldehyde (8.5 g, 20 mmol) dissolved in DMF (20 mL) was added. The resulting solution was stirred overnight at which point sodium cyanoborohydride (3.2 g, 51 mmol) was added and the mixture was allowed to stir for 5 minutes prior to addition of TFA (3.9 mL, 51 mmol) dissolved in MeOH (250 mL). The reaction mixture was stirred for one hour at which point methanol was removed under reduced pressure. To the concentrated solution was then added piperidine (8.3 mL, 84 mmol) and the reaction mixture was stirred for an additional hour. The reaction mixture was then purified using reverse phase C18 column chromatography (Phenomenex Luna 10 µM PREP C18(2) 250 x 21.2 mm column) using gradients of water and acetonitrile, each containing 0.1% (v/v) of TFA. In some instances, lactic acid was used instead of TFA to prepare lactic acid salt former of RV40. Fractions were evaluated using HPLC and then pertinent fractions containing purified RV40 were pooled together and lyophilized to yield the target product, RV40 (8.2 g, 5.0 mmol, 25% overall yield) as a fluffy white solid.

^{1}H NMR (500 MHz, DMSO) δ

9.40 (s, 1H), 9.06 (s, 1H), 8.61 (s, 1H), 8.48 (d, *J* = 5.8 Hz), 8.21 (s, 1H), 7.96 (s, 1H), 7.83 (s, 1H), 7.51 (dd, *J* = 8.6, 1.7 Hz, 1H), 7.45 (dd, *J* = 8.4, 1.8 Hz, 1H), 7.38 (s, 1H), 7.33 (s, 1H), 7.29 – 7.27 (m, 1H), 7.23 (d, *J* = 8.4 Hz, 1H), 7.18 (d, J = 8.4 Hz, 1H), 6.88 (s, 1H), 6.78 (dd, *J* = 8.4, 2.1 Hz, 1H), 6.69 (dd, *J* = 8.4, 2.1 Hz, 1H), 6.4 (m, 1H), 6.16 (m, 1H), 5.92 (s, 1H), 5.74 (d, *J* = 8.1 Hz, 1H), 5.56 (s, 1H), 5.33 (d, *J* = 7.7 Hz, 1H), 5.23 (d, *J* = 4.0 Hz, 1H), 5.19 (d, *J* = 2.1 Hz, 1H), 5.15 (d, *J* = 4.3 Hz, 1H), 5.10 (s, 1H), 4.88 (s, 1H), 4.64 (q, *J* = 6.5 Hz, 1H), 4.44 (t, *J* = 6.4 Hz, 1H), 4.35 (s, 1H), 4.18 (dq, *J* = 17.2, 7.3, 6.9 Hz, 2H), 4.00 (q, *J* = 6.9 Hz, 2H), 3.69 (d, *J* = 10.8 Hz, 2H), 3.61 – 3.49 (m, 4H), 3.45 (t, *J* = 8.8 Hz, 2H), 3.30 – 3.18 (m, 2H), 3.21 (m, 2H), 2.84 (d, *J* = 6.2 Hz, 2H), 2.75 – 2.68 (m, 4H), 2.37 (s, 2H), 2.14 (m, 1H), 1.70 (h, *J* = 6.0, 5.1 Hz, 2H), 1.53 (m, 2H), 1.49 (s, 2H), 1.43 (dt, *J* = 14.0, 7.2 Hz, 2H), 1.36 (d, *J* = 7.1 Hz, 2H), 1.28 (dd, *J* = 6.9, 1.7 Hz, 3H), 1.26 – 1.19 (m, 12H), 1.08 (d, *J* = 6.3 Hz, 2H), 0.90 (d, *J* = 6.6 Hz, 2H), 0.88 –

0.84 (m, 3H). ^{13}C NMR (126 MHz, DMSO) δ 176.75, 173.98, 172.79, 172.56, 172.25, 170.94, 170.59, 169.45, 169.05, 167.65, 166.98, 158.10, 157.86, 157.08, 156.40, 154.99, 152.07, 151.26, 149.85, 148.26, 142.29, 139.69, 136.16, 135.61, 134.33, 131.80, 128.54, 127.23, 127.23, 127.03, 126.19, 126.14, 124.08, 123.25, 121.54, 118.33, 117.92, 115.95, 106.93, 106.03, 105.40, 104.50, 102.51, 102.12, 100.86, 97.09, 77.81, 77.16, 76.83, 71.48, 71.05, 70.37, 66.24, 65.63, 63.45, 63.11, 61.89, 61.49, 61.19, 58.35, 56.67, 55.43, 54.83, 53.47, 50.84, 47.19, 45.82, 36.47, 31.17, 28.79, 28.55, 26.38, 26.03, 24.17, 23.68, 22.51, 22.49, 21.98, 20.49, 17.10, 13.91, 0.00 (TMS). LC-MS (ESI^+) calculated for $C_{78}H_{100}Cl_2N_{10}O_{24}$ $[M+H]^+$ *m/z*: 1630.6289; found: 1630.630; retention time = 17.9 minutes.

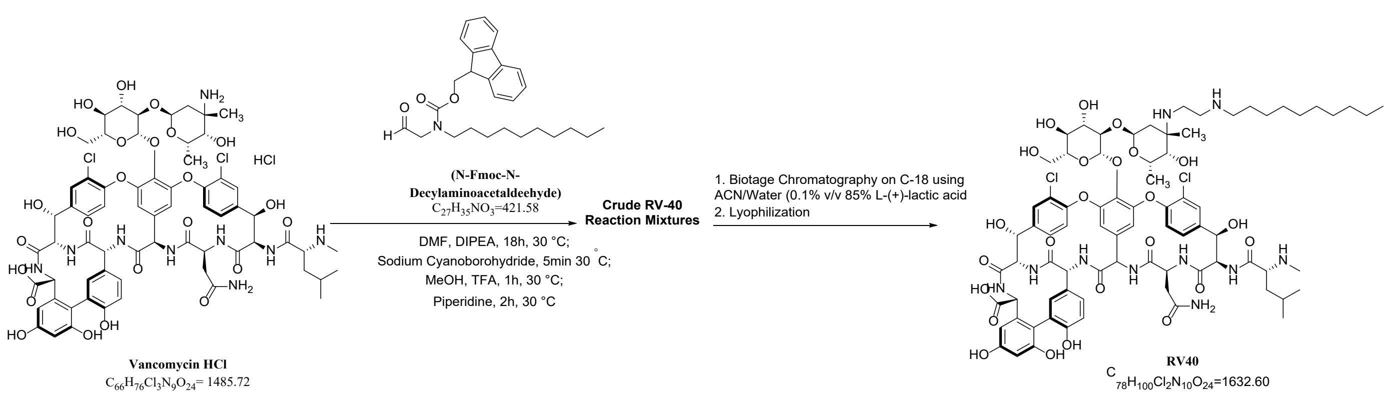

Scheme S24: Synthesis of RV40

Telavancin (TLV)

To a reactor vessel equipped with overhead stirring, temperature control, and nitrogen purging was added H_2O (12 mL), acetonitrile (48 mL), and DIPEA (0.64 mL, 2.7 mmol, 20 equiv.). The reaction temperature was reduced to 0 °C at which point Aminomethylphosphonic acid (250 mg, 2.2 mmol, 12 equiv.) and a 37% formaldehyde solution (25 µL, 920 µmol, 5 equiv.) were added. The reaction mixture was allowed to stir for 60 minutes at which point RV40 (300 mg, 180 µmol, 1 equiv.) was added to the reaction mixture. The reaction mixture was allowed to stir overnight at which point acetonitrile (150 mL) was added to induce precipitation. The crude product was isolated via vacuum filtration and was purified using reverse phase C18 column chromatography (Phenomenex Luna 10 µM PREP C18(2) 250 x 21.2 mm column) using gradients of water and acetonitrile, each containing 0.1% (v/v) of TFA. Fractions were evaluated using HPLC and then pertinent fractions containing purified TLV were pooled together and lyophilized to yield the target product, TLV (180 mg, 100 mmol, 57% overall yield) as a fluffy white solid.

LC-MS (ESI^+) calculated for $C_{80}H_{106}Cl_2N_{11}O_{27}P$ $[M+H]^+$ *m/z*: 1753.6374; found: 1753.637; retention time = 13.3 minutes.

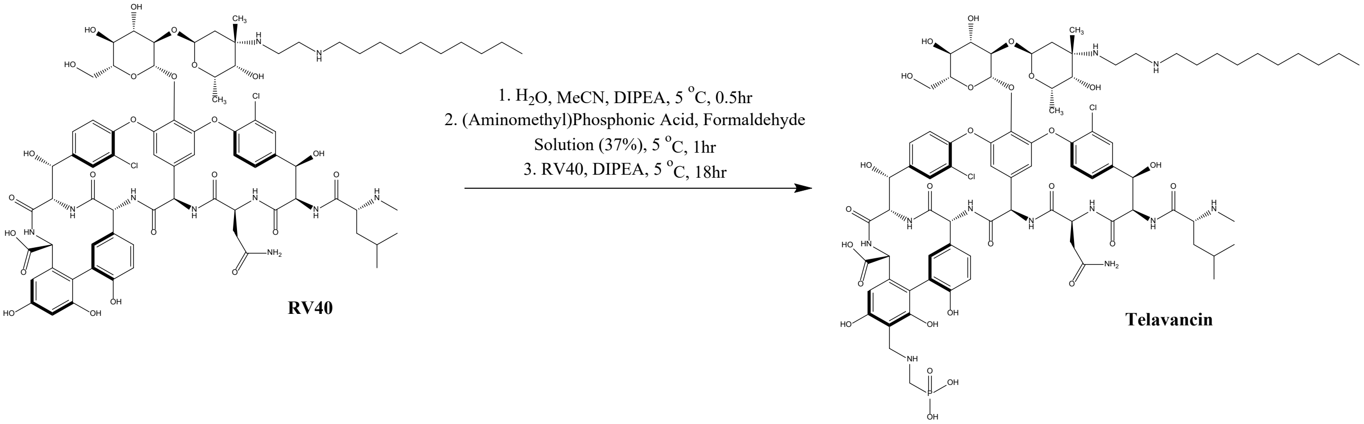

Scheme S25: Synthesis of Telavancin

CHEMICAL CHARACTERIZATION

NMR SPECTRA

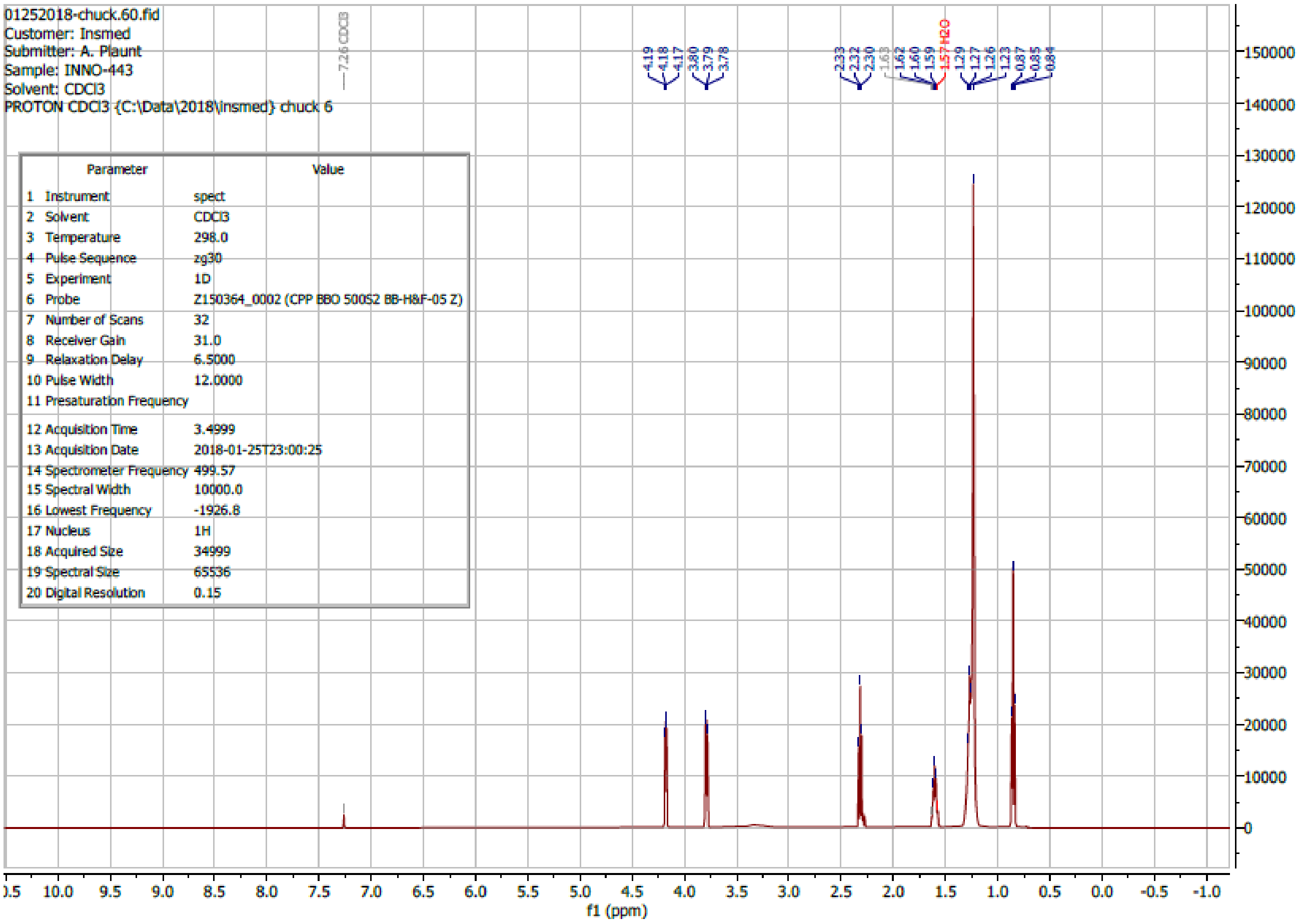

Figure S6: 1H NMR of 2-Hydroxyethyl Dodecanoate

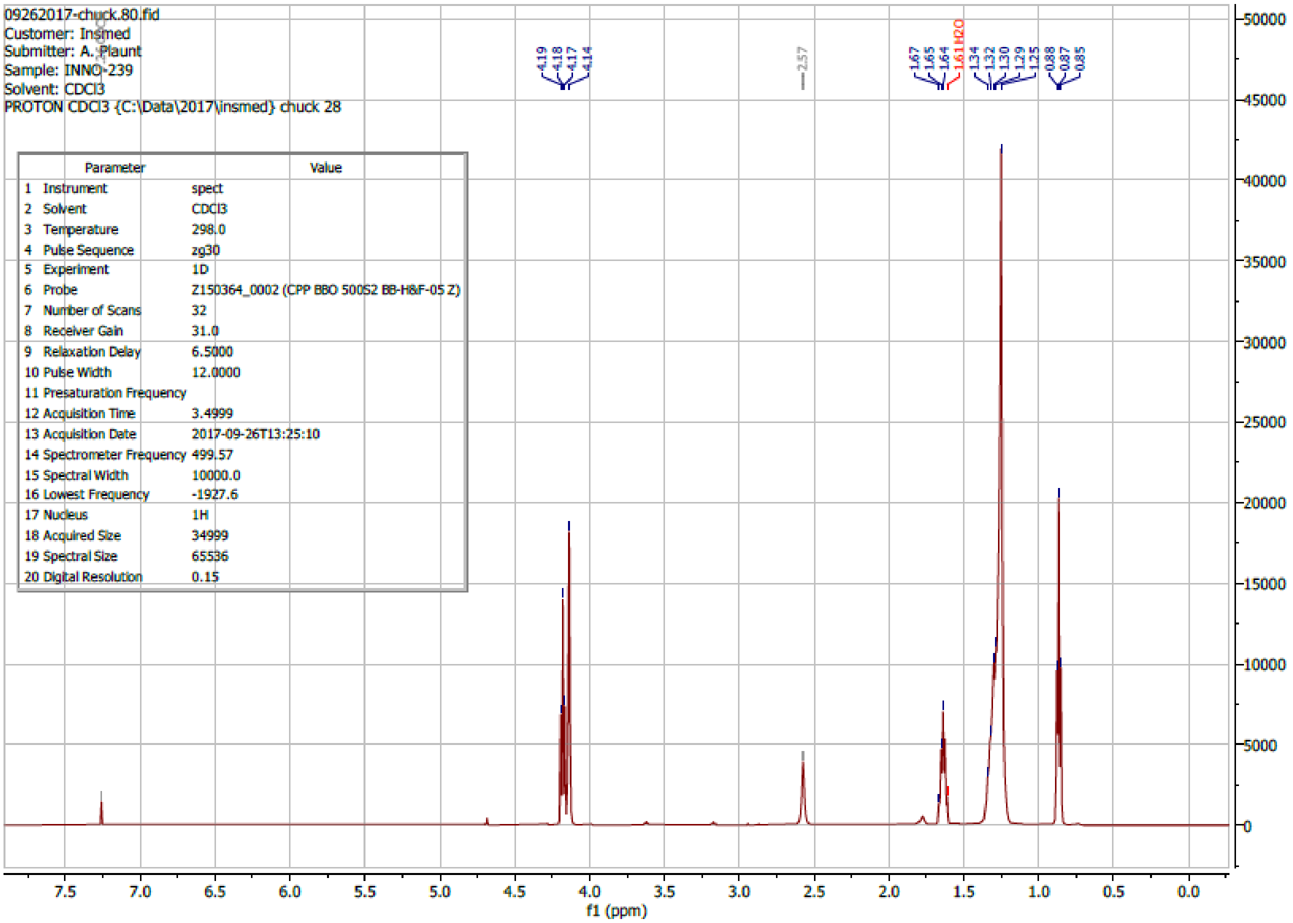

627

628 **Figure S7: 1H NMR of Decyl 2-Hydroxyacetate**

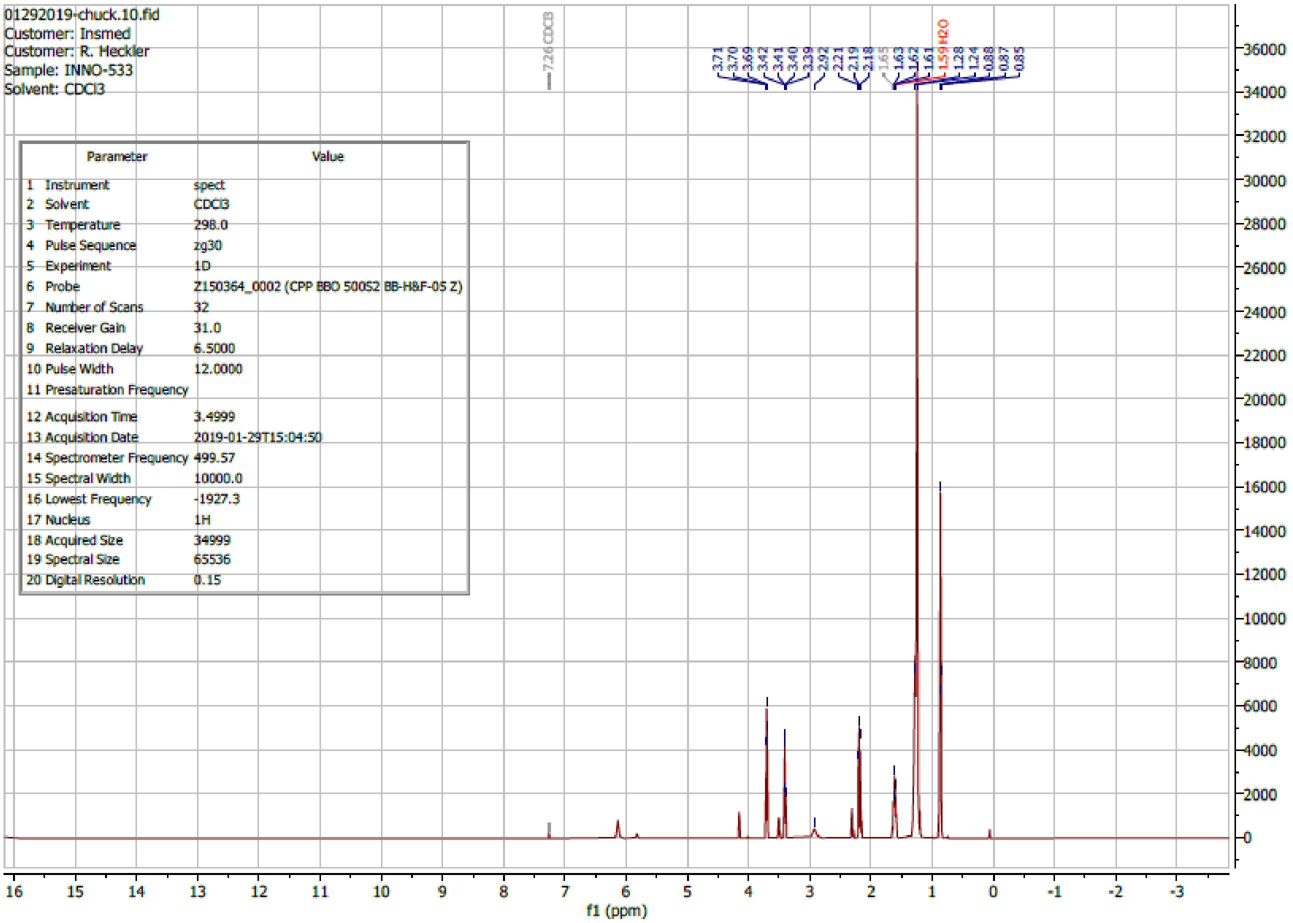

629

630 **Figure S8: 1H NMR of N-(2-Hydroxyethyl)decanamide**

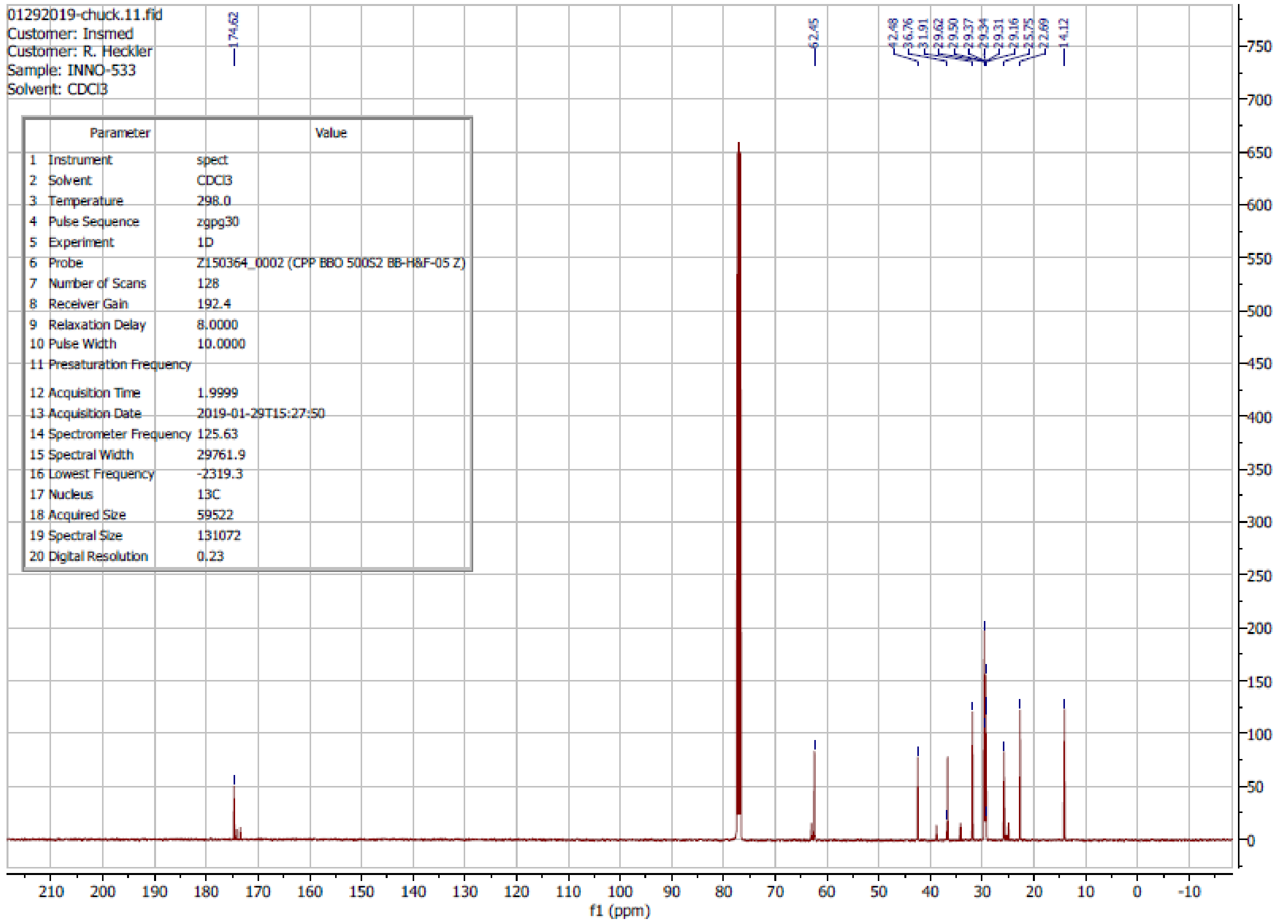

Figure S9: 13C NMR of N-(2-Hydroxyethyl)decanamide

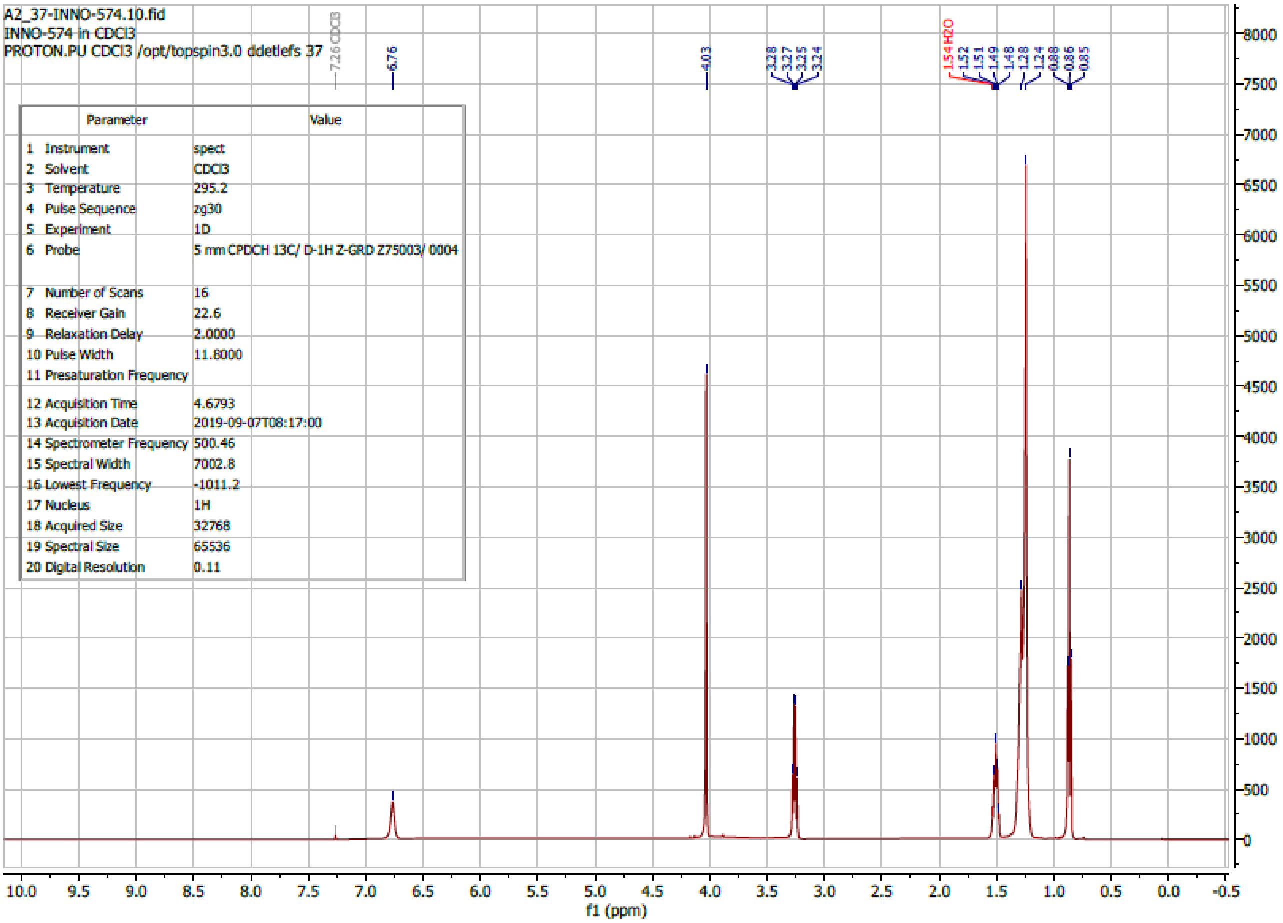

Figure S10: 1H NMR of N-Decyl-2-Hydroxyacetamide

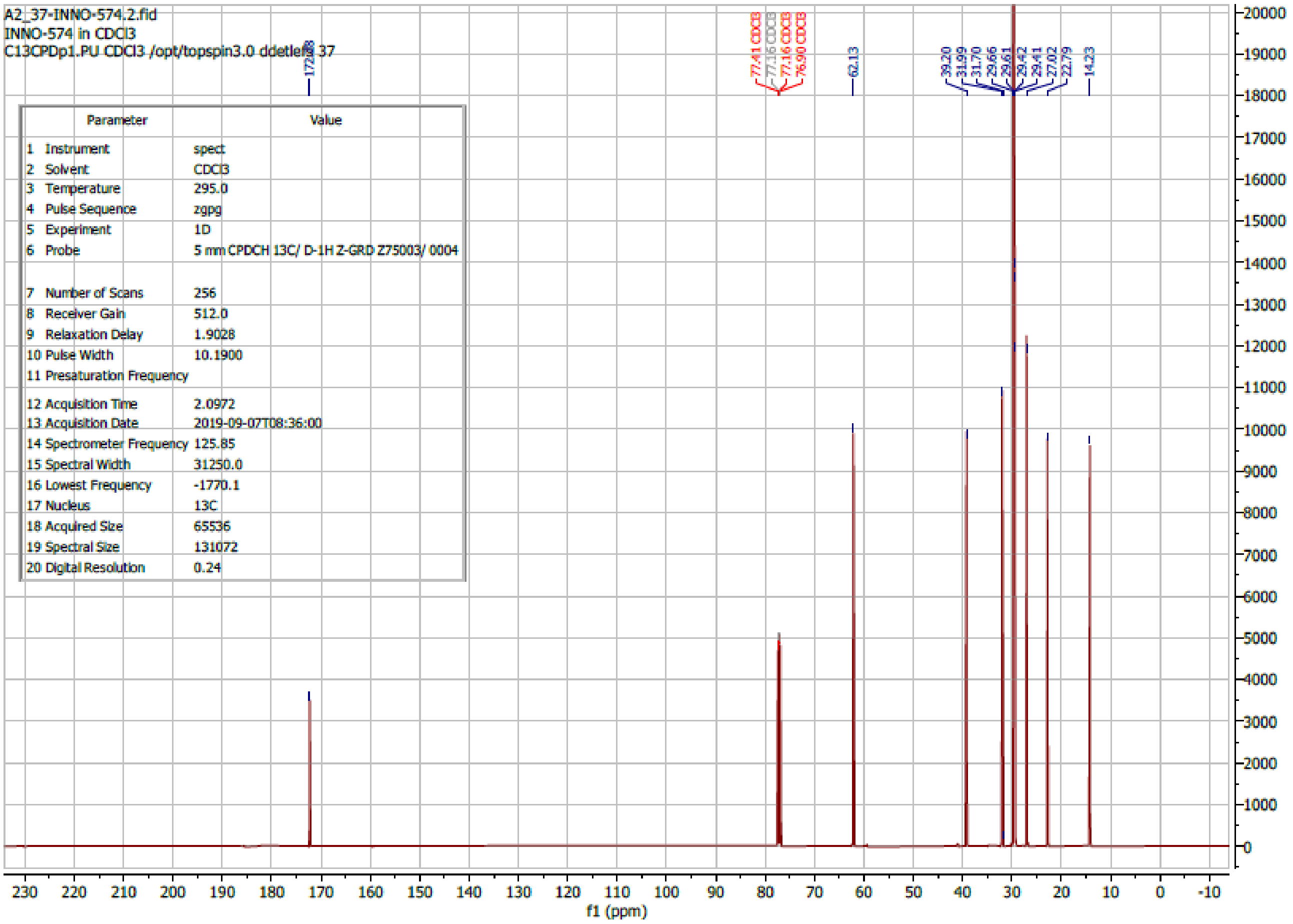

635

636 **Figure S11: 13C NMR of N-Decyl-2-Hydroxyacetamide**

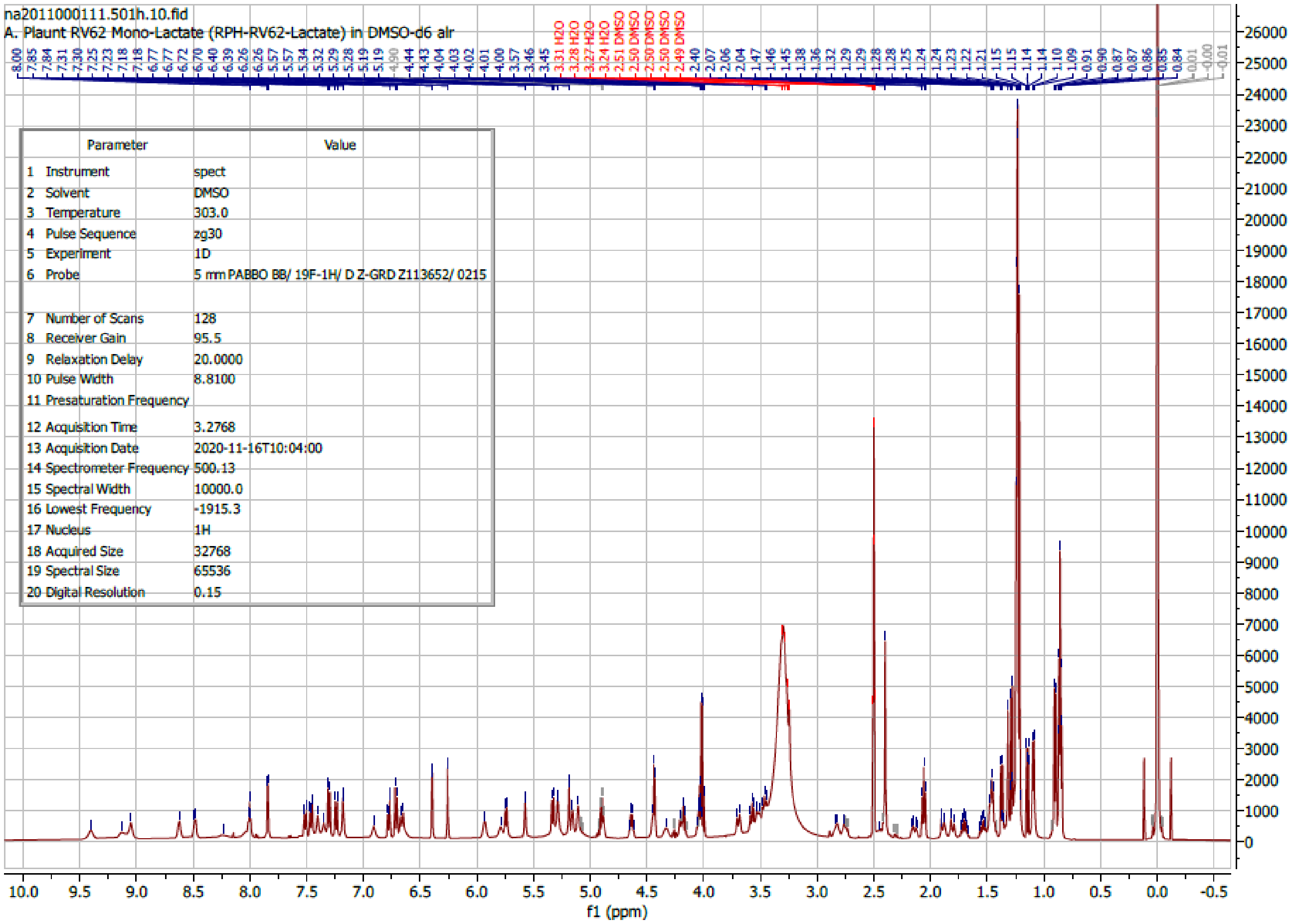

Figure S12: 1H NMR of RV62

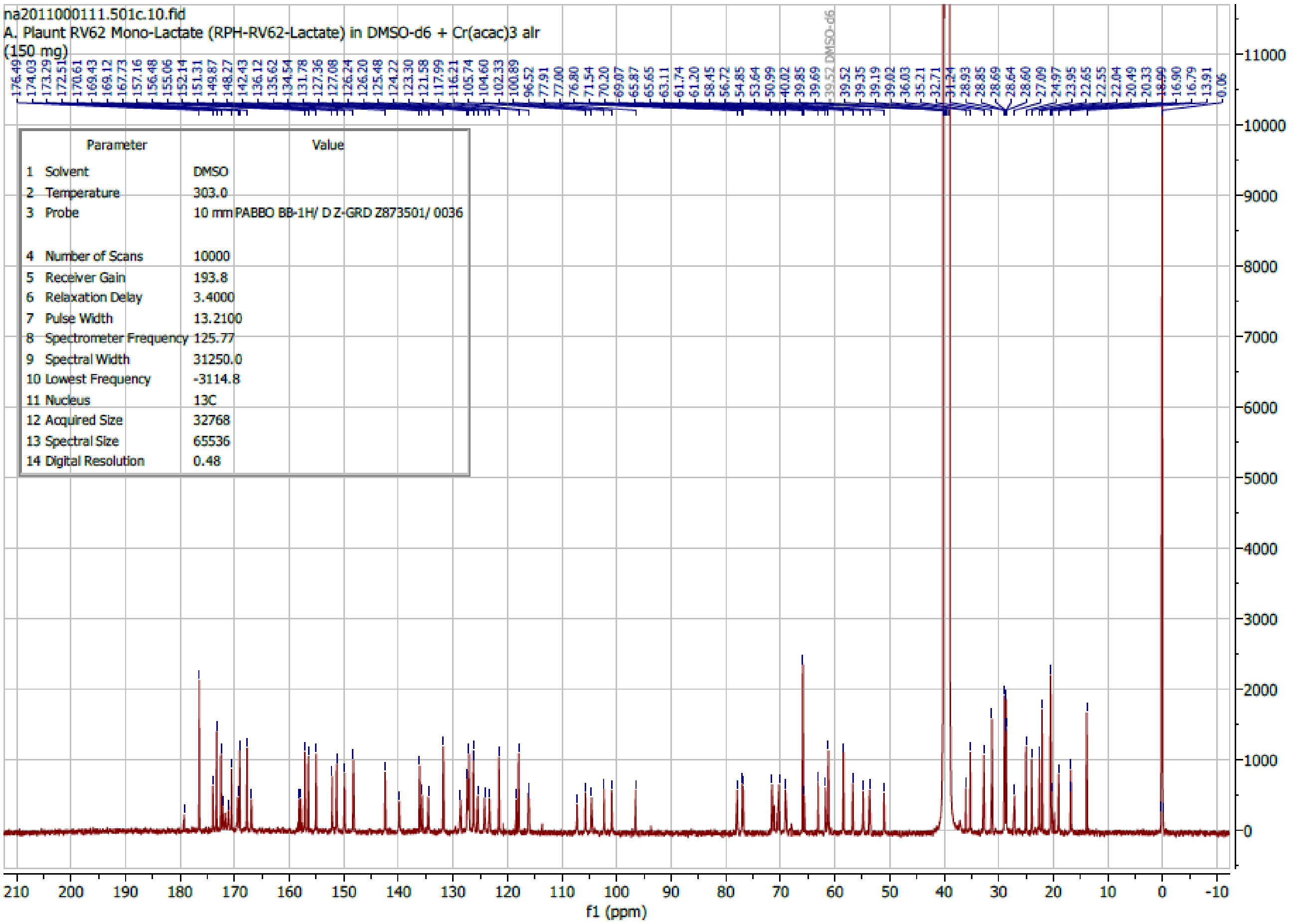

Figure S13: 13C NMR of RV62

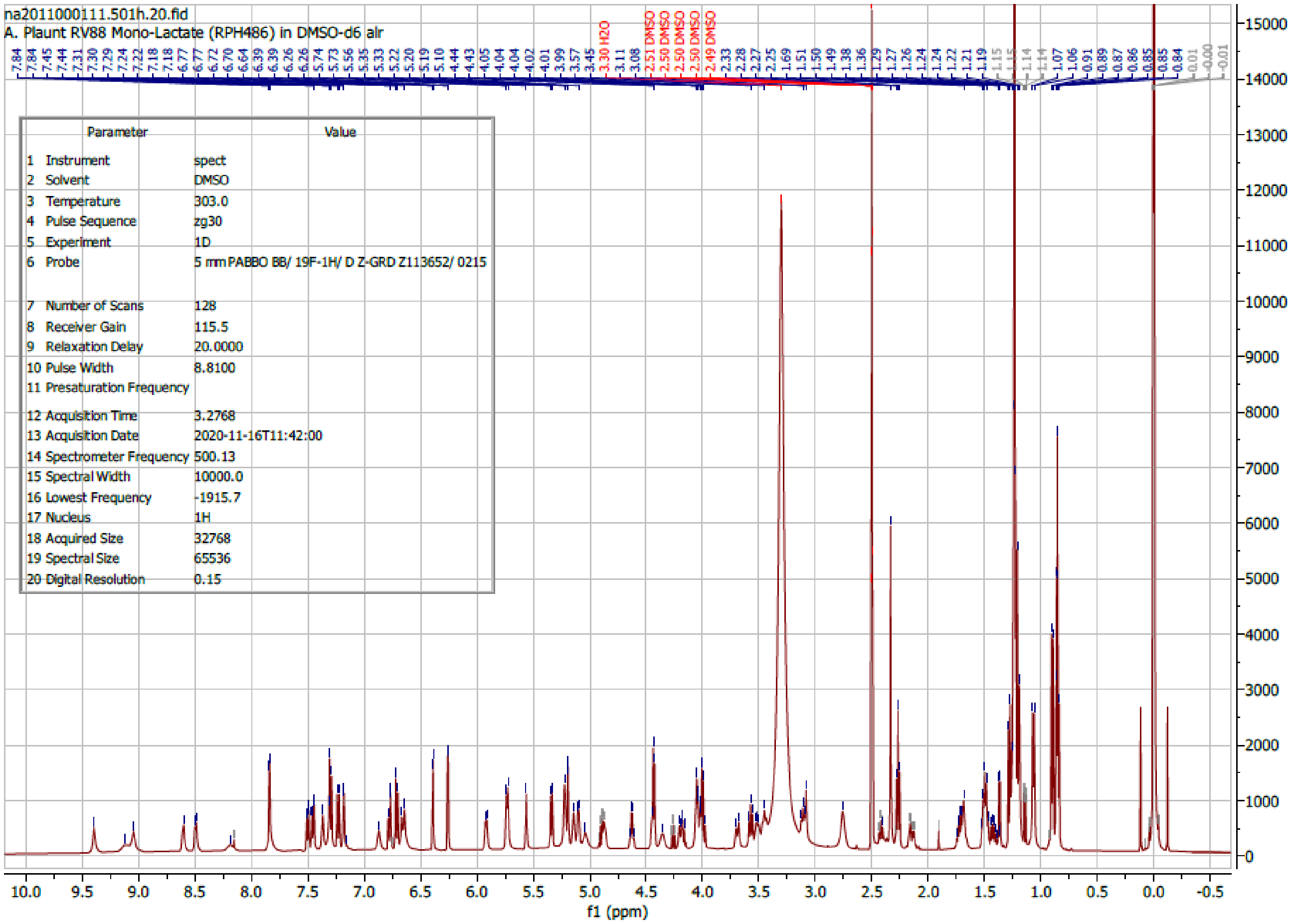

Figure S14: 1H NMR of RV88

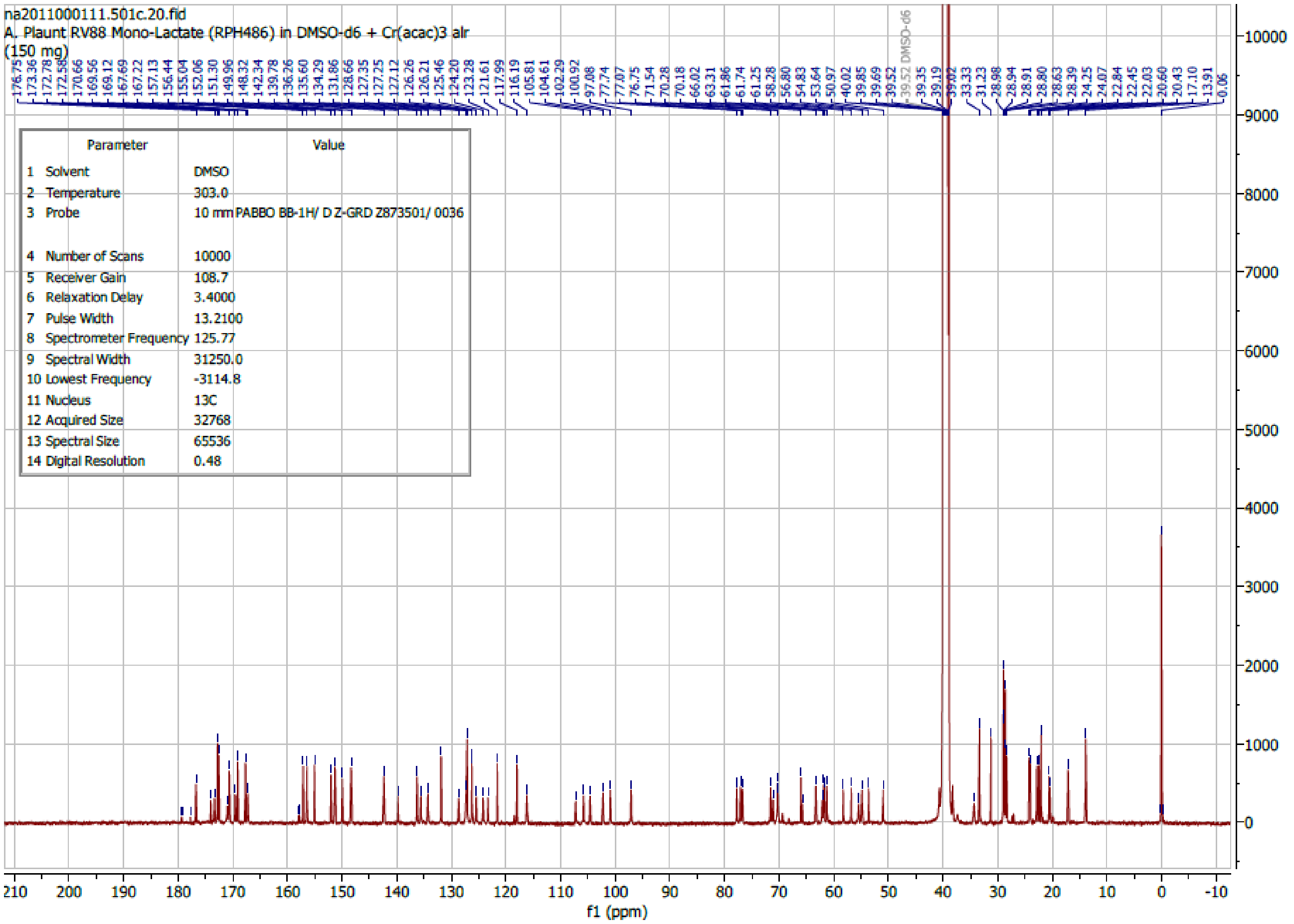

Figure S15: 13C NMR of RV88

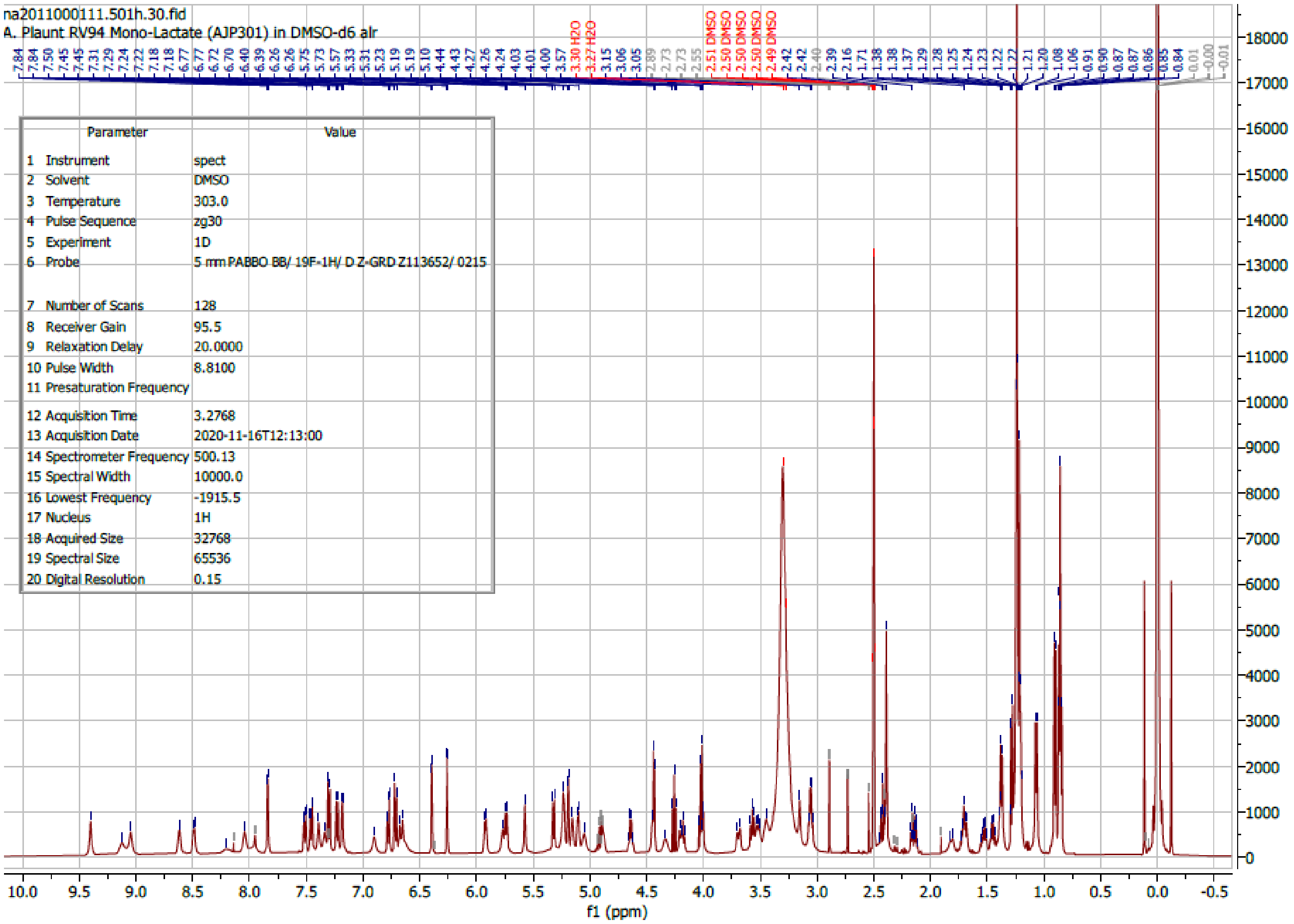

Figure S16: 1H NMR of RV94

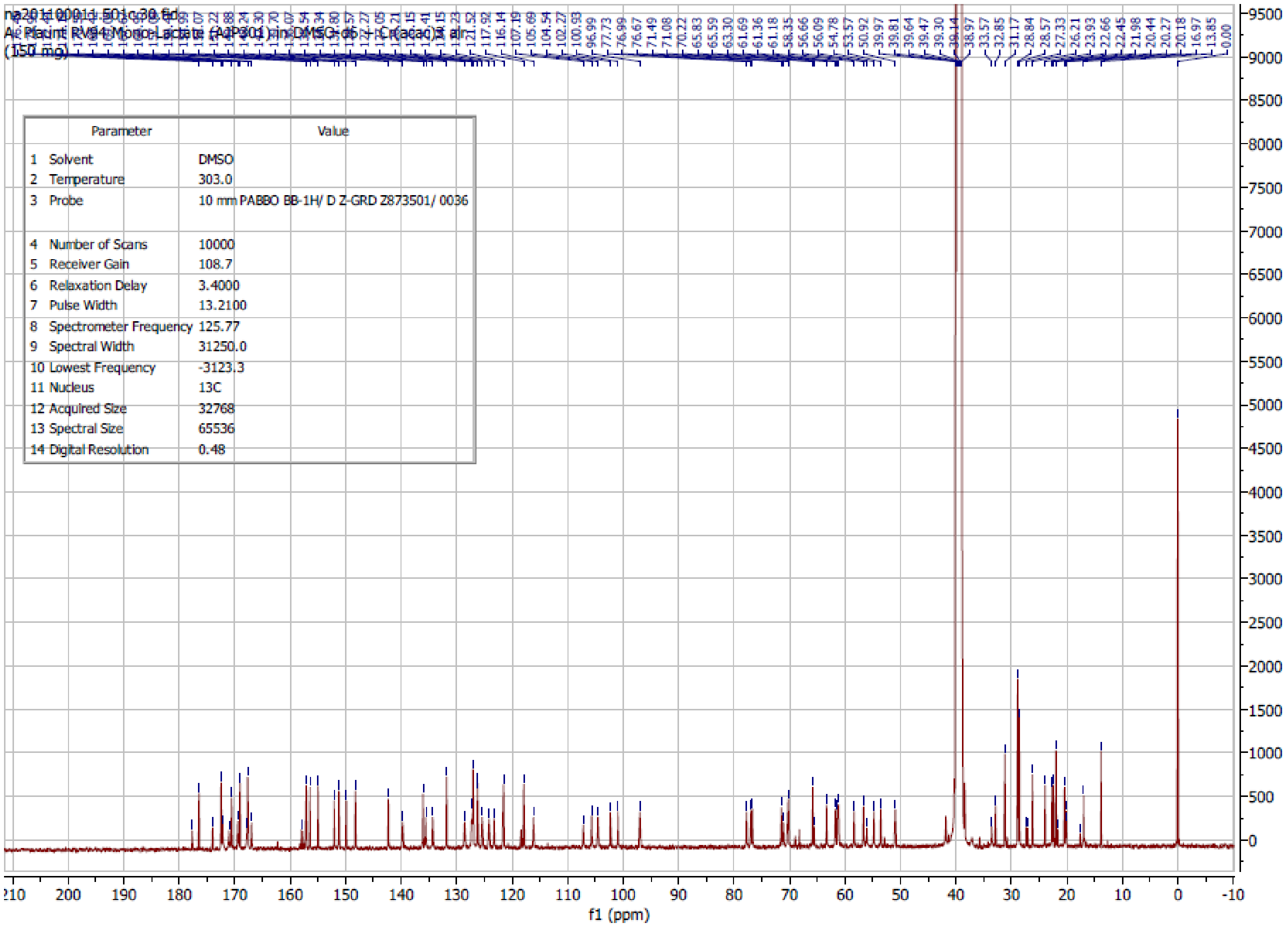

Figure S17: 13C NMR of RV94

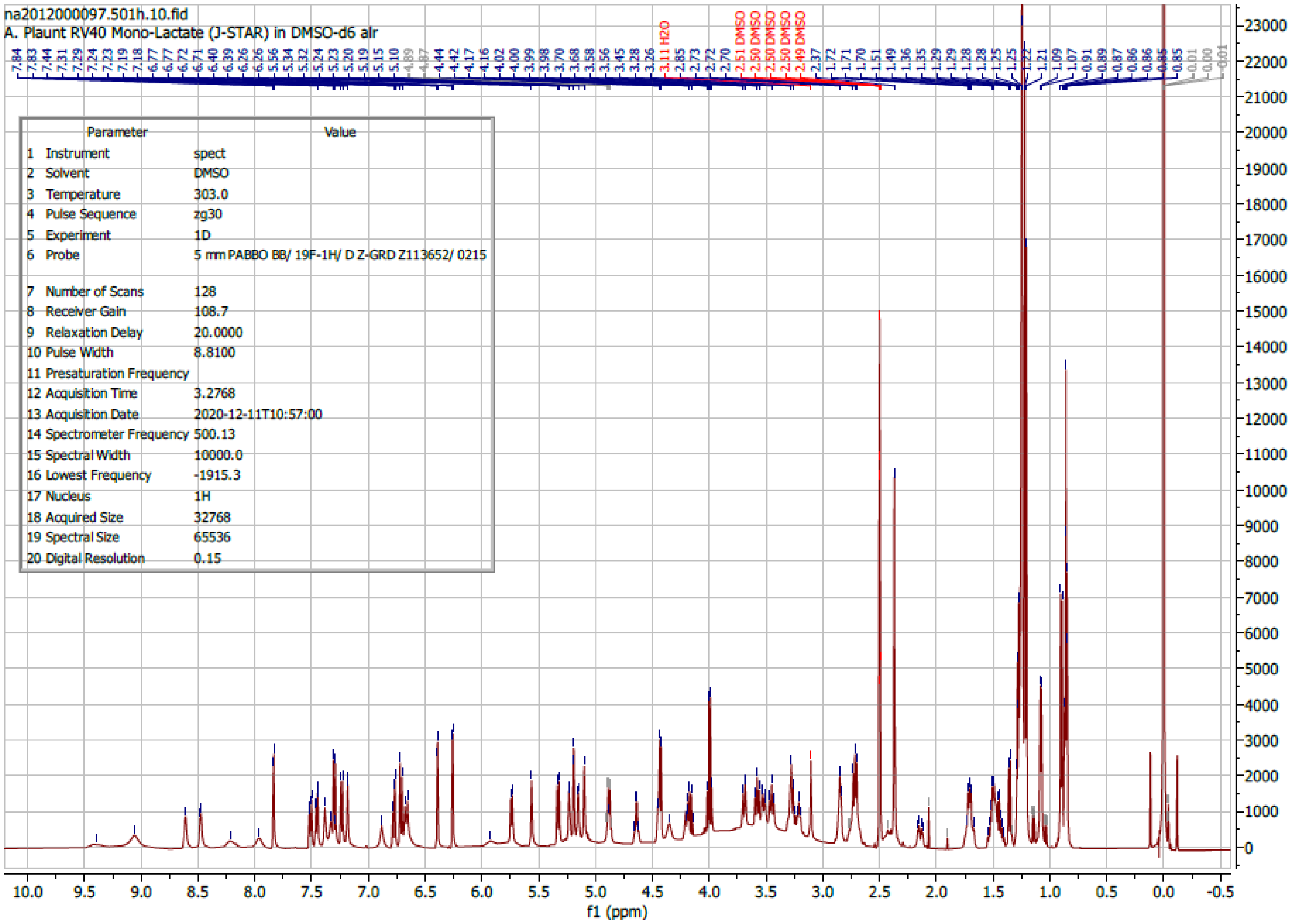

Figure S18: 1H NMR of RV40

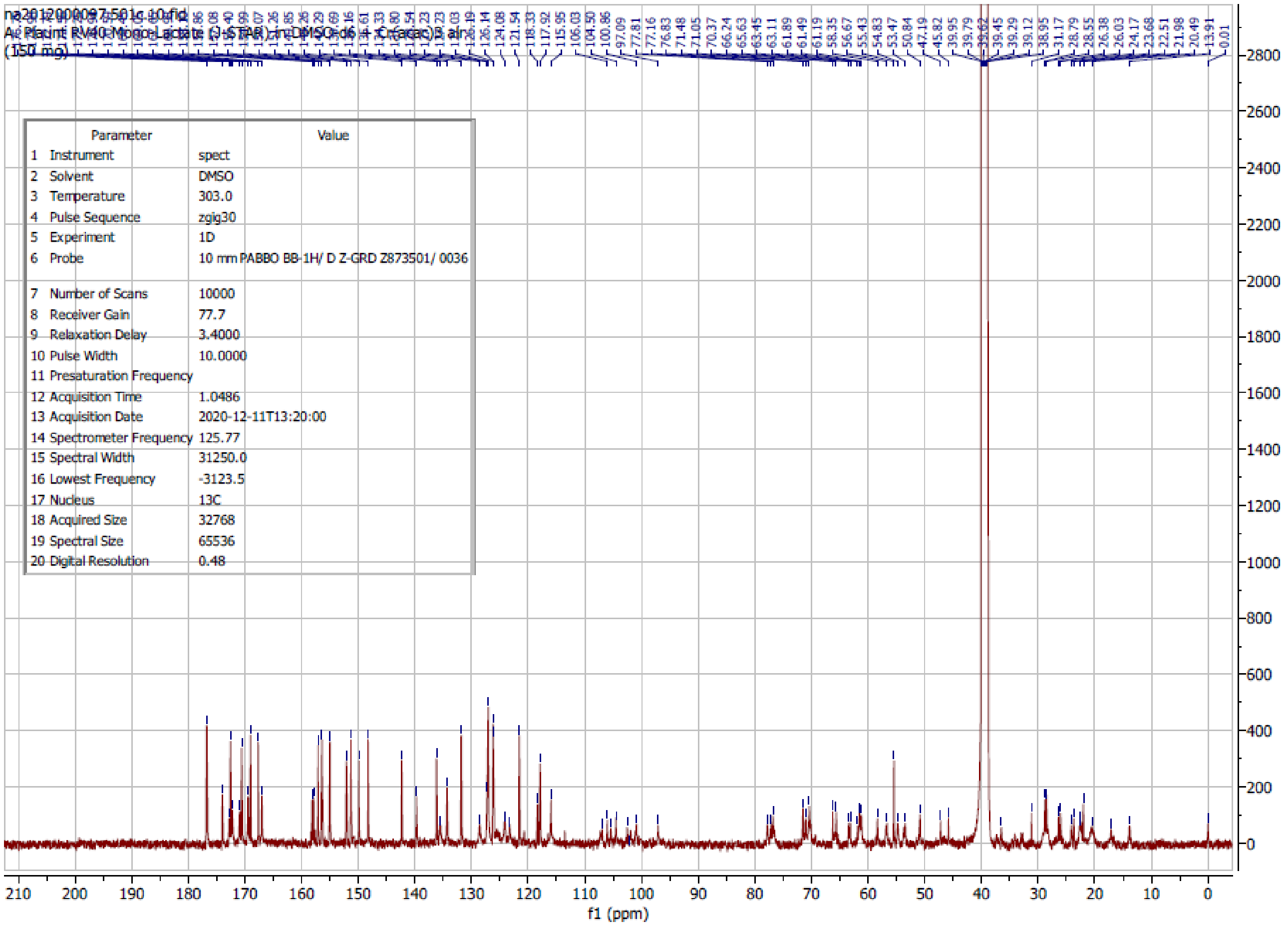

Figure S19: 13C NMR of RV40

MS DATA

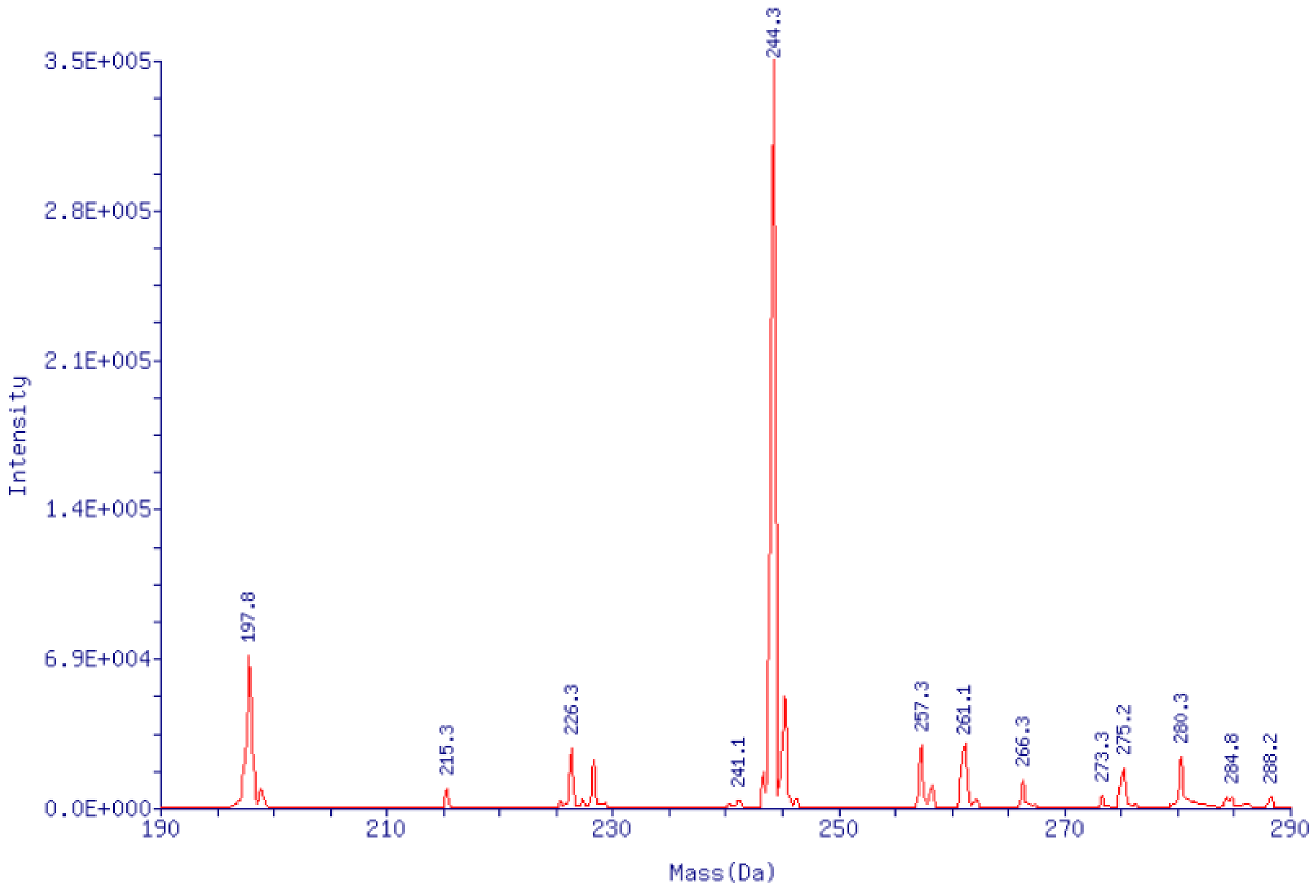

Figure S20: MS Data for 2-Hydroxyethyl Dodecanoate

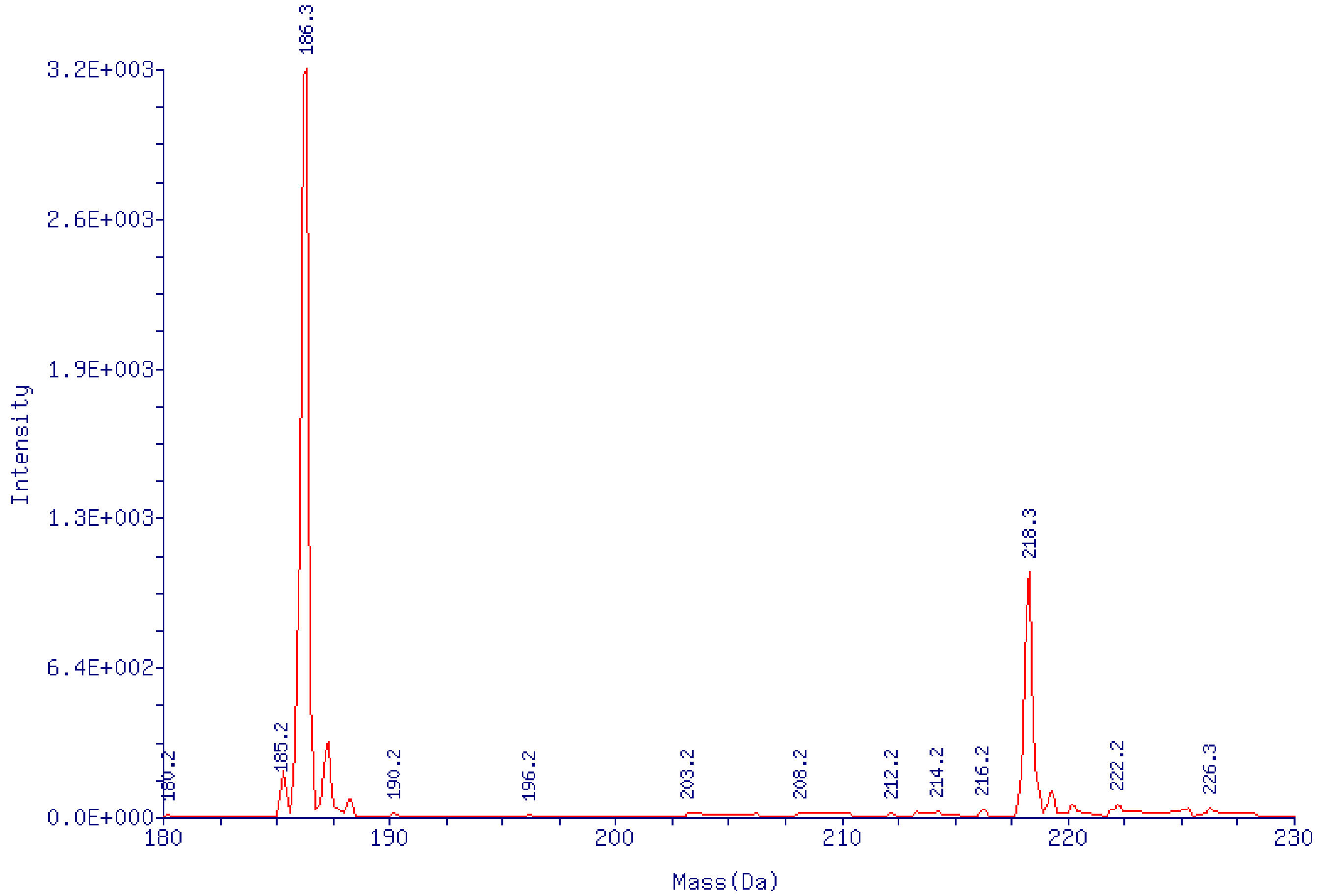

Figure S21: MS Data for Decyl 2-Hydroxyacetate

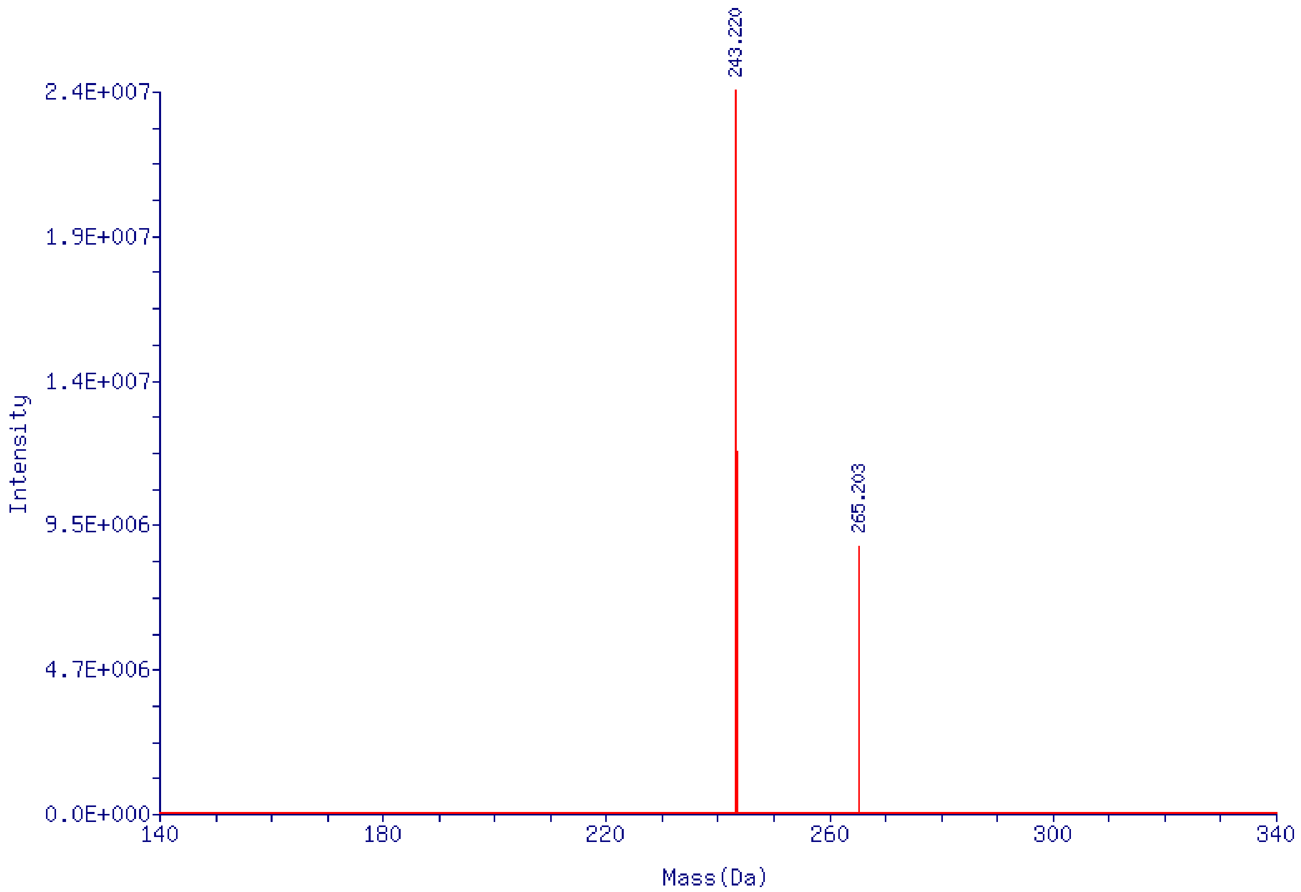

Figure S22: MS Data for N-(2-Hydroxyethyl)decanamide

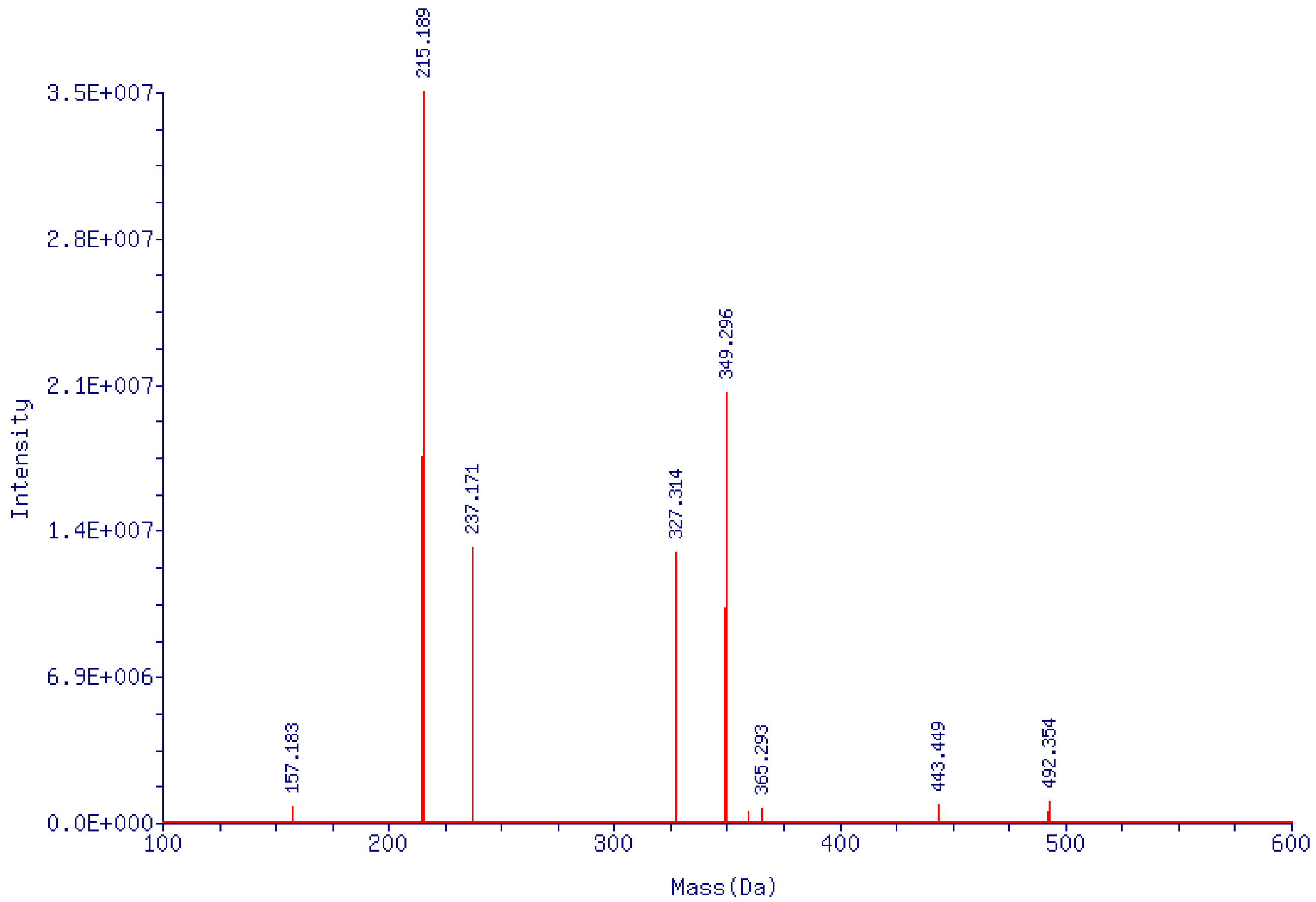

660

661 **Figure S23: MS Data for N-Decyl-2-Hydroxyacetamide**

HPLC & LCMS CHROMATOGRAMS

RV40: **HPLC**: Analytical HPLC trace of RV40 showing >98% purity. **LCMS**: Method: 2.1 x 100 mm Halo Peptide ES-C18, 2.7 µm, 2-40% in 20 min after 1 min hold, 0.4 mL/min, 50 °C, A = 0.05% TFA in H_2O, B = 0.05% TFA in ACN. LC-MS (ESI$^+$) calculated for $C_{78}H_{100}Cl_2N_{10}O_{24}$ $[M+H]^+$ *m/z*: 1630.6289; found: 1630.630; retention time = 17.9 minutes.

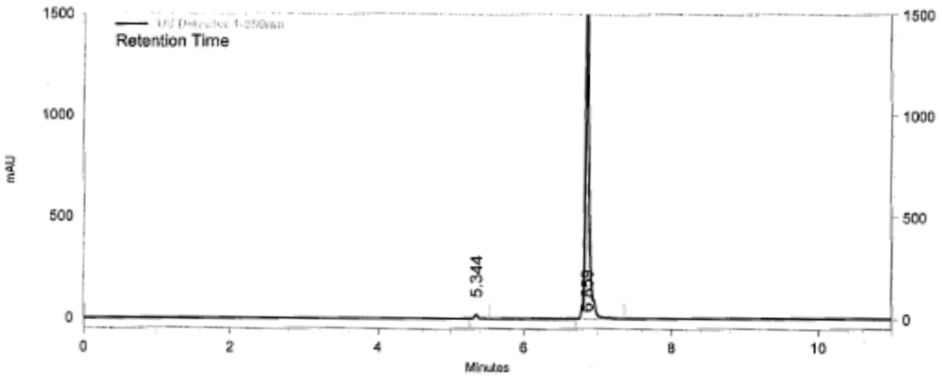

UV Detector 1-280nm Results
(System (10/23/2017 6:08:52 PM) (Original))

Pk #	Retention Time	Area	Area Percent	Integration Codes
1	5.344	59067	1.067	BB
2	6.859	5478688	98.933	BB

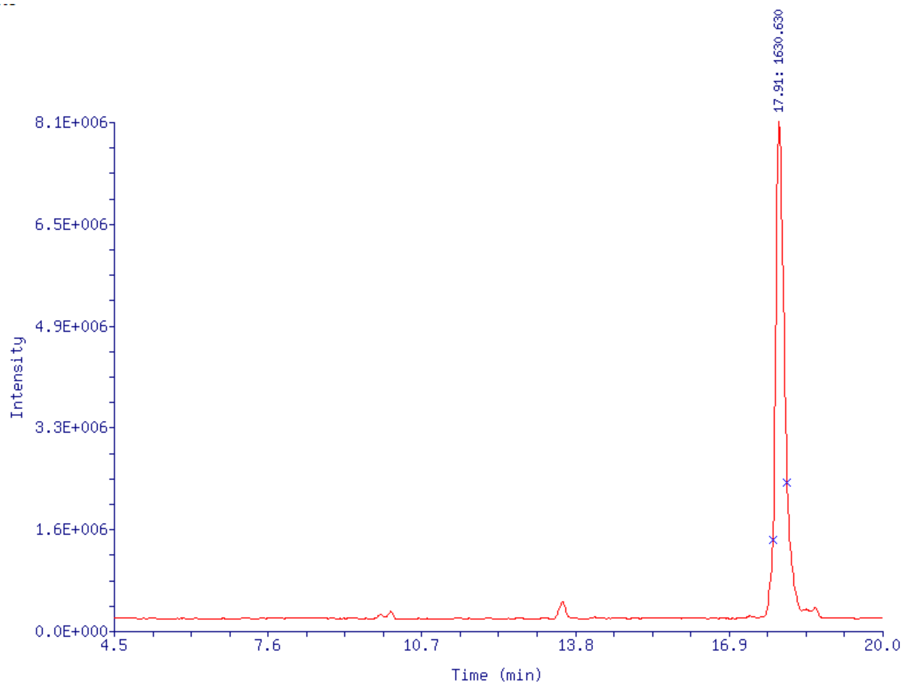

Figure S24: HPLC (top) and LCMS (bottom) Chromatograms for RV40

Telavancin: **HPLC**: Analytical HPLC trace of TLV showing >98% purity. **LCMS**: Method: 2.1 x 100 mm Halo Peptide ES-C18, 2.7 µm, 2-40% in 20 min after 1 min hold, 0.4 mL/min, 50 °C, A = 0.05% TFA in H_2O, B = 0.05% TFA in ACN. LC-MS (ESI$^+$) calculated for $C_{80}H_{106}Cl_2N_{11}O_{27}P$ $[M+H]^+$ *m/z*: 1753.6374; found: 1753.637; retention time = 13.3 minutes.

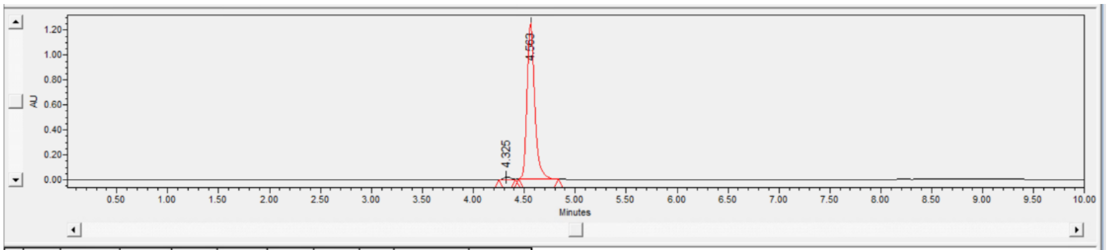

	Name	Retention Time (min)	Area (µV*sec)	% Area	Height (µV)	Int Type	Amount	Units	Peak Type	Peak Codes
1		4.325	98483	1.52	22235	bb			Unknown	
2		4.563	6385879	98.48	1241368	bb			Unknown	

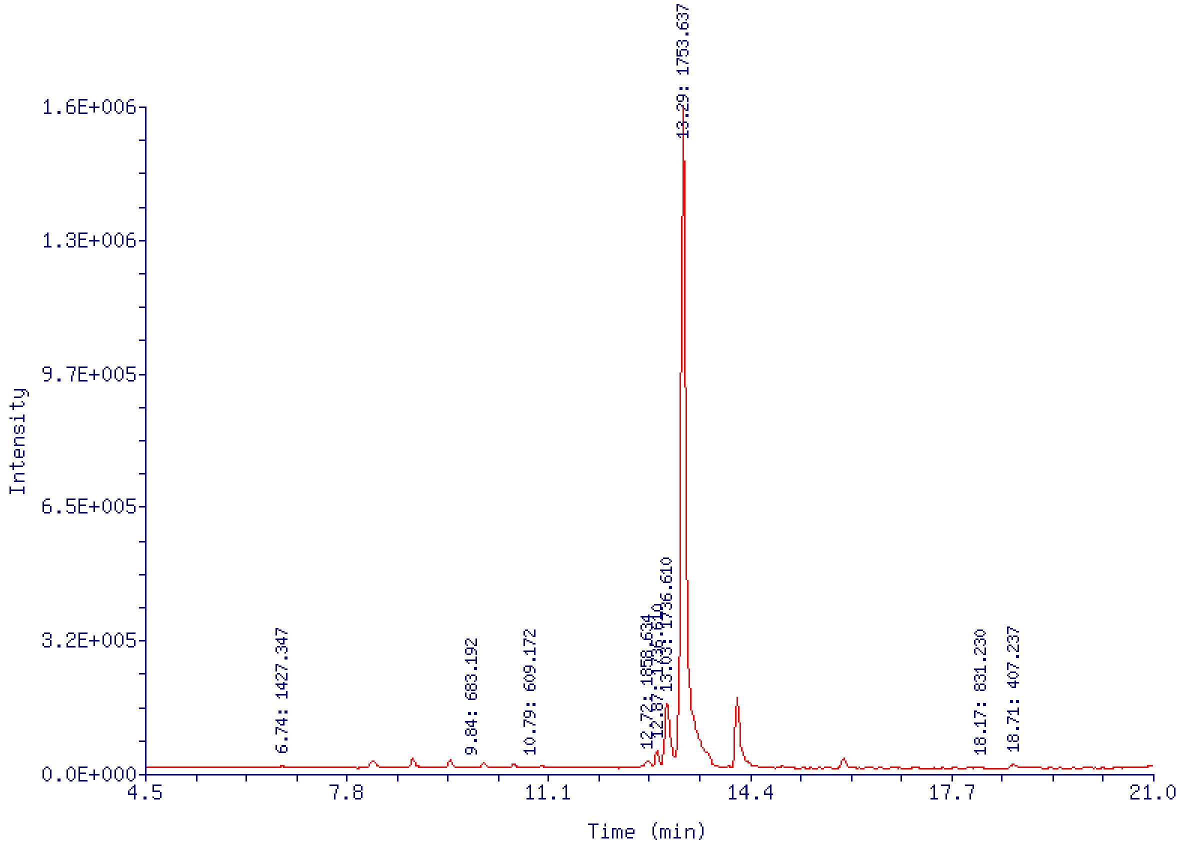

Figure S25: HPLC (top) and LCMS (bottom) Chromatograms for TLV

<u>RV90</u>: HPLC: Analytical HPLC trace of RV90 showing >98% purity. **LCMS**: Method: 2.1 x 100 mm Halo Peptide ES-C18, 2.7 µm, 2-40% in 20 min after 1 min hold, 0.4 mL/min, 50 °C, A = 0.05% TFA in H_2O, B = 0.05% TFA in ACN. LC-MS (ESI^+) calculated for $C_{76}H_{93}Cl_2N_9O_{26}$ $[M+H]^+$ *m/z*: 1617.5609; found: 1617.561; retention time = 15.2-15.6 minutes.

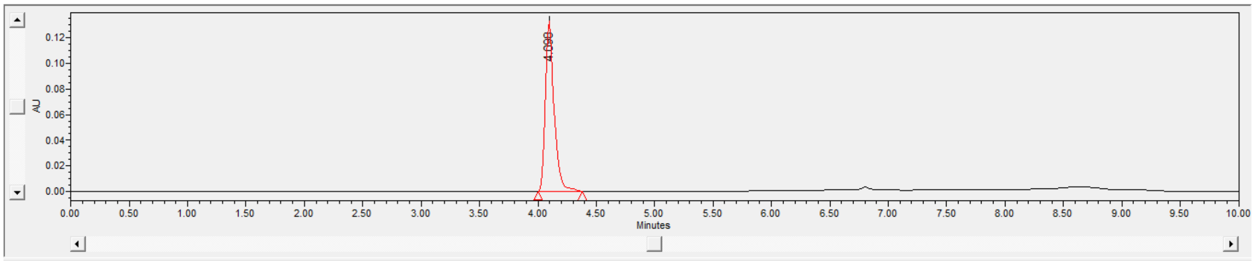

	Name	Retention Time (min)	Area (µV*sec)	% Area	Height (µV)	Int Type	Amount	Units	Peak Type	Peak Codes
1		4.098	692482	100.00	130307	bb			Unknown	

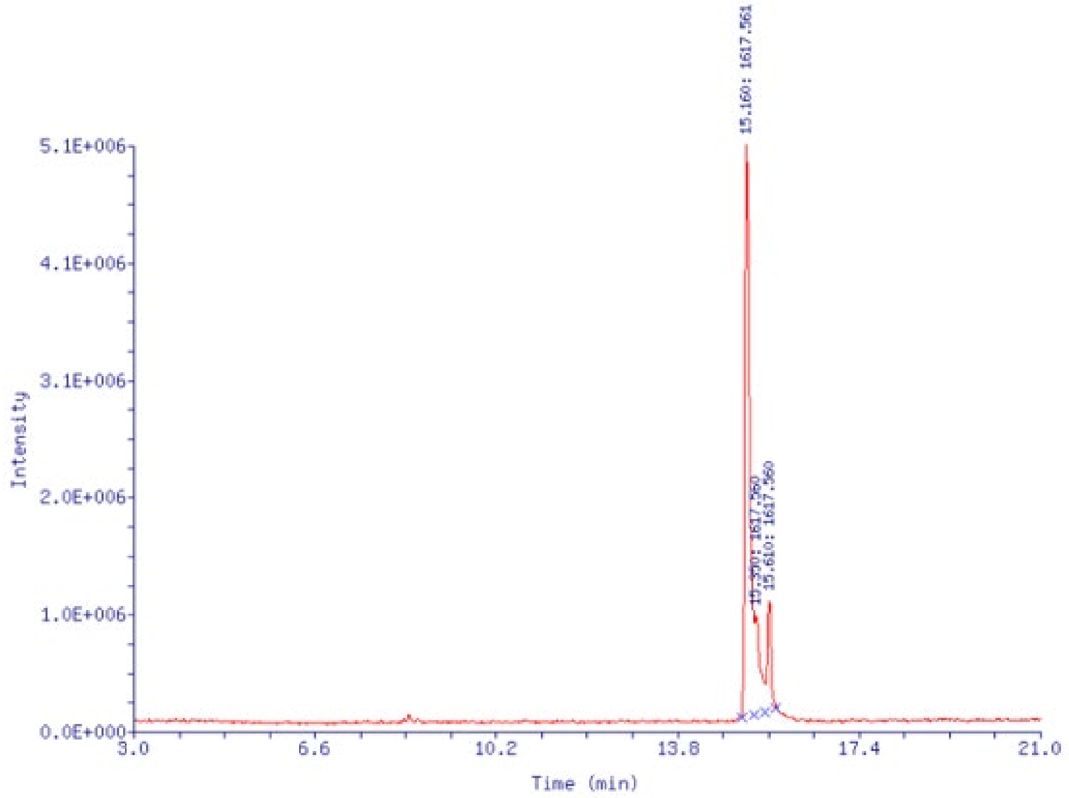

Figure S26: HPLC (top) and LCMS (bottom) Chromatograms for RV90

RV54: **HPLC**: Analytical HPLC trace of RV54 showing >98% purity. **LCMS**: Method: 2.1 x 100 mm Halo Peptide ES-C18, 2.7 µm, 2-40% in 20 min after 1 min hold, 0.4 mL/min, 50 °C, A = 0.05% TFA in H_2O, B = 0.05% TFA in ACN. LC-MS (ESI^+) calculated for $C_{78}H_{97}Cl_2N_9O_{26}$ $[M+H]^+$ *m/z*: 1645.9522; found: 1645.592; retention time = 15.7-16.1 minutes.

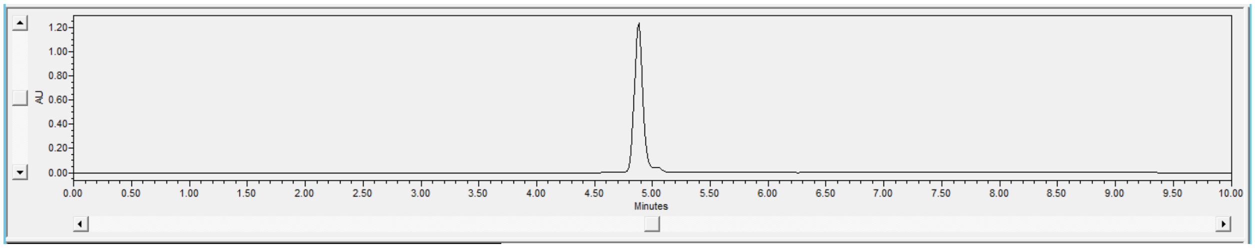

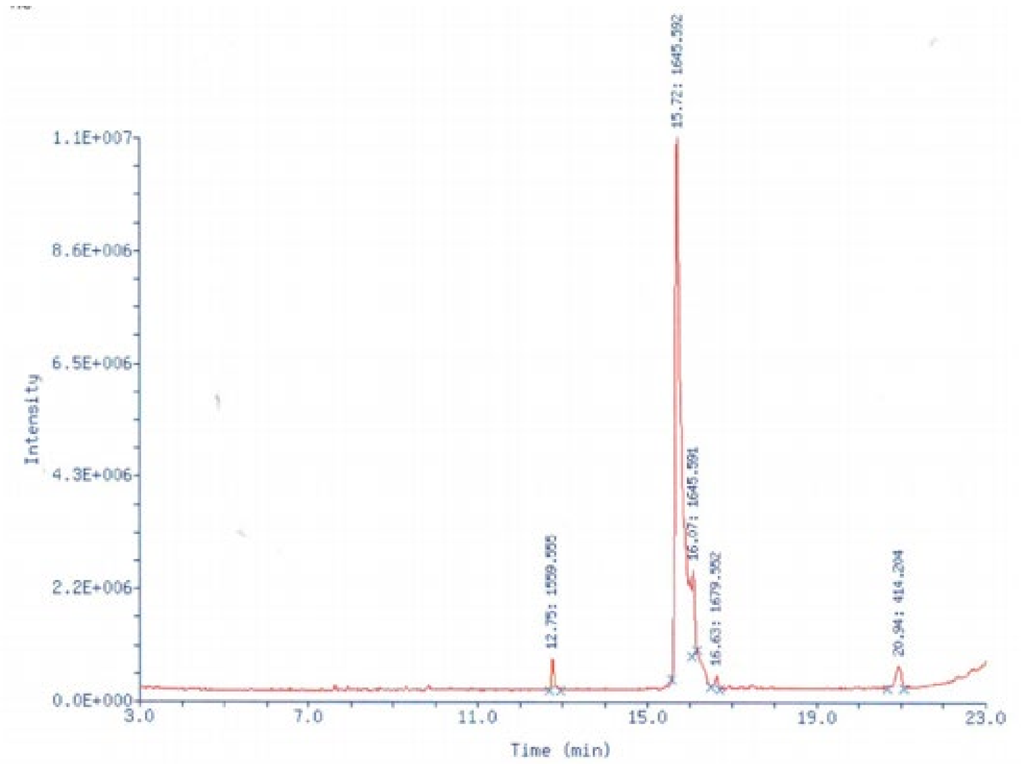

Figure S27: HPLC (top) and LCMS (bottom) Chromatograms for RV54

RV65: **HPLC**: Analytical HPLC trace of RV65 showing >98% purity. **LCMS:** Method: 2.1 x 100 mm Halo Peptide ES-C18, 2.7 µm, 2-40% in 20 min after 1 min hold, 0.4 mL/min, 50 °C, A = 0.05% TFA in H_2O, B = 0.05% TFA in ACN. LC-MS (ESI$^+$) calculated for $C_{80}H_{101}Cl_2N_9O_{24}$ $[M+H]^+$ *m/z*: 1673.6235; found: 1673.623; retention time = 19.6-18.9 minutes.

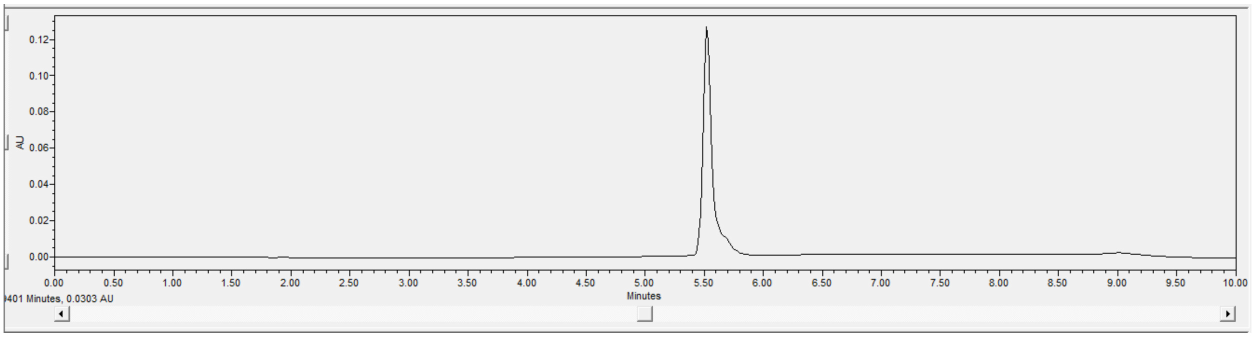

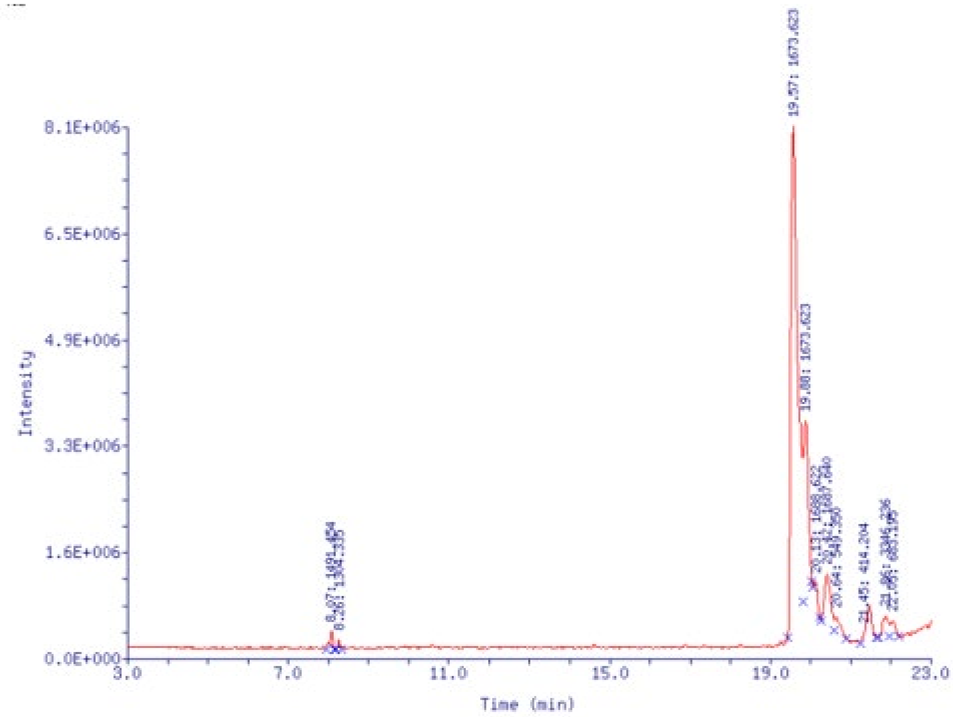

Figure S28: HPLC (top) and LCMS (bottom) Chromatograms for RV65

RV88: **HPLC**: Analytical HPLC trace of RV88 showing >98% purity. **LCMS:** Method: 2.1 x 100 mm Halo Peptide ES-C18, 2.7 µm, 2-40% in 20 min after 1 min hold, 0.4 mL/min, 50 °C, A = 0.05% TFA in H_2O, B = 0.05% TFA in ACN. LC-MS (ESI^+) calculated for $C_{82}H_{105}Cl_2N_9O_{26}$ $[M+H]^+$ *m/z*: 1701.6548; found: 1701.655; retention time = 22.1minutes.

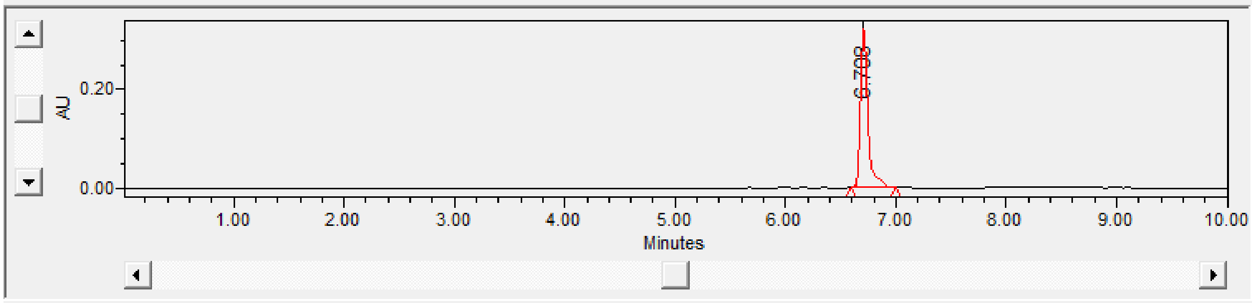

	Name	Retention Time (min)	Purity1 Angle	Purity1 Threshold	PDA/FLR Match1 Spect. Name	PDA/FLR Match1 Angle	PDA/FLR Match1 Threshold	PDA/FLR Match1 Lib. Name	Area (µV*sec)	% Area	Heig (µV
1		6.708							1642989	100.00	3227

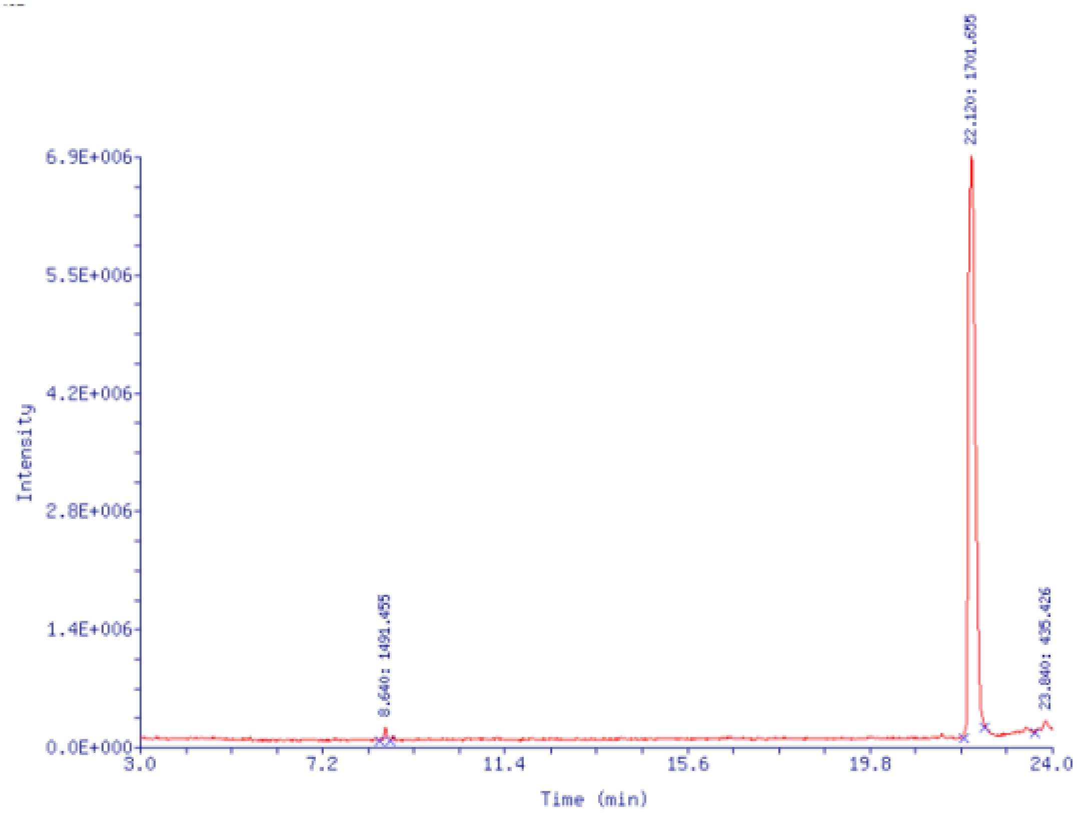

Figure S29: HPLC (top) and LCMS (bottom) Chromatograms for RV88

RV89: **HPLC**: Analytical HPLC trace of RV89 showing >98% purity. **LCMS:** Method: 2.1 x 100 mm Halo Peptide ES-C18, 2.7 µm, 2-40% in 20 min after 1 min hold, 0.4 mL/min, 50 °C, A = 0.05% TFA in H_2O, B = 0.05% TFA in ACN. LC-MS (ESI$^+$) calculated for $C_{84}H_{109}Cl_2N_9O_{26}$ $[M+H]^+$ *m/z*: 1729.6861; found: 1729.686; retention time = 22.7 minutes.

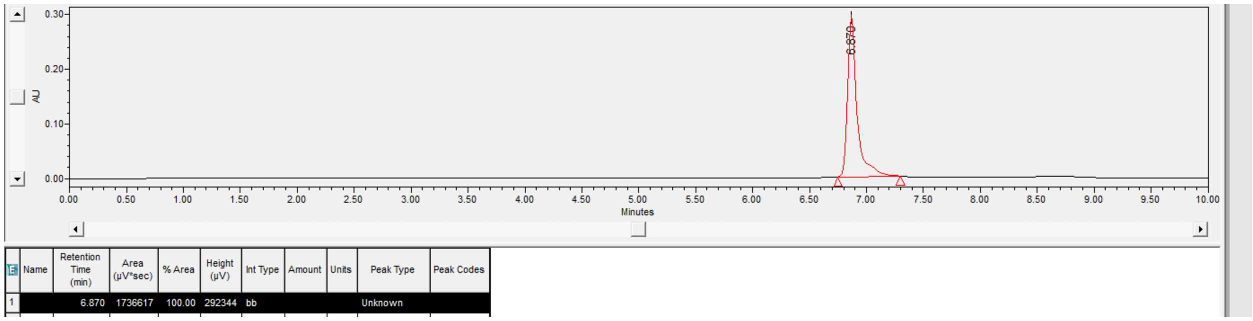

	Name	Retention Time (min)	Area (µV*sec)	% Area	Height (µV)	Int Type	Amount	Units	Peak Type	Peak Codes
1		6.870	1736617	100.00	292344	bb			Unknown	

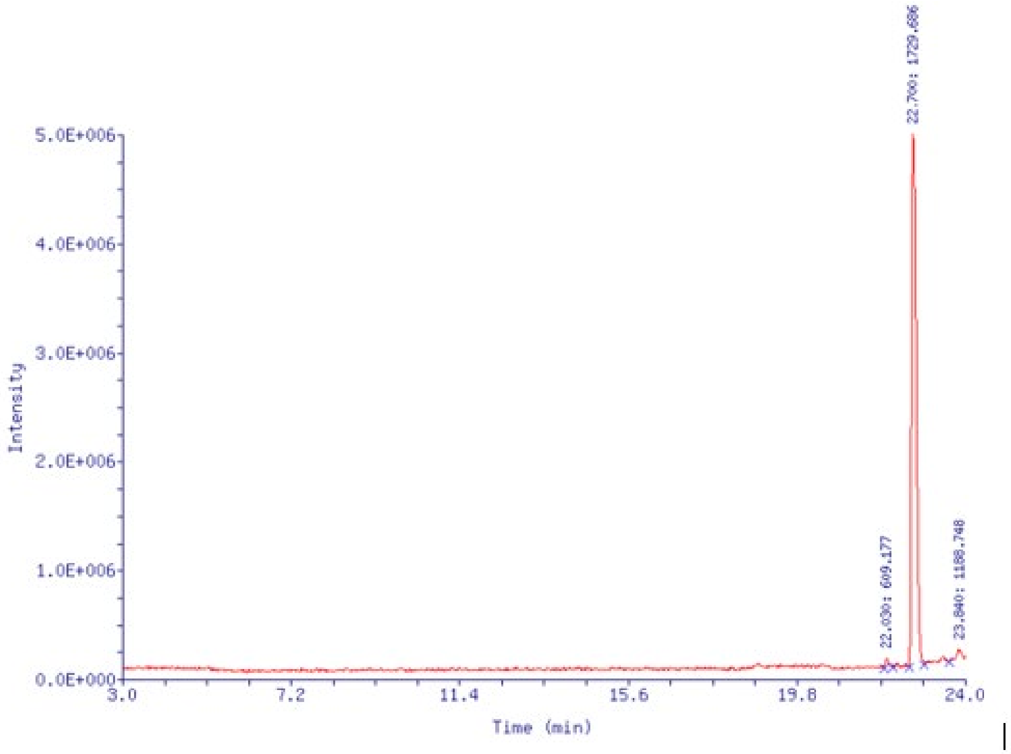

Figure S30: HPLC (top) and LCMS (bottom) Chromatograms for RV89

<u>RV80</u>: **HPLC**: Analytical HPLC trace of RV showing >98% purity. **LCMS:** Method: 2.1 x 100 mm Halo Peptide ES-C18, 2.7 µm, 2-40% in 20 min after 1 min hold, 0.4 mL/min, 50 °C, A = 0.05% TFA in H_2O, B = 0.05% TFA in ACN. LC-MS (ESI$^+$) calculated for $C_{68}H_{79}Cl_2N_9O_{25}$ $[M+H]^+$ *m/z*: 1491.4564; found: 1491.463; retention time = 8.1-8.9 minutes.

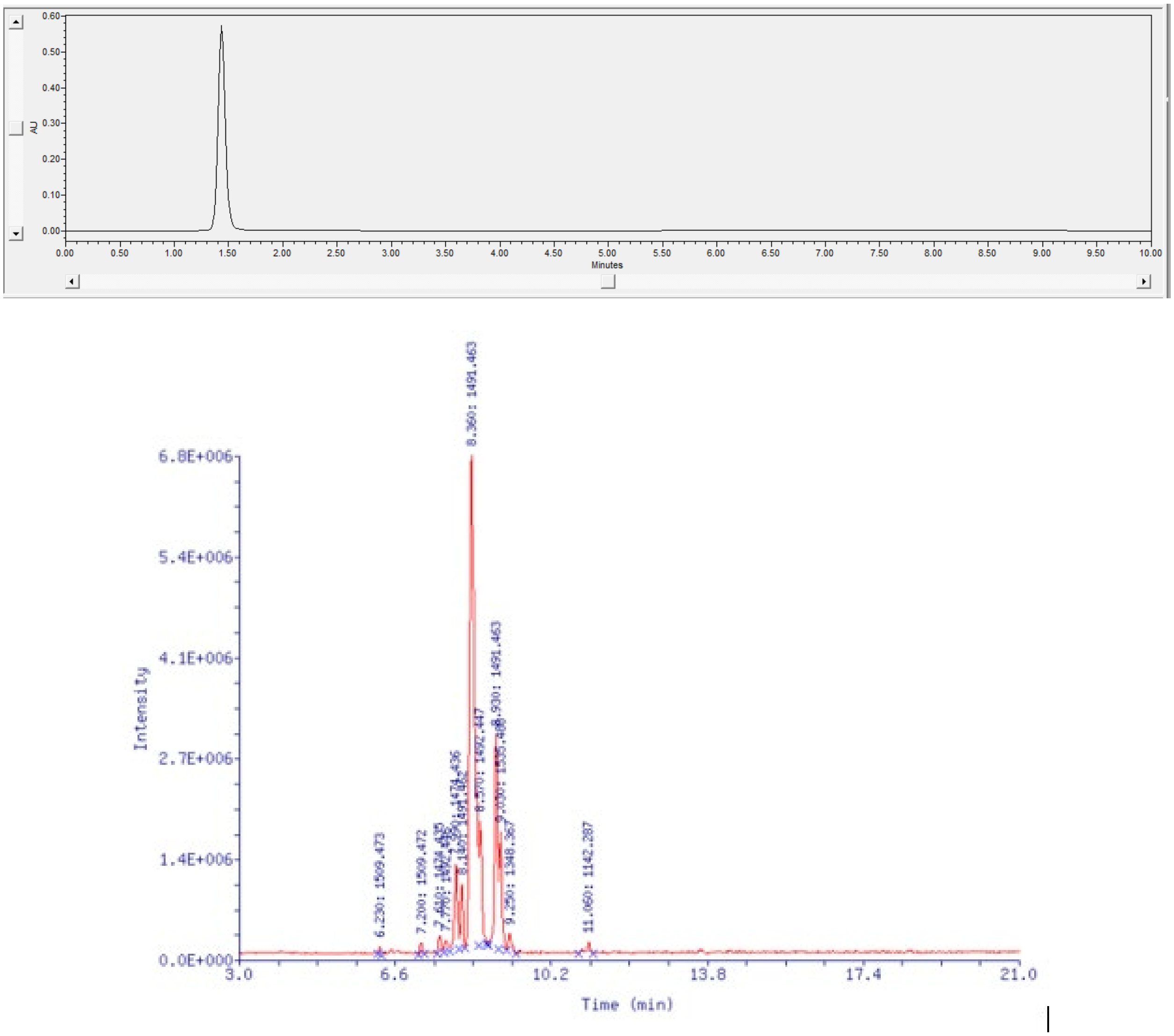

Figure S31: HPLC (top) and LCMS (bottom) Chromatograms for RV80

RV124: HPLC: Analytical HPLC trace of RV124 showing >98% purity. **LCMS:** Method: 2.1 x 100 mm Waters HILIC, 1.6 µm, 5-80% in 1.5 min after 1.5 min hold, 0.6 mL/min, 50 °C, A = 0.05% TFA in H_2O, B = 0.05% TFA in CAN; Waters QdA detector. LC-MS (ESI$^+$) calculated for $C_{76}H_{93}Cl_2N_9O_{26}$ $[M+2H]^{+2}$ *m/z*: 809.8 ; found: 810.6; retention time = 2.0 minutes.

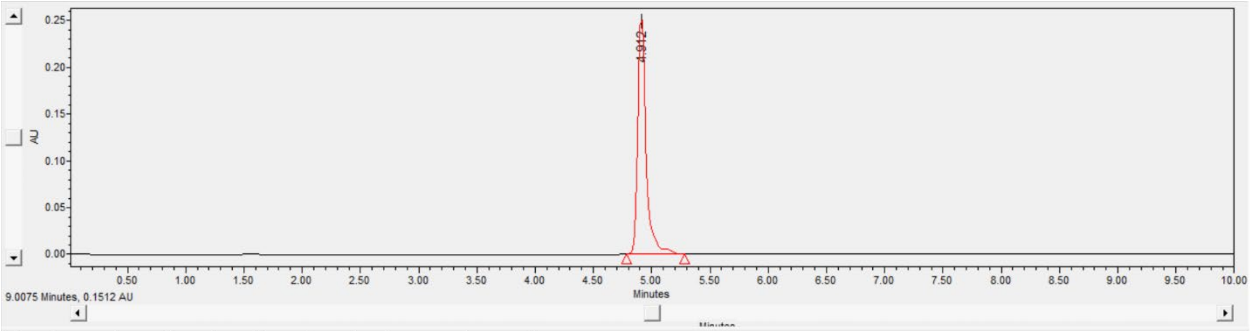

	Name	Retention Time (min)	Area (µV*sec)	% Area	Height (µV)	Int Type	Amount	Units	Peak Type	Peak Codes
1		4.912	1318038	100.00	249622	bb			Unknown	

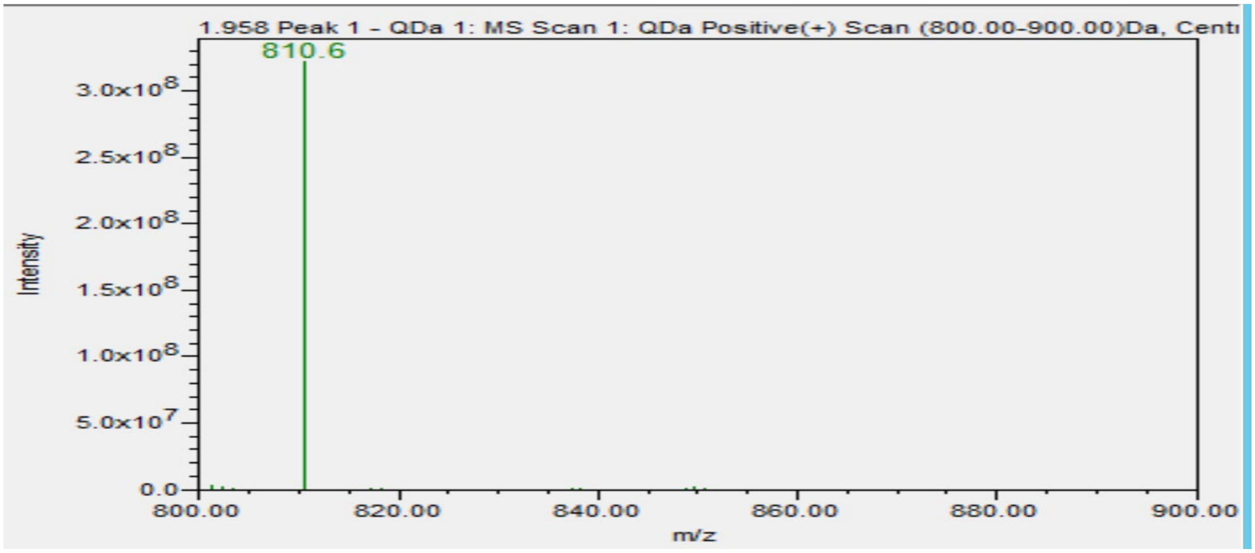

Figure S32: HPLC (top) and LCMS (bottom) Chromatograms for RV124

RV55: HPLC: Analytical HPLC trace of RV55 showing >98% purity. **LCMS:** Method: 2.1 x 100 mm Halo Peptide ES-C18, 2.7 µm, 2-40% in 20 min after 1 min hold, 0.4 mL/min, 50 °C, A = 0.05% TFA in H_2O, B = 0.05% TFA in ACN. LC-MS (ESI^+) calculated for $C_{78}H_{97}Cl_2N_9O_{26}$ $[M+H]^+$ *m/z*: 1645.5922; found: 1645.592; retention time = 16.5-16.9 minutes.

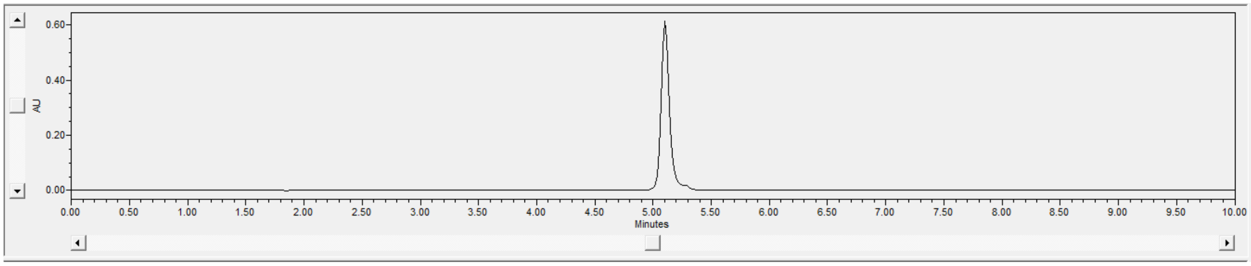

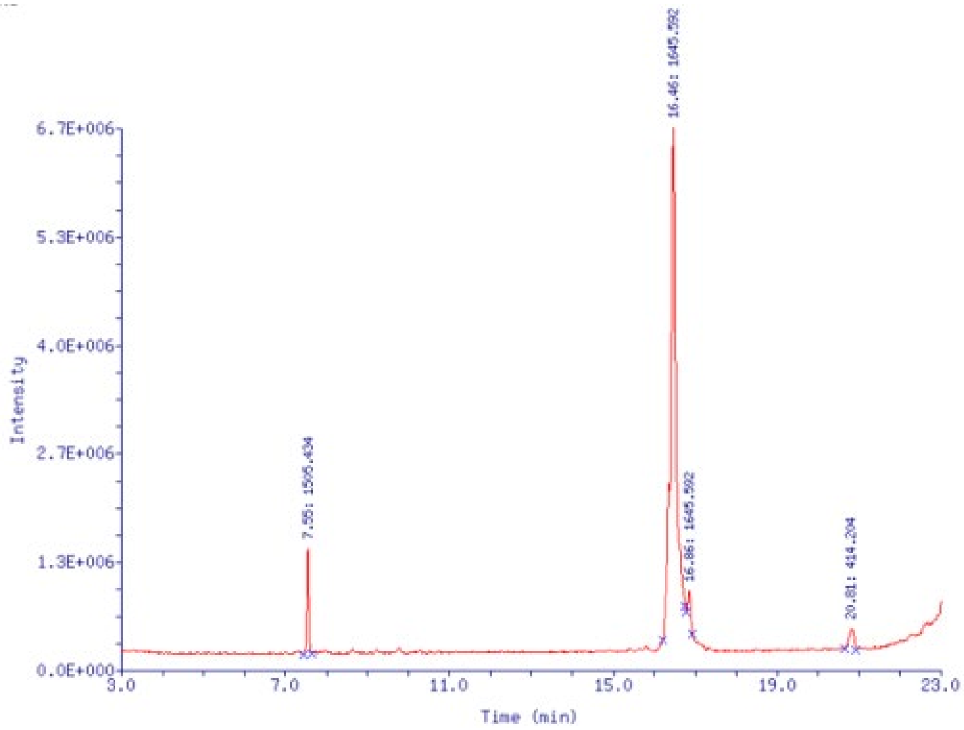

Figure S33: HPLC (top) and LCMS (bottom) Chromatograms for RV55

<u>RV126</u>: HPLC: Analytical HPLC trace of RV126 showing >90% purity. **LCMS:** Method: 2.1 x 100 mm Waters HILIC, 1.6 μm, 5-80% in 1.5 min after 1.5 min hold, 0.6 mL/min, 50 °C, A = 0.05% TFA in H_2O, B = 0.05% TFA in CAN; Waters QdA detector. LC-MS (ESI$^+$) calculated for $C_{80}H_{101}Cl_2N_9O_{26}$ $[M+2H]^{+2}$ *m/z*: 837.8 ; found: 838.0; retention time = 1.9 minutes.

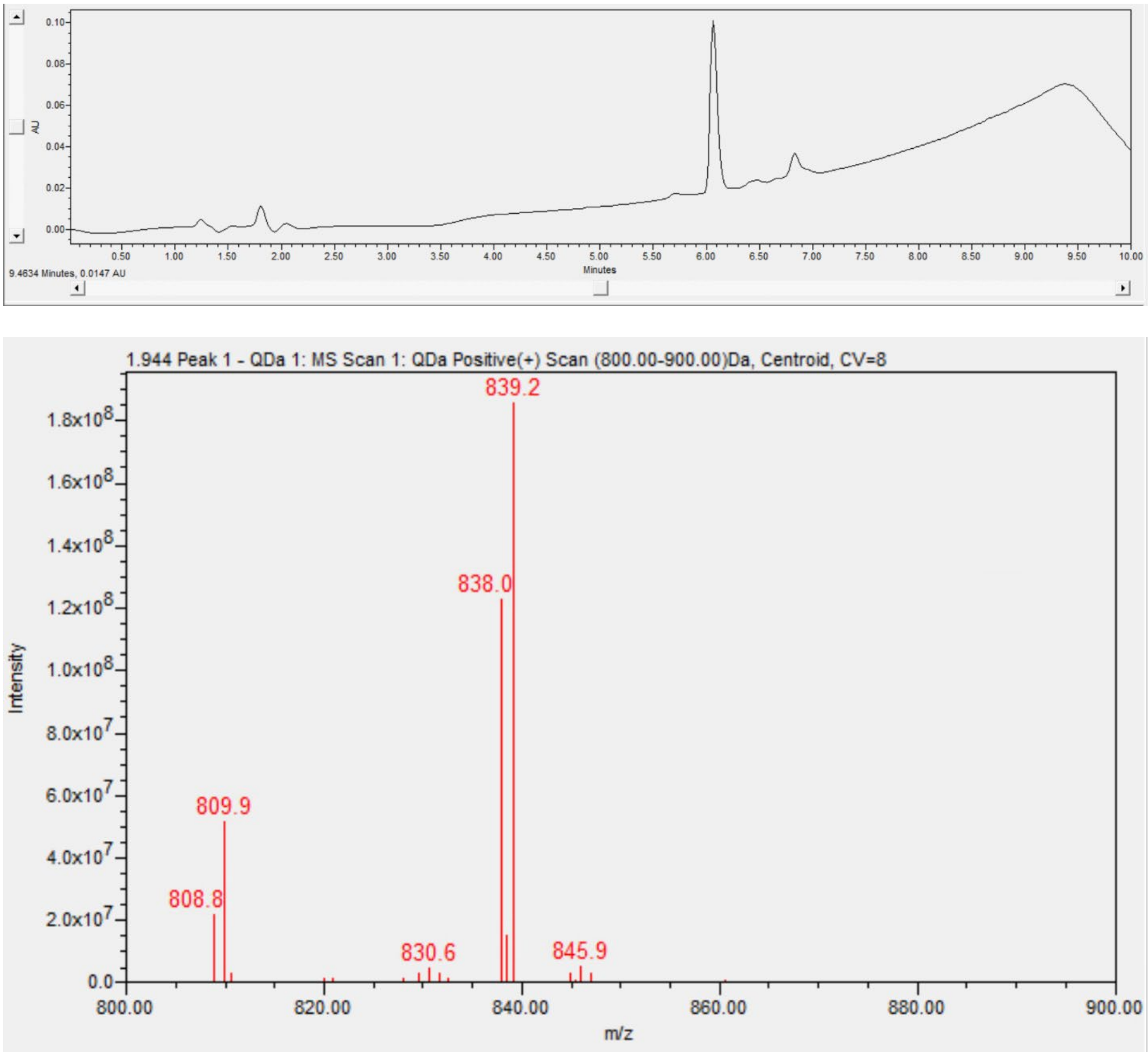

Figure S34: HPLC (top) and LCMS (bottom) Chromatograms for RV126

RV127: HPLC: Analytical HPLC trace of RV127 showing >98% purity. **LCMS:** Method: 2.1 x 100 mm Waters HILIC, 1.6 µm, 5-80% in 1.5 min after 1.5 min hold, 0.6 mL/min, 50 °C, A = 0.05% TFA in H_2O, B = 0.05% TFA in CAN; Waters QdA detector. LC-MS (ESI$^+$) calculated for $C_{82}H_{105}Cl_2N_9O_{26}$ $[M+2H]^{+2}$ *m/z*: 851.8 ; found: 852.7; retention time = 1.9 minutes.

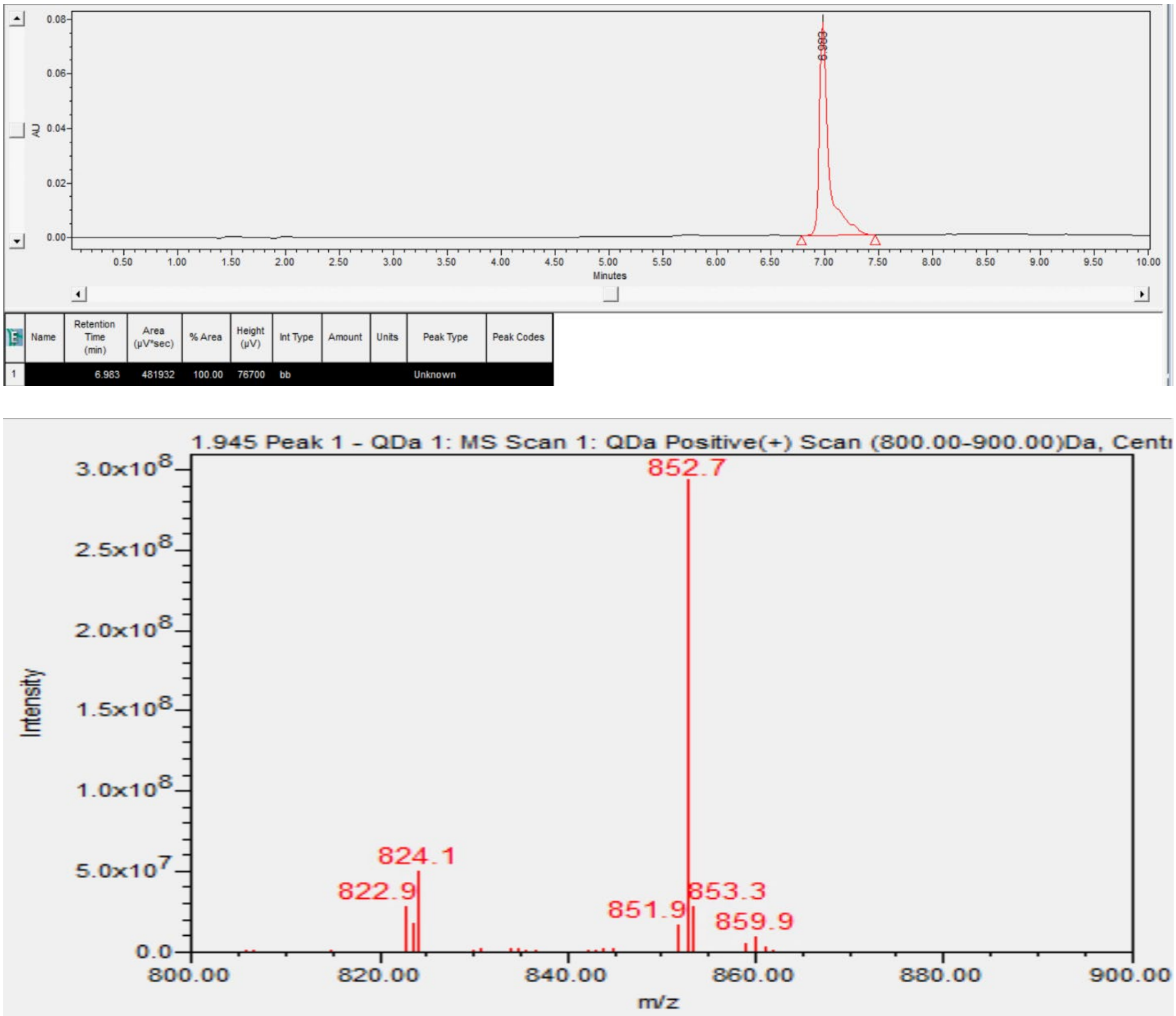

Figure S35: HPLC (top) and LCMS (bottom) Chromatograms for RV127

RV128: **HPLC**: Analytical HPLC trace of RV128 showing >98% purity. **LCMS:** Method: 2.1 x 100 mm Halo Peptide ES-C18, 2.7 µm, 2-40% in 20 min after 1 min hold, 0.4 mL/min, 50 °C, A = 0.05% TFA in H_2O, B = 0.05% TFA in ACN. LC-MS (ESI$^+$) calculated for $C_{84}H_{109}Cl_2N_9O_{26}$ $[M+H]^+$ *m/z*: 1729.6861; found: 1729.681; retention time = 17.5 minutes.

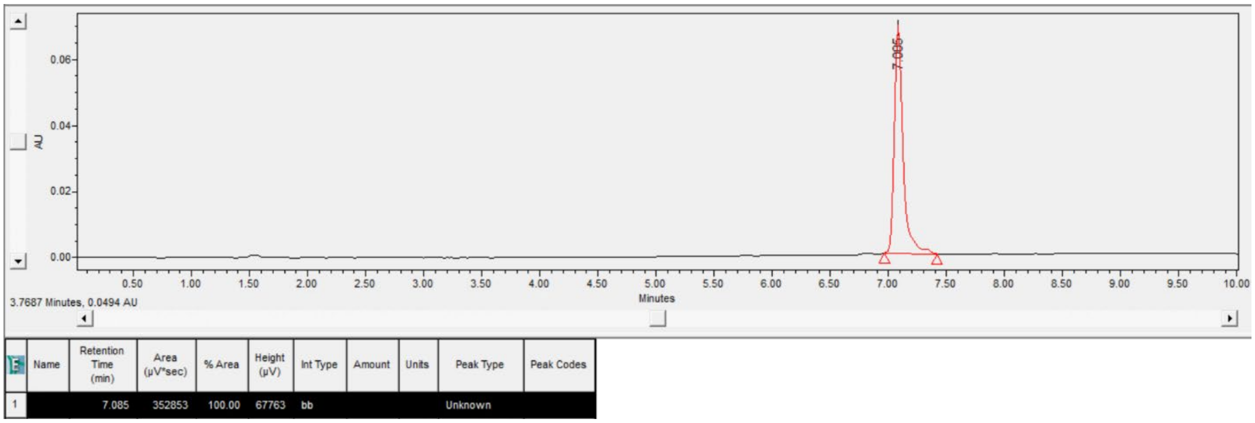

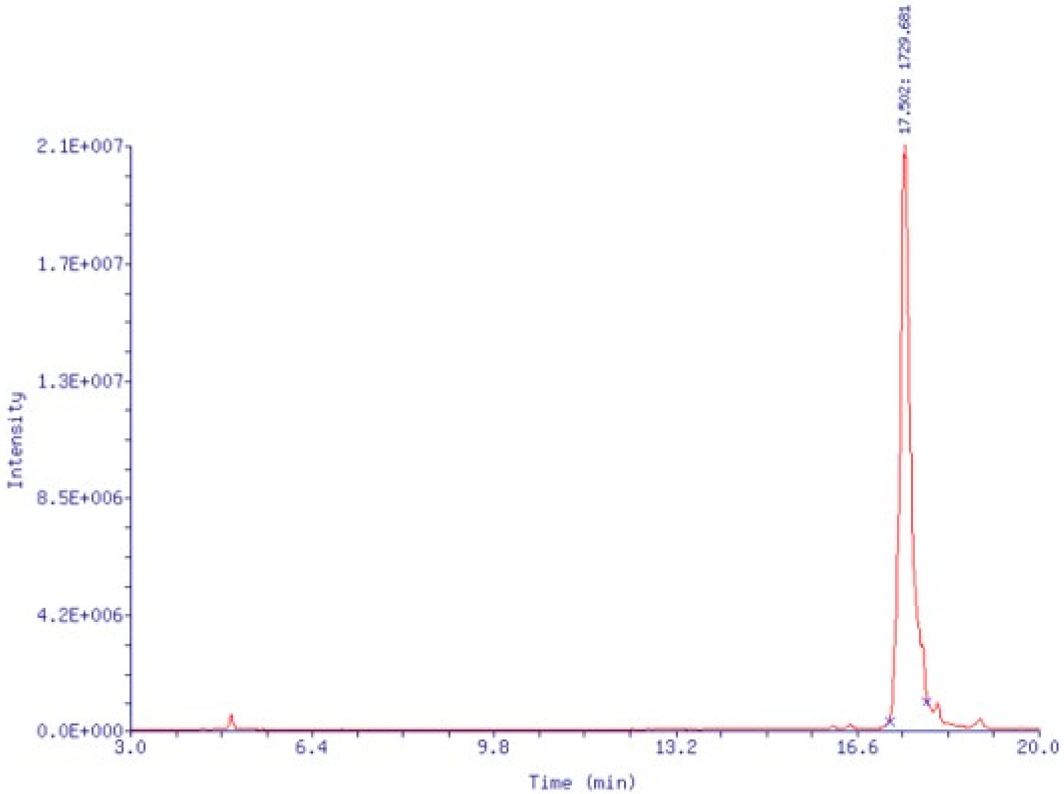

Figure S36: HPLC (top) and LCMS (bottom) Chromatograms for RV128

<u>RV101</u>: HPLC: Analytical HPLC trace of RV101 showing >98% purity. **LCMS:** Method: 2.1 x 100 mm Halo Peptide ES-C18, 2.7 µm, 2-40% in 20 min after 1 min hold, 0.4 mL/min, 50 °C, A = 0.05% TFA in H_2O, B = 0.05% TFA in ACN. LC-MS (ESI$^+$) calculated for $C_{68}H_{77}Cl_2N_9O_{26}$ [M+H]$^+$ *m/z*: 1505.4357; found: 1505.43; retention time = 8.7-9.0 minutes.

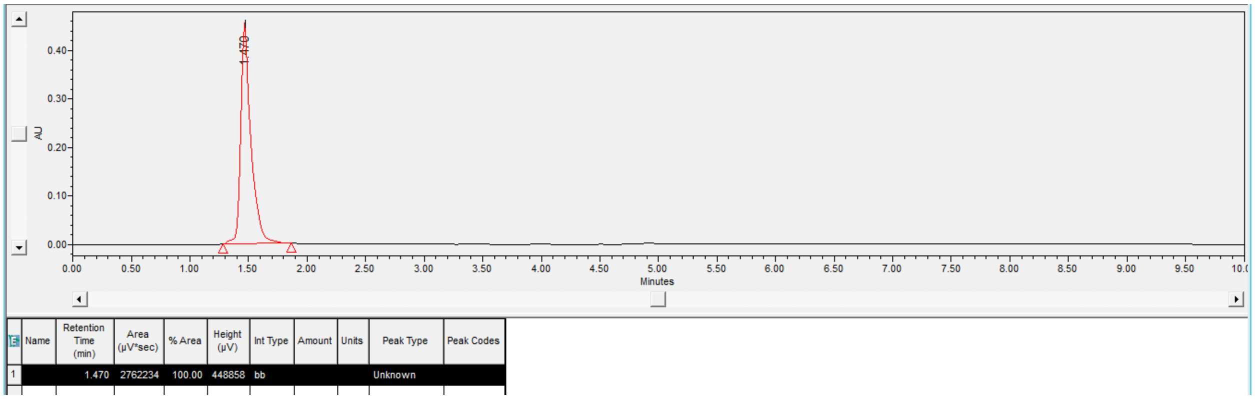

	Name	Retention Time (min)	Area (µV*sec)	% Area	Height (µV)	Int Type	Amount	Units	Peak Type	Peak Codes
1		1.470	2762234	100.00	448858	bb			Unknown	

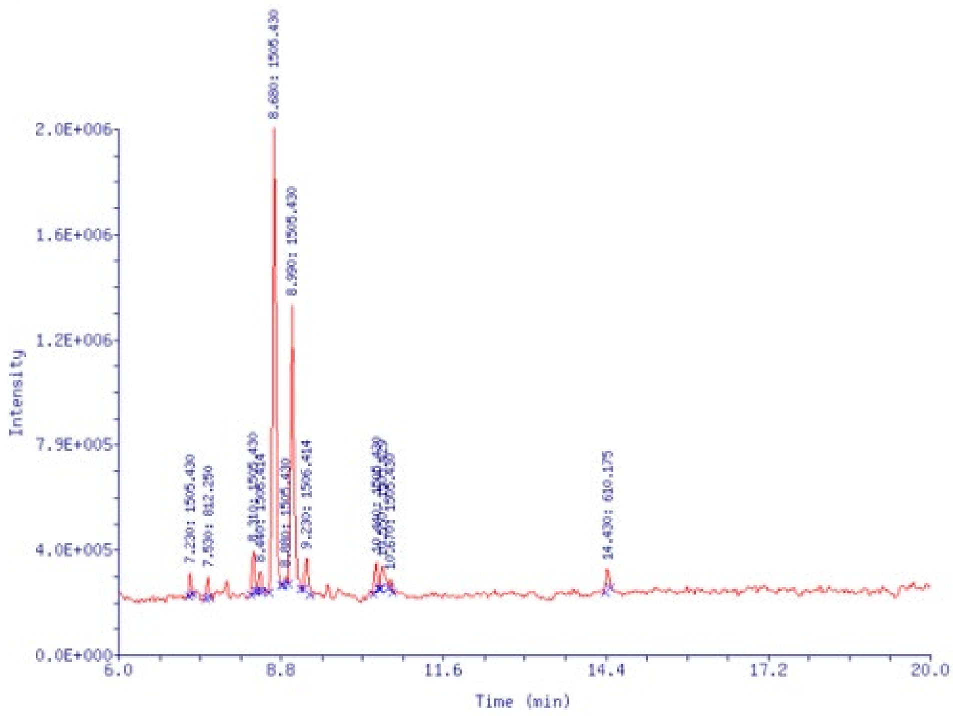

Figure S37: HPLC (top) and LCMS (bottom) Chromatograms for RV101

<u>RV93</u>: HPLC: Analytical HPLC trace of RV93 showing >98% purity. **LCMS:** Method: 2.1 x 100 mm Halo Peptide ES-C18, 2.7 µm, 2-40% in 20 min after 1 min hold, 0.4 mL/min, 50 °C, A = 0.05% TFA in H_2O, B = 0.05% TFA in ACN. LC-MS (ESI^+) calculated for $C_{76}H_{94}Cl_2N_{10}O_{25}$ $[M+H]^+$ *m/z*: 1616.5769; found: 1616.577; retention time = 13.6-13.9 minutes.

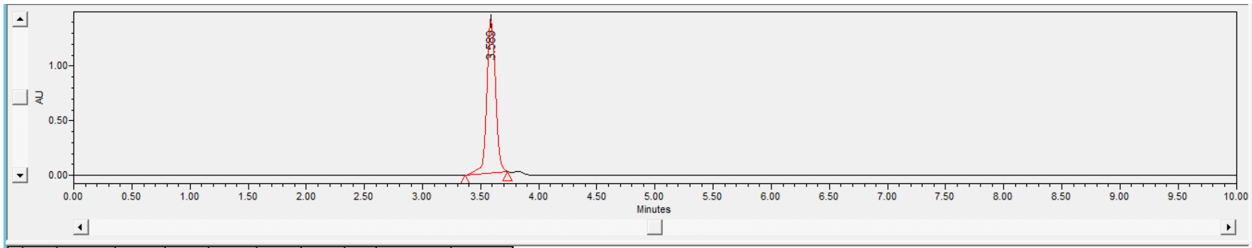

	Name	Retention Time (min)	Area (µV*sec)	% Area	Height (µV)	Int Type	Amount	Units	Peak Type	Peak Codes
1		3.589	7070102	100.00	1396346	bb			Unknown	

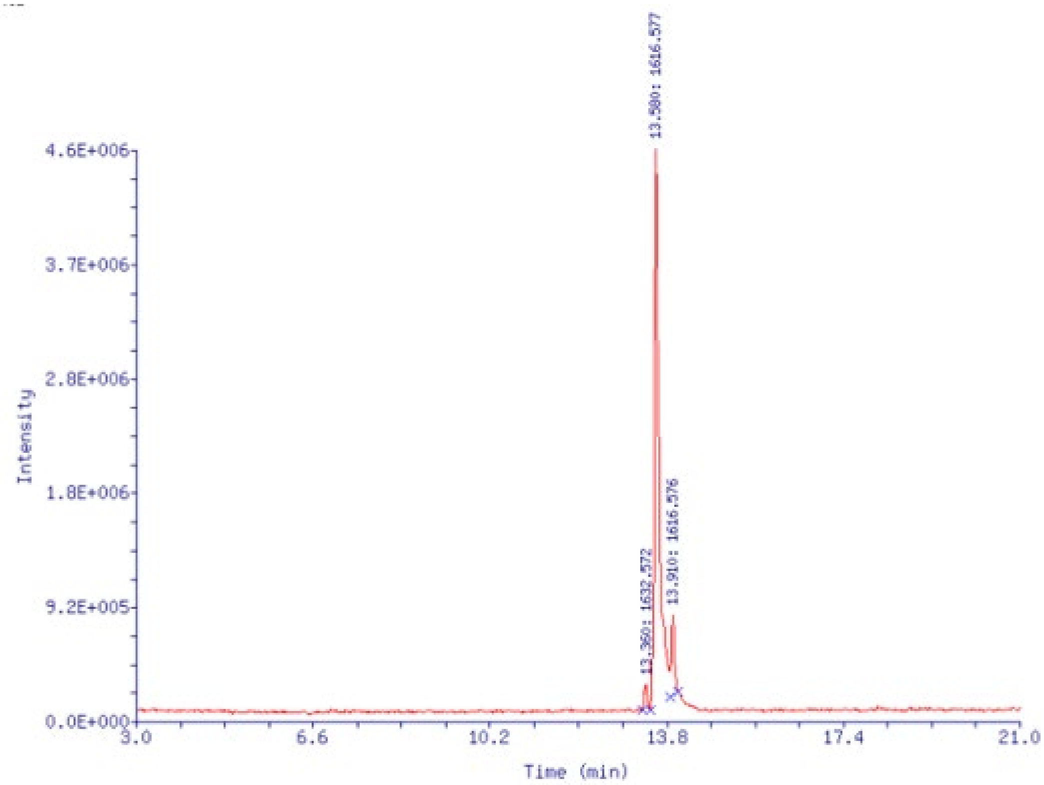

Figure S38: HPLC (top) and LCMS (bottom) Chromatograms for RV93

RV56: **HPLC**: Analytical HPLC trace of RV56 showing >98% purity. **LCMS:** Method: 2.1 x 100 mm Halo Peptide ES-C18, 2.7 µm, 2-40% in 20 min after 1 min hold, 0.4 mL/min, 50 °C, A = 0.05% TFA in H_2O, B = 0.05% TFA in ACN. LC-MS (ESI$^+$) calculated for $C_{78}H_{98}Cl_2N_{10}O_{25}$ $[M+H]^+$ *m/z*: 1644.6082; found: 1644.608; retention time = 14.2-14.5 minutes.

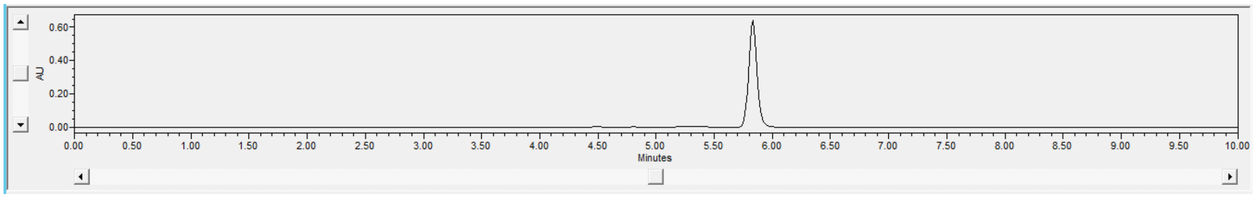

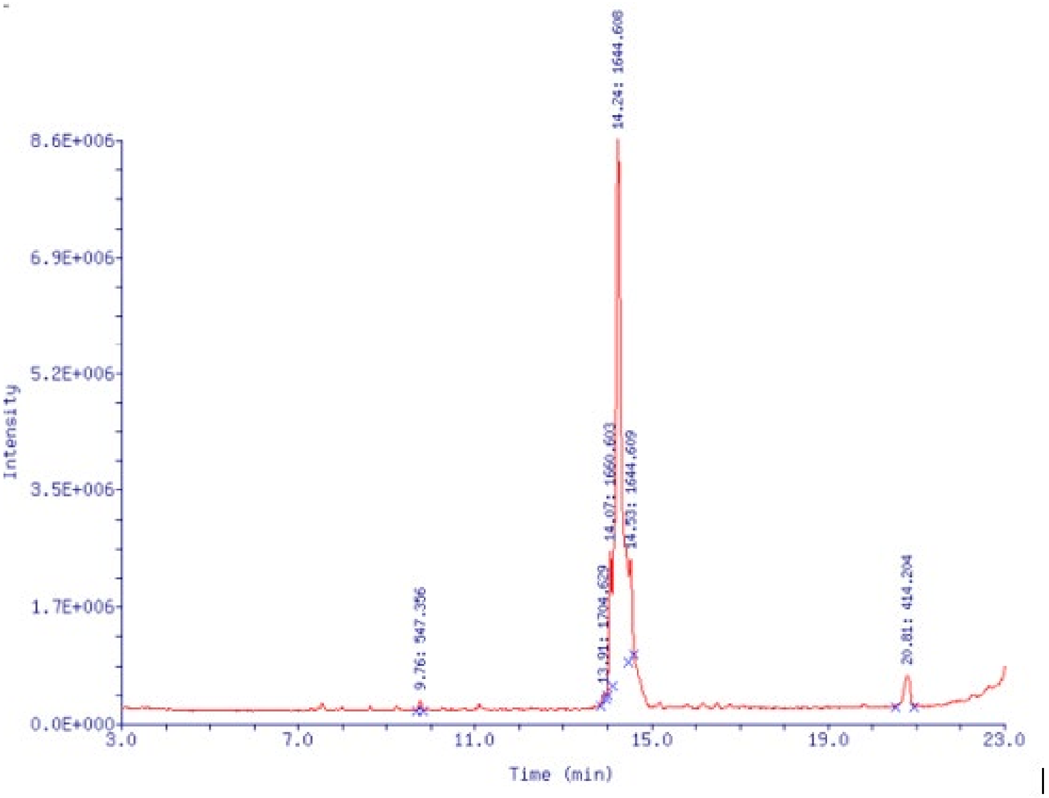

Figure S39: HPLC (top) and LCMS (bottom) Chromatograms for RV56

RV62: HPLC: Analytical HPLC trace of RV62 showing >98% purity. **LCMS:** Method: 2.1 x 100 mm Halo Peptide ES-C18, 2.7 µm, 2-40% in 20 min after 1 min hold, 0.4 mL/min, 50 °C, A = 0.05% TFA in H_2O, B = 0.05% TFA in ACN. LC-MS (ESI$^+$) calculated for $C_{80}H_{102}Cl_2N_{10}O_{25}$ $[M+H]^+$ *m/z*: 1672.6395; found: 1672.638; retention time = 19.2-19.4 minutes.

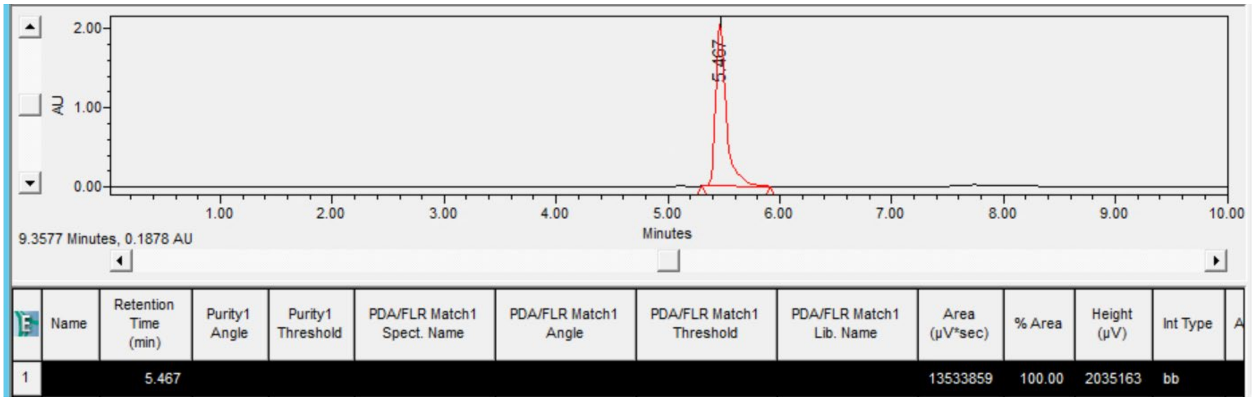

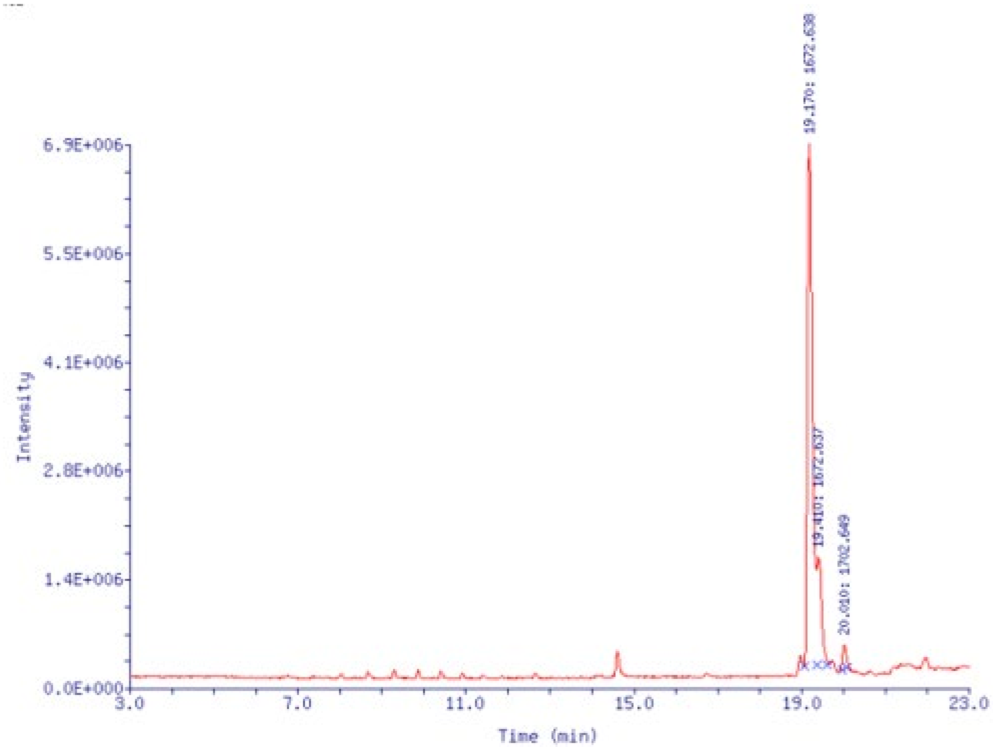

Figure S40: HPLC (top) and LCMS (bottom) Chromatograms for RV62

RV92: **HPLC**: Analytical HPLC trace of RV92 showing >98% purity. **LCMS:** Method: 2.1 x 100 mm Halo Peptide ES-C18, 2.7 µm, 2-40% in 20 min after 1 min hold, 0.4 mL/min, 50 °C, A = 0.05% TFA in H_2O, B = 0.05% TFA in ACN. LC-MS (ESI^+) calculated for $C_{82}H_{106}Cl_2N_{10}O_{25}$ $[M+H]^+$ *m/z*: 1700.6708; found: 1600.671; retention time = 21.7 minutes.

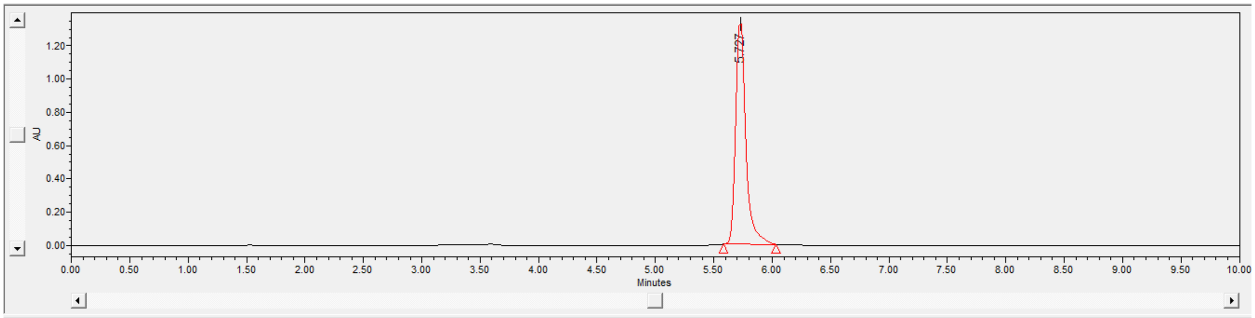

	Name	Retention Time (min)	Area (µV*sec)	% Area	Height (µV)	Int Type	Amount	Units	Peak Type	Peak Codes
1		5.727	7854182	100.00	1327539	bb			Unknown	

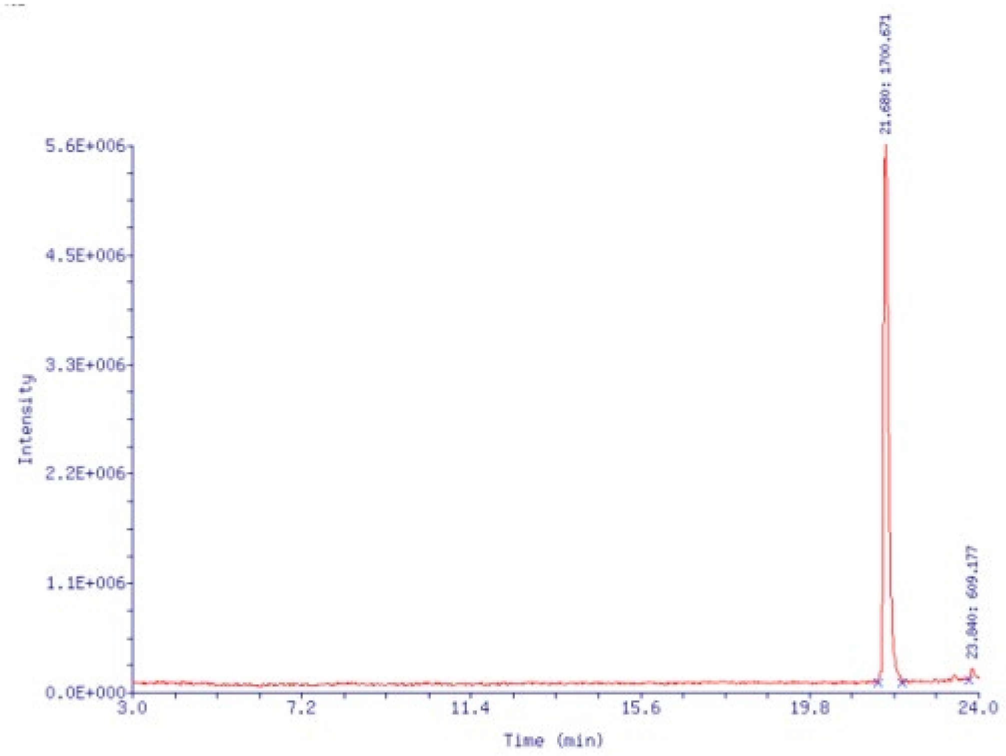

Figure S41: HPLC (top) and LCMS (bottom) Chromatograms for RV92

RV91: **HPLC**: Analytical HPLC trace of RV91 showing >98% purity. **LCMS:** Method: 2.1 x 100 mm Halo Peptide ES-C18, 2.7 µm, 2-40% in 20 min after 1 min hold, 0.4 mL/min, 50 °C, A = 0.05% TFA in H_2O, B = 0.05% TFA in ACN. LC-MS (ESI$^+$) calculated for $C_{84}H_{110}Cl_2N_{10}O_{25}$ $[M+H]^+$ *m/z*: 1728.7021; found: 1727.703; retention time = 22.4 minutes.

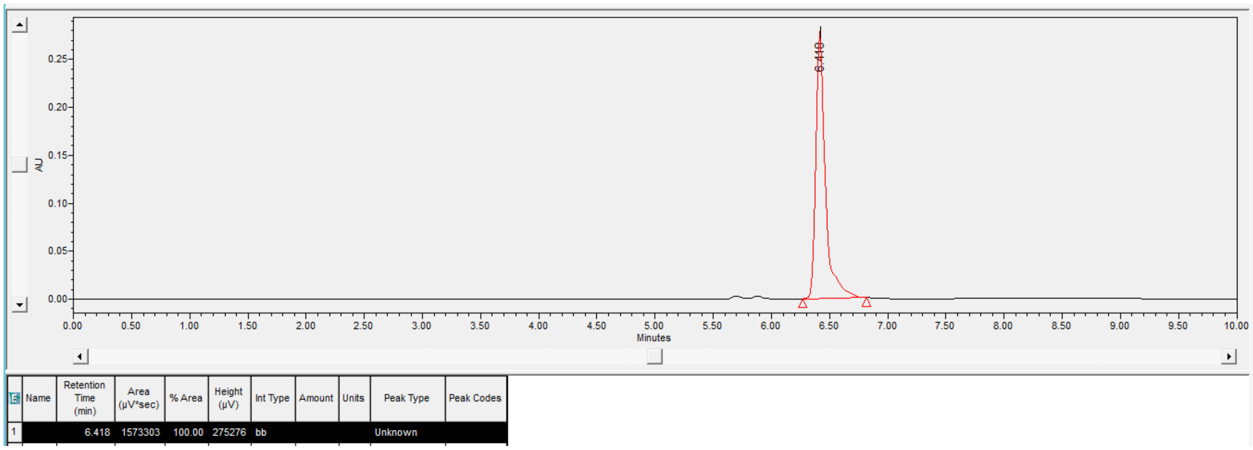

	Name	Retention Time (min)	Area (µV*sec)	% Area	Height (µV)	Int Type	Amount	Units	Peak Type	Peak Codes
1		6.418	1573303	100.00	275276	bb			Unknown	

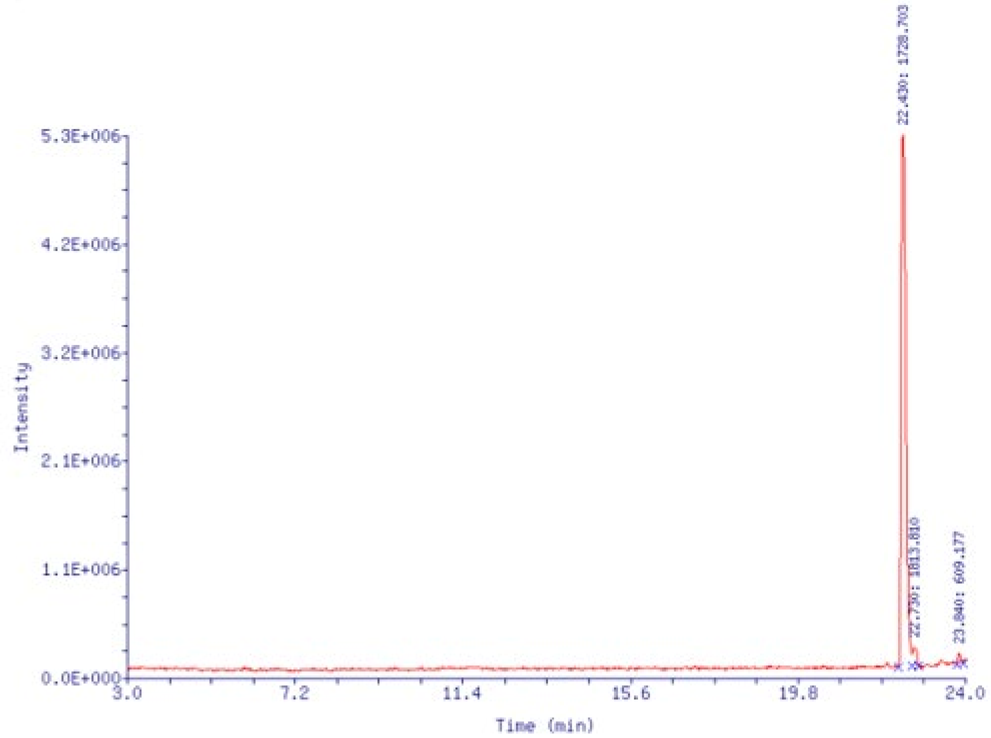

Figure S42: HPLC (top) and LCMS (bottom) Chromatograms for RV91

<u>RV82-FMOC</u>: HPLC: Analytical HPLC trace of RV82 showing >98% purity

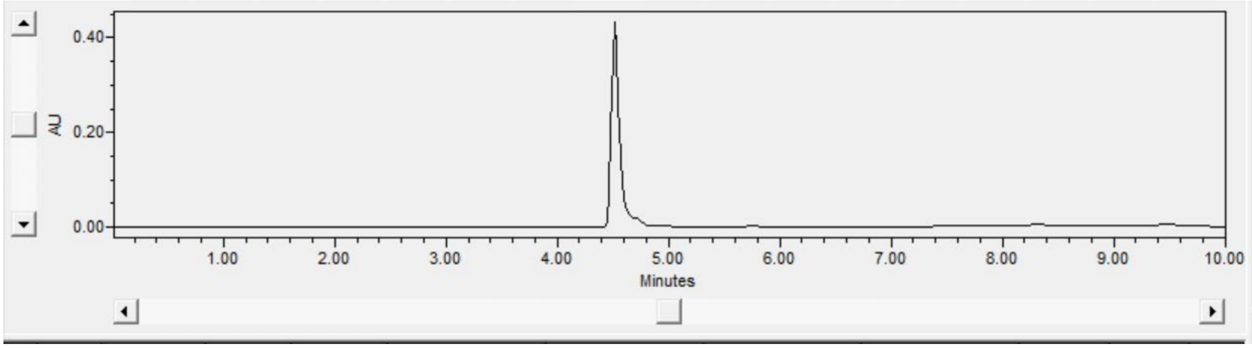

Figure S43: HPLC chromatograms for RV82-FMOC

<u>RV82</u>: HPLC: Analytical HPLC trace of RV82 showing >98% purity. **LCMS:** Method: 2.1 x 100 mm Halo Peptide ES-C18, 2.7 µm, 2-40% in 20 min after 1 min hold, 0.4 mL/min, 50 °C, A = 0.05% TFA in H_2O, B = 0.05% TFA in ACN. LC-MS (ESI^+) calculated for $C_{68}H_{80}Cl_2N_{10}O_{24}$ $[M+H]^+$ *m/z*: 1490.4724; found: 1490.478; retention time = 8.0-8.4 minutes.

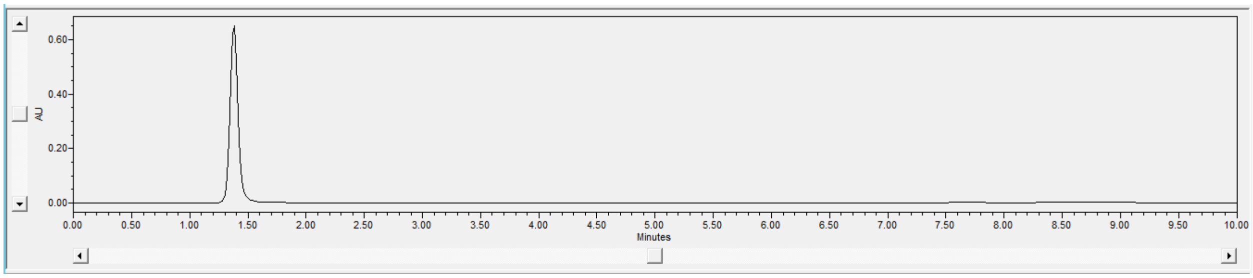

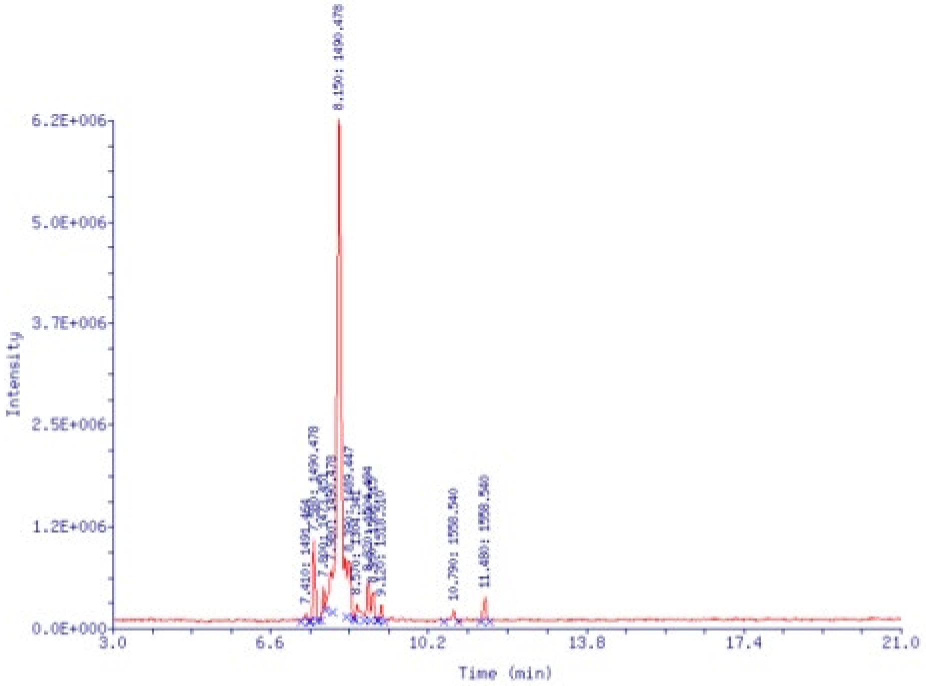

Figure S44: HPLC (top) and LCMS (bottom) Chromatograms for RV82

<u>RV121</u>: HPLC: Analytical HPLC trace of RV121 showing >98% purity. **LCMS:** Method: 2.1 x 100 mm Halo Peptide ES-C18, 2.7 µm, 2-40% in 20 min after 1 min hold, 0.4 mL/min, 50 °C, A = 0.05% TFA in H_2O, B = 0.05% TFA in ACN. LC-MS (ESI^+) calculated for $C_{76}H_{94}Cl_2N_{10}O_{25}$ $[M+H]^+$ *m/z*: 1616.5769; found: 1616.579; retention time = 15.7 minutes.

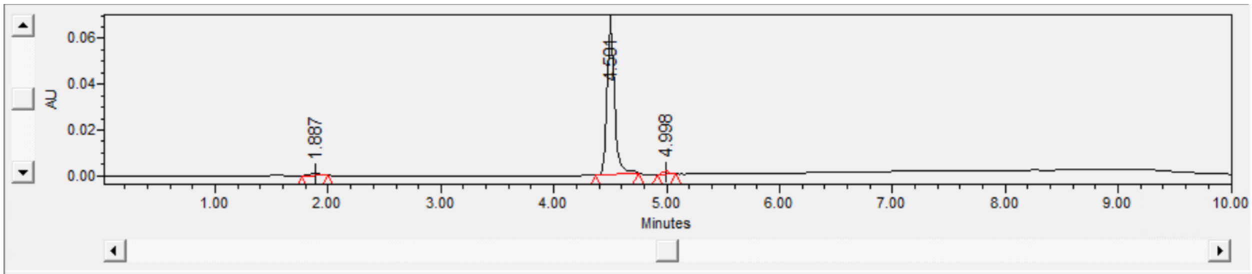

	Name	Retention Time (min)	Purity1 Angle	Purity1 Threshold	PDA/FLR Match1 Spect. Name	PDA/FLR Match1 Angle	PDA/FLR Match1 Threshold	PDA/FLR Match1 Lib. Name	Area (µV*sec)	% Area	Height (µV)	Int Type	Amo
1		1.887							7762	2.36	860	bb	
2		4.501							315365	96.01	64973	bb	
3		4.998							5331	1.62	1227	bb	

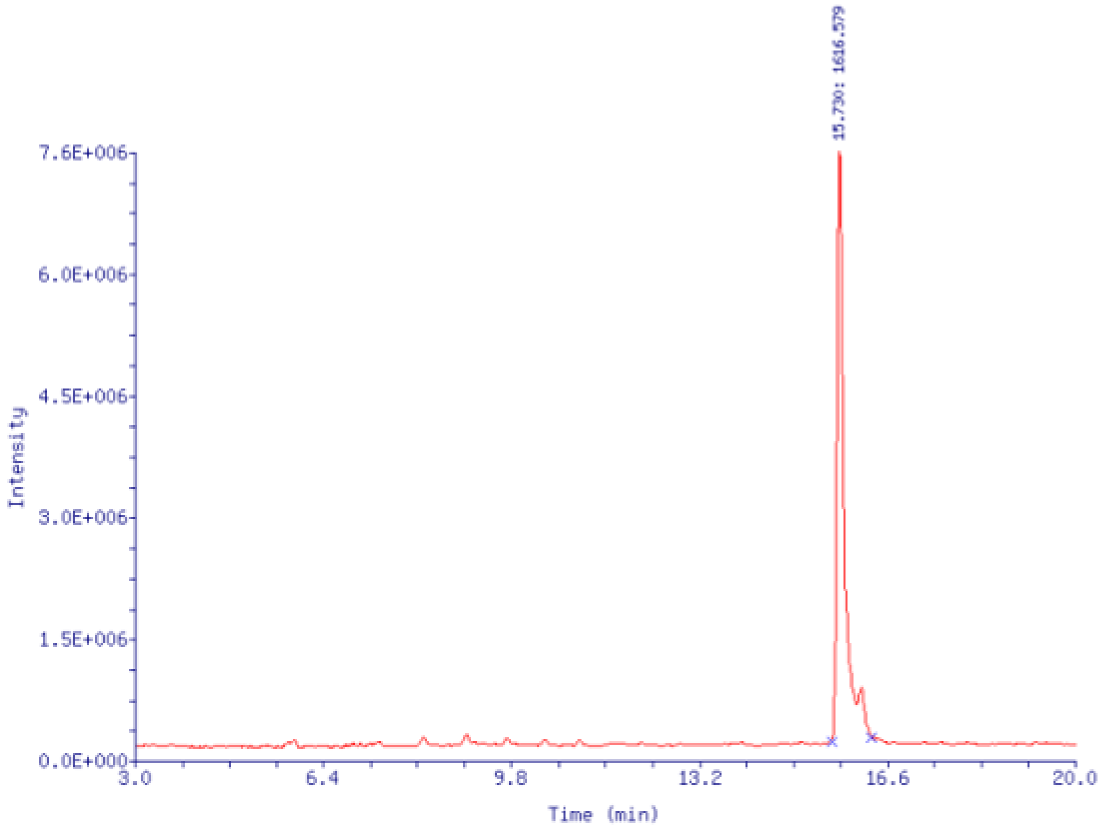

Figure S45: HPLC (top) and LCMS (bottom) Chromatograms for RV121

RV94: **HPLC**: Analytical HPLC trace of RV94 showing >98% purity. **LCMS:** Method: 2.1 x 100 mm Halo Peptide ES-C18, 2.7 µm, 2-40% in 20 min after 1 min hold, 0.4 mL/min, 50 °C, A = 0.05% TFA in H_2O, B = 0.05% TFA in ACN. LC-MS (ESI^+) calculated for $C_{78}H_{98}Cl_2N_{10}O_{25}$ $[M+H]^+$ *m/z*: 1644.6082; found: 1644.607; retention time = 18.4-18.8 minutes.

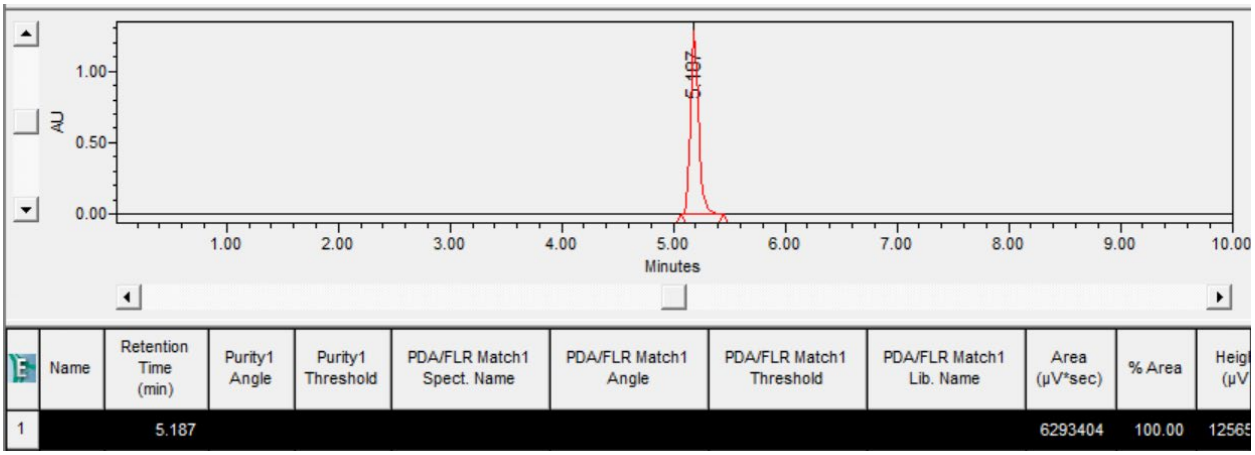

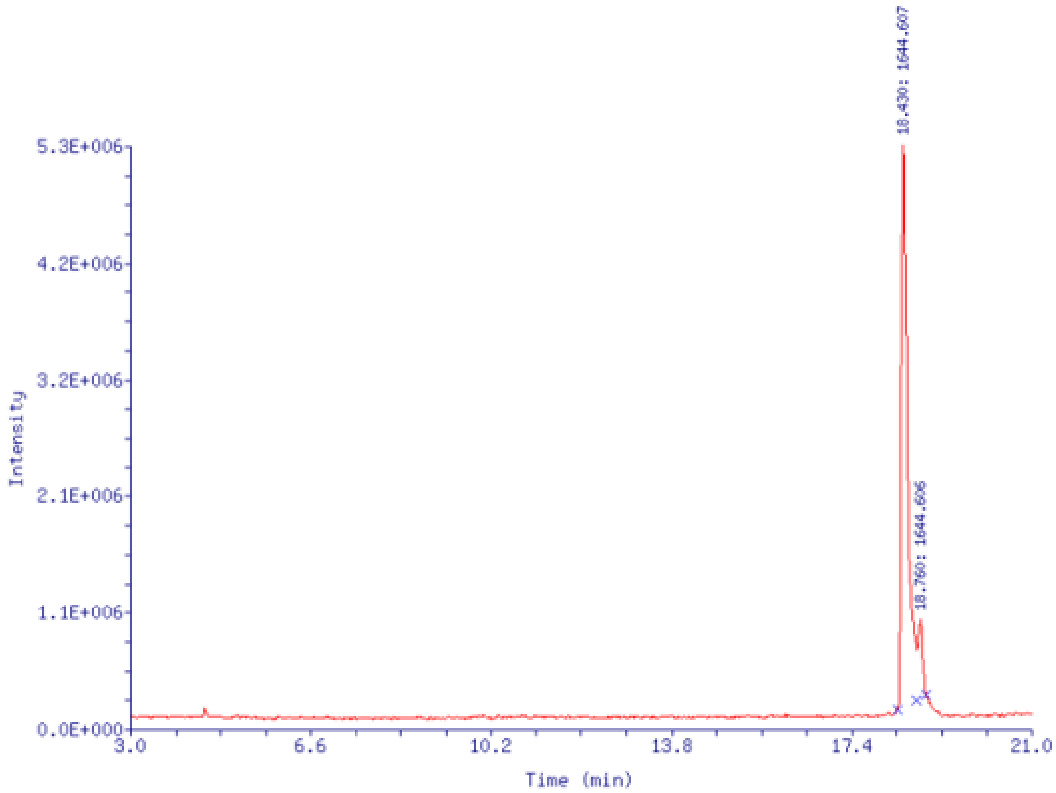

Figure S46: HPLC (top) and LCMS (bottom) Chromatograms for RV94

RV95: **HPLC**: Analytical HPLC trace of RV95 showing >98% purity. **LCMS:** Method: 2.1 x 100 mm Halo Peptide ES-C18, 2.7 µm, 2-40% in 20 min after 1 min hold, 0.4 mL/min, 50 °C, A = 0.05% TFA in H_2O, B = 0.05% TFA in ACN. LC-MS (ESI^+) calculated for $C_{80}H_{102}Cl_2N_{10}O_{25}$ $[M+H]^+$ *m/z*: 1672.6395; found: 1672.638; retention time = 21.1 minutes.

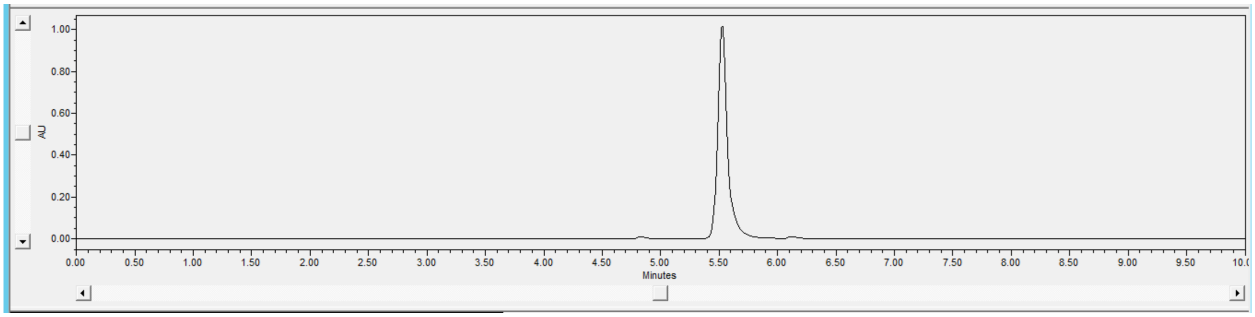

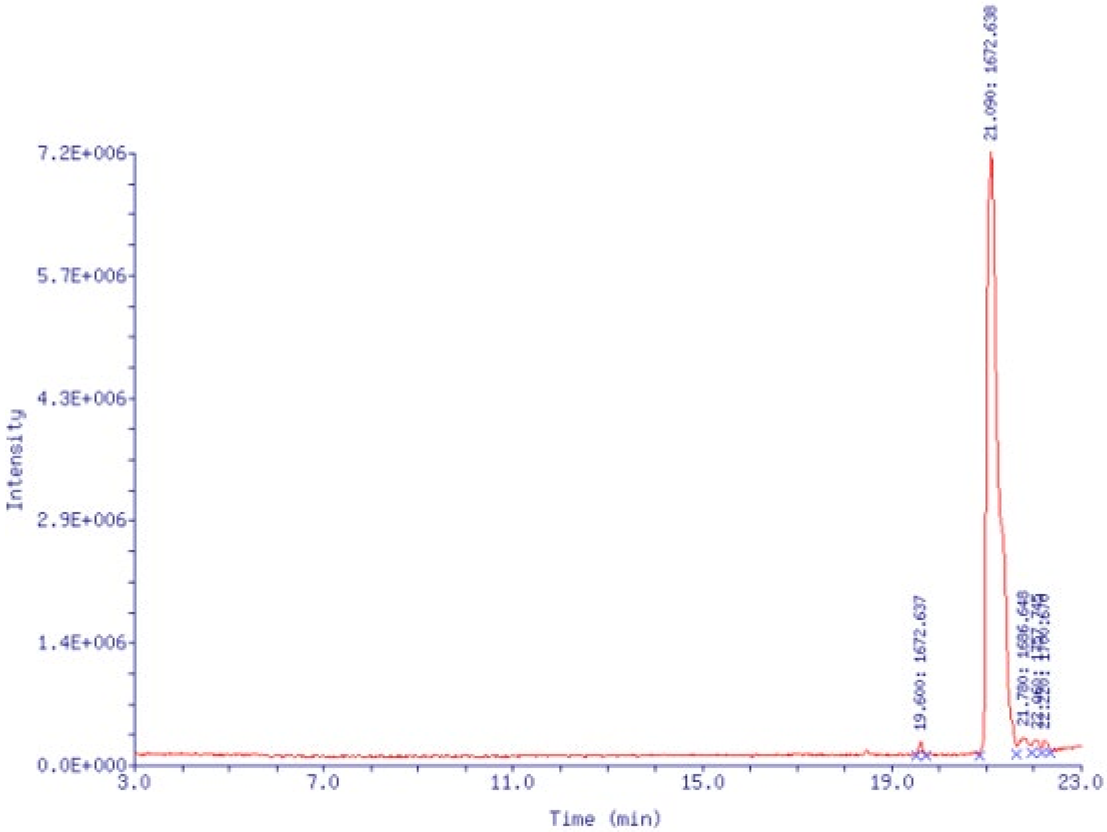

Figure S47: HPLC (top) and LCMS (bottom) Chromatograms for RV95

RV130: **HPLC**: Analytical HPLC trace of RV130 showing >98% purity. **LCMS:** Method: 2.1 x 100 mm Halo Peptide ES-C18, 2.7 µm, 2-40% in 20 min after 1 min hold, 0.4 mL/min, 50 °C, A = 0.05% TFA in H_2O, B = 0.05% TFA in ACN. LC-MS (ESI^+) calculated for $C_{82}H_{106}Cl_2N_{10}O_{25}$ $[M+H]^+$ *m/z*: 1700.6708; found: 1700.671; retention time = 15.3 minutes.

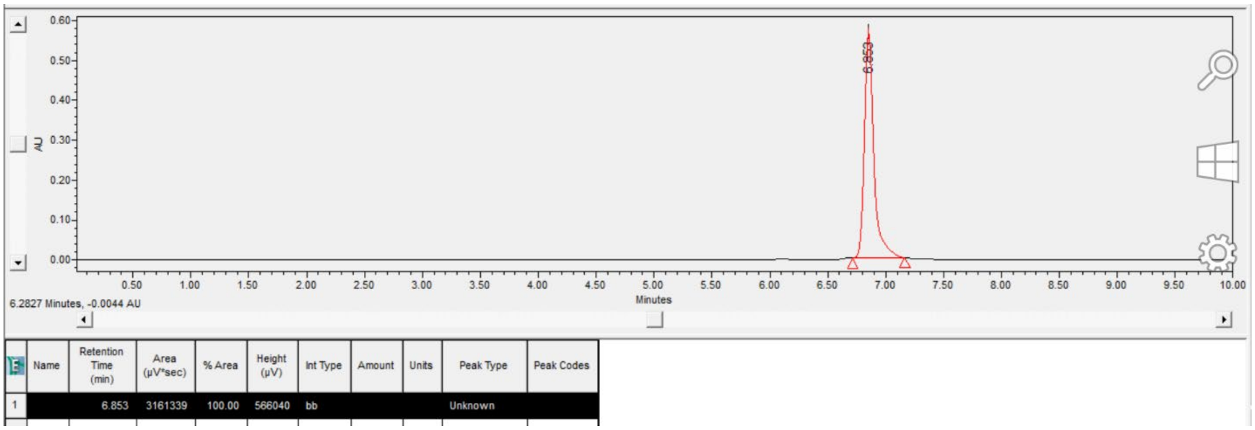

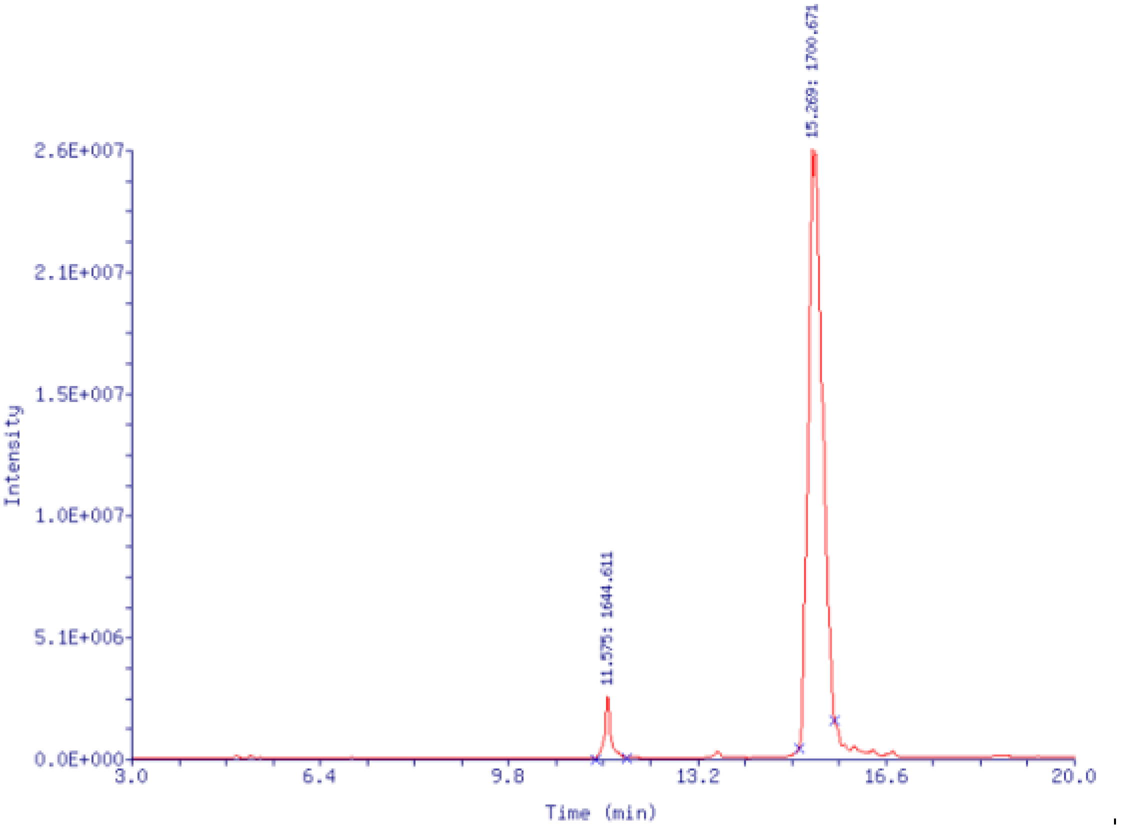

Figure S48: HPLC (top) and LCMS (bottom) Chromatograms for RV130

<u>RV131</u>: HPLC: Analytical HPLC trace of RV131 showing >98% purity. **LCMS:** Method: 2.1 x 100 mm Halo Peptide ES-C18, 2.7 µm, 2-40% in 20 min after 1 min hold, 0.4 mL/min, 50 °C, A = 0.05% TFA in H_2O, B = 0.05% TFA in ACN. LC-MS (ESI$^+$) calculated for $C_{84}H_{110}Cl_2N_{10}O_{25}$ $[M+H]^+$ *m/z*: 1728.7021; found: 1728.701; retention time = 17.2 minutes.

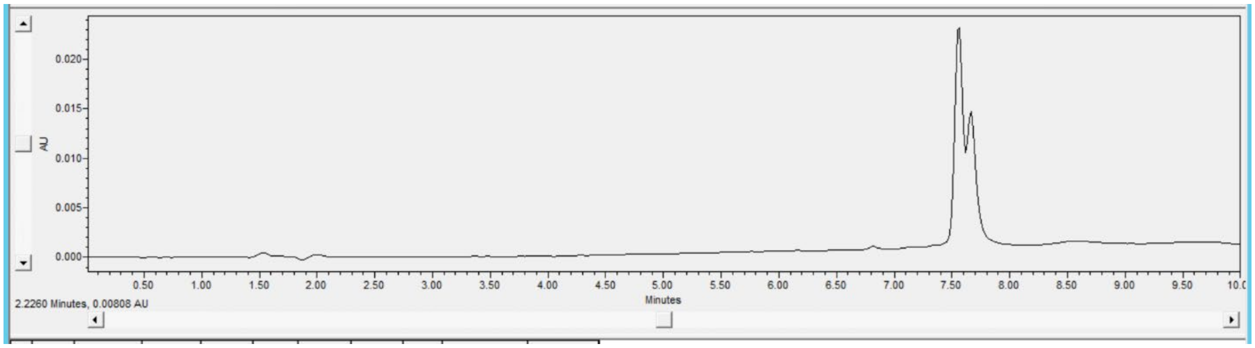

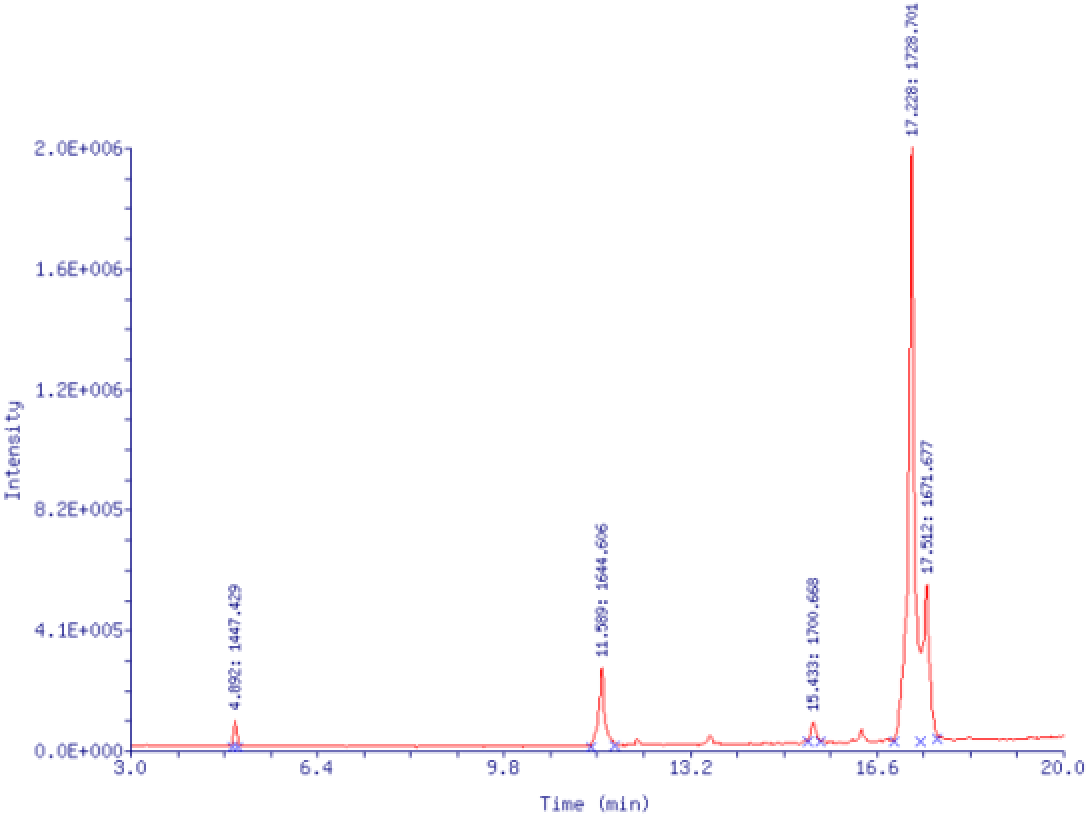

Figure S49: HPLC (top) and LCMS (bottom) Chromatograms for RV131

1. Leadbetter MR, Adams SM, Bazzini B, Fatheree PR, Karr DE, Krause KM, Lam BM, Linsell MS, Nodwell MB, Pace JL, Quast K, Shaw JP, Soriano E, Trapp SG, Villena JD, Wu TX, Christensen BG, Judice JK. 2004. Hydrophobic vancomycin derivatives with improved ADME properties: discovery of telavancin (TD-6424). J Antibiot (Tokyo) 57:326-36.
